# Probiotics for the prevention of mortality and sepsis in preterm very low birth weight neonates from low- and middle-income countries: a Bayesian network meta-analysis

**DOI:** 10.3389/fnut.2023.1133293

**Published:** 2023-06-14

**Authors:** Deena Thomas, Akash Sharma, M. Jeeva Sankar

**Affiliations:** ^1^Department of Pediatrics, Muthoot Hospitals, Kozhencherry, Kerala, India; ^2^Department of Pediatrics, Sir Padampat Institute of Neonatology and Pediatric Health (SPINPH), SMS Medical College, Jaipur, India; ^3^Department of Pediatrics, All India Institute of Medical Sciences, New Delhi, India

**Keywords:** network meta-analysis, neonate, very low birth weight, probiotics, enterocolitis, necrotizing, neonatal sepsis, neonatal mortality

## Abstract

**Background:**

Probiotics have been shown to reduce the risk of mortality and necrotizing enterocolitis (NEC) in very low birth weight (VLBW) neonates. The probiotic species with the maximal benefits in neonates from low- and middle-income countries are unknown.

**Objective:**

To identify the strain of probiotics with the maximum benefit in preventing neonatal mortality, sepsis, and NEC using the Bayesian network meta-analysis.

**Search methods:**

We searched Medline via PubMed, Embase, and Cochrane Central Register of Controlled Trials (CENTRAL). We also hand-searched reference lists of previous systematic reviews to identify eligible studies.

**Selection criteria:**

Randomized controlled trials (RCTs) from LMICs comparing enteral supplementation of one or more probiotics with another probiotic species or placebo were included.

**Data collection and analysis:**

Two authors screened the studies, extracted the data, and examined the risk of bias using the Cochrane risk of bias 2 (RoB 2) tools. Bayesian network meta-analysis was performed using the “BUGSnet” package in R and RStudio (version 1.4.1103). The confidence in the findings was assessed using the Confidence in Network Meta-analysis (CINeMA) web application.

**Results:**

Twenty-nine RCTs enrolling 4,906 neonates and evaluating 24 probiotics were included. Only 11 (38%) studies had a low risk of bias. All the studies compared the probiotics with a placebo; none had a head-to-head comparison of different probiotic species. Also, only one study each had evaluated most probiotic regimens. When compared to placebo, the combination of *B longum, B bifidum, B infantis*, and *L acidophilus* may reduce the risk of mortality (relative risk [RR] 0.26; 95% credible interval [CrI] 0.07 to 0.72), sepsis (RR 0.47; 95% CrI 0.25 to 0.83), and NEC (RR 0.31; 95% CrI 0.10 to 0.78) but the evidence is very uncertain. There is low certainty evidence that the single probiotic species, *B lactis*, could reduce the risk of mortality (RR 0.21; 0.05 to 0.66) and NEC (RR 0.09; 0.01 to 0.32).

**Conclusion:**

Given the low to very low certainty of evidence for the efficacy of the two probiotics found to reduce mortality and necrotizing enterocolitis, no firm conclusions can be made on the optimal probiotics for use in preterm neonates in low- and middle-income countries.

**Systematic review registration:**

https://www.crd.york.ac.uk/prospero/display_record.php?ID=CRD42022353242, identifier: CRD42022353242.

## Introduction

Globally, about 5 million children die before reaching their fifth birthday every year. Roughly half of these under-five child deaths occur during the neonatal period—the first 28 days of life. The risk of neonatal deaths is not uniform across the countries—it varies from 1 per 1,000 live births to 44 per 1,000 live births. The mortality rate is highest in the countries from sub-Saharan Africa and Southern Asia region; a child born in sub-Saharan Africa is about ten times more likely to die during the neonatal period than a child born in a high-income country (1).

The two most common causes of neonatal deaths in low- and middle-income countries (LMIC) are preterm birth complications and neonatal sepsis (2). Various interventions, including antenatal corticosteroids (3) and kangaroo mother care (4, 5), have been shown to reduce neonatal mortality and sepsis in preterm neonates. Increased coverage of the proven interventions during labor, birth, and postnatal period would avert up to 70% of neonatal deaths in LMICs (6). Efforts are underway to address the coverage of interventions and improve the quality of care in the facility-based care of neonates in LMIC settings. Concurrently, it is critical to identify other evidence-based interventions to reduce neonatal mortality secondary to prematurity and sepsis.

Probiotics have emerged as a promising intervention to prevent necrotizing enterocolitis (NEC) and mortality in preterm very low birth weight (VLBW) neonates. The Cochrane Review (2020), which included 56 trials involving 10,812 neonates, concluded that probiotics might reduce the risk of NEC, mortality, and sepsis. However, the sensitivity analysis of 16 studies at low risk of bias did not show any effect on mortality or sepsis (7). Another meta-analysis assessing the efficacy and safety of probiotics in LMICs in 2017 showed a reduction in all-cause mortality, sepsis, and NEC among 2000 enrolled neonates (8). Around 2,000 additional neonates have been enrolled in probiotic trials in LMICs in the last 5 years, which mandates an update of the available evidence.

While the beneficial effects of probiotics—as a group—are known, it is still unclear which probiotic species, alone or in combination, provides the maximum benefits in reducing mortality or NEC. A few network meta-analyses involving studies from high-income countries and LMICs have examined this issue. But they have predominantly evaluated the probiotics' effect at the genus level, not at the species/strain level. Assessing the impact of individual species/strains of probiotics is critical in LMICs, given the potentially different maternal and neonatal microbiomes in these settings (9, 10). Differences in maternal gut microbiota, environmental flora, and nature of antibiotic use in mothers and neonates could substantially affect the effects of the individual probiotic species in LMIC settings. We, therefore, conducted this Bayesian species-specific network meta-analysis to examine the efficacy of different probiotic species on (1) all-cause mortality, (2) culture-positive sepsis, and (3) NEC in very low birth weight neonates or neonates <32 weeks of gestational age at birth.

## Methods

### Search strategy

We followed the Preferred Reporting Items for Systematic Reviews and Meta-Analyses (PRISMA) (11) statements and Cochrane Handbook for Systematic Reviews of Interventions (12). The study protocol was prospectively registered with PROSPERO (CRD42022353242). Deviations from the published protocol have been mentioned in Supplementary Table 1.

We searched MEDLINE (via PubMed), Cochrane Central Register of Controlled Trials (CENTRAL), and Embase from inception to July 31, 2022. The search strategy was developed by two reviewers (DT and AS) and finalized by the third reviewer (MJS). The following keywords were used to build our search strategy: intervention and control—probiotic^*^/prebiotic^*^/synbiotic^*^/placebo; population—*infant, newborn, OR preterm*; study design—*randomized controlled trials*. In addition, we also searched the references of previous systematic reviews and meta-analyses. No language restrictions were used during the literature search. Only studies from low- and middle-income countries (13) were identified and included in the final analysis. The detailed search strategy has been described in Supplementary Table 2.

### Study selection

Randomized controlled trials (RCT) or quasi-RCT comparing (1) enteral supplementation of one or more species of probiotics with another probiotic species/genera and (2) supplementation of any probiotics with placebo or no probiotics in very low birth weight (VLBW) neonates were considered eligible for this review (14). Two researchers (DT and AS) independently conducted the title and abstract screening—using the Covidence web application (14)—followed by full-text screening to determine eligibility. The disagreements were resolved by mutual discussion or discussion with the third author (MJS). Studies that met the following criteria were finally included: (i) *population:* birthweight of enrolled neonates <1,500 g or gestation <32 weeks. Studies that enrolled more mature or heavier neonates were also included if the mean gestation of the neonates was <32 weeks or birth weight was <1.5 kg. If the gestation/birthweight data was unavailable, at least 50% of neonates must have been born before 32 weeks or have a birth weight of <1.5 kg to be eligible; (ii) *intervention:* one or more species of probiotics; (iii) *comparator:* a different species of probiotics or placebo or no probiotics; (iv) *outcome:* neonatal mortality, sepsis/severe infection, and necrotizing enterocolitis stage 2 or more as per modified Bell's staging (15); and (v) *others:* conducted in LMICs wherein LMIC was defined as per world bank data as countries with gross national income per capita less than $4,256 (13). We excluded cross-over trials and studies that employed prebiotics or synbiotics as cointerventions (along with the probiotics) or had not reported at least one of the three primary outcomes.

### Primary and secondary outcomes

Primary outcomes were all-cause neonatal mortality and sepsis/severe infection at discharge or 28 days or the latest follow-up. Sepsis was identified by a positive culture of bacteria or fungus from blood, cerebrospinal fluid, urine, or from a normally sterile body space or as defined by the authors of the individual studies. The secondary outcome was necrotizing enterocolitis (NEC)—stage 2 or more as per modified Bell's staging (15).

### Data extraction

Two reviewers (DT and AS) extracted the data of key demographic characteristics and outcomes from the included studies and collated them in a predesigned master spreadsheet. Data collected included general article information (author, study ID, and language of trial), trial information (type, location, setting, size sample, treatment arms, and randomization), demographic information of participants (gestation and birth weight), characteristics of interventions (number of treatment arms, intervention and control groups, and timing, dose, and route of administration), outcomes (primary and secondary outcomes), and risk of bias (sequence generation, allocation concealment, blinding, selective reporting, and incomplete data). The third reviewer (MJS) checked the master spreadsheet for the accuracy of extracted data.

### Risk of bias and certainty of evidence

Two reviewers (DT and AS) independently assessed the risk of bias of each study using Cochrane's “Risk-of-bias” 2.0 (RoB 2) tool (16); any discrepancy was resolved by discussion with the third reviewer (MJS). The “*robvis”* package in R (17) was used to create the summary and traffic-light plots of the risk-of-bias summary assessment for each outcome. We used the CINeMA (Confidence in Network Meta-Analysis) web application to examine the confidence in the findings from the network meta-analysis (18, 19). CINeMA considers six domains: within-study bias, reporting bias, indirectness, imprecision, heterogeneity, and incoherence. We first evaluated the direct evidence from pairwise comparisons on these domains. Then we intended to assess the certainty of indirect evidence from the lowest quality of direct evidence (of pairwise comparisons) from the first-order loops. The certainty of the evidence was ranked as high, moderate, low, and very low based on the presence or absence of “major concerns” in 0, 1, 2, or 3 (or more), respectively, of the six domains mentioned above.

### Statistical analysis

We used Stata version 15.1 (StataCorp, College Station, TX) for data preparation and analysis. For each outcome, we conducted a Bayesian network meta-analysis (NMA) by fitting a generalized linear model with a complementary log-log link function and binomial likelihood function using the “BUGSnet” (Bayesian inference Using Gibbs Sampling to conduct a Network meta-analysis) package (20) in R and RStudio (version 1.4.1103). We performed the Bayesian analysis with Markov chain Monte Carlo simulation using vague priors. We specified a burn-in of 50,000 iterations followed by 100,000 iterations with 10,000 adaptations, consistent with the NICE-DSU technical support document (21). Leverage plots, total residual deviance, and deviance information criterion were employed to assess the model fit. Gelman-Rubin and trace and density plots were inspected for model convergence. We intended to use the node-splitting and inconsistency model method to look for any inconsistency between the direct and indirect evidence (22).

Network diagrams were generated for each outcome wherein the node size and the line width represent the number of neonates and the number of trials for different comparisons, respectively. Posterior medians of relative risks (RR) and 95% credible intervals (CrI) were used to express the effect size. Forest and league plots were used to depict the network estimates of different comparisons. We calculated the mean surface under the cumulative ranking (SUCRA) curve for each intervention arm. In addition, we planned to do a subgroup analysis on the effect of probiotics on the two primary outcomes based on the type of feeding—exclusive breastmilk feeds, exclusive formula feeding, or mixed feeding.

## Results

### Study selection

**Figure 1:**
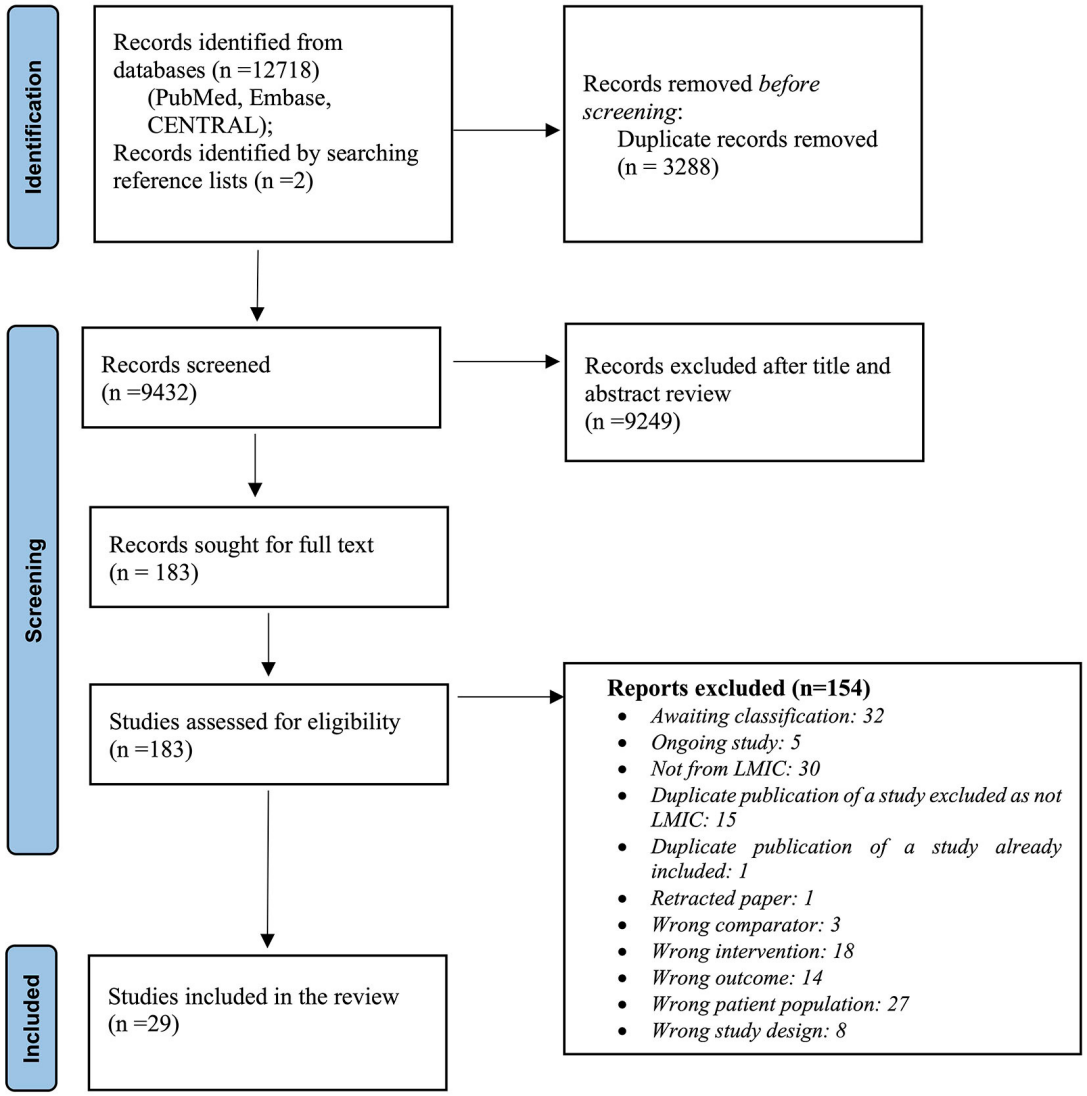
Flow chart of search results (adapted from PRISMA 2009 flow diagram).

Figure 1 depicts the process of screening and selection of eligible studies. Of the 183 full-text articles assessed for eligibility, 29 randomized and quasi-randomized studies from low- and middle-income countries enrolling 4,906 neonates were included in the review (Table 1). A detailed list of the excluded studies, those awaiting classification, and ongoing studies have been provided in Supplementary Tables 3–5, respectively.

**Table 1:** Baseline characteristics of included studies.

**S No.**	**References**	**Country**	**Inclusioncriteria**	**Gestation, weeks; mean (SD)**		**Birthweight, grams; mean (SD)**		**Sample size**	**Intervention**			**Control**	**Postnatal age at randomization (days)**		**Proportion of infants on exclusive breastmilk feeding**		**Critical outcomes reported**	**Mode of feeding**	**Duration of follow up **
				**P**	**C**	**P**	**C**		**Probiotic species**	**Duration of treatment**	**Dosage**		**P**	**C**	**P**	**C**			
1.	Braga et al. (23)	Brazil	750–1,500 g	29.5 (2.5)	29.2 (2.6)	1,195 (206)	1,151 (225)	231	*B breve + L casei* (*n* = 119)	Till 30 days of life/diagnosis of NEC/discharge or death, whichever occurred earlier	35 × 10^6^ to 3500 × 10^6^ CFU OD	None (*n* = 112)	2	2			Mortality, sepsis, NEC	Both HM and F	30 days
2.	Chandrashekar et al. (24)	India	Clinically stable ≤ 34 weeks	-	-	-	-	140	*B longum + L acidophilus + L rhamnosus + Sa boulardii* (*n =* 70)	Minimum of 7 days or till 35 weeks of gestation	1250 × 10^6^ CFU OD	None (*n =* 70)			74.3	81.4	Mortality, sepsis, NEC	Both HM and F	Duration of hospital stay: 15 days for probiotic and 23 days for control
3.	Chowdhury et al. (25)	Bangladesh	<33 weeks; <1,500 g; age >48 h	31.4 (0.9)	31.7 (0.8)	1,311 (110)	1,338 (98)	119	*B bifidum + B longum + B infantis + L rhamnosus + L paracasei + L casei + L acidophilus + L lactis* (*n =* 60)	Till discharge (minimum 10 days)	3000 × 10^6^ CFU OD	None (*n =* 59)	3.3	3.4			Mortality, NEC	Both HM and F	Duration of stay: 16 days in study grp vs 19 days in control group
4.	Dashti et al. (26)	Iran	700–1,800 g; stable and able to have enteral feeding	31.1 (2.68)	31.4 (2.6)	1,373 (279)	1,441 (253)	136	*B longum + B breve + L acidophilus + L rhamnosus + L bulgaricus + L casei + S thermophilus* (*n =* 69)	Information not available	750 × 10^6^ CFU OD; birthweight < 1000 g: 500 × 10^6^ CFU OD	Placebo (*n =* 67)	4.5 (3.4)	4.2 (2.9)	42.6	27.3	Mortality, sepsis, NEC	Both HM and F	Duration of stay 27-28 days
5.	Demirel et al. (27)	Turkey	≤ 32 weeks and ≤ 1,500 g who survived to start enteral feeds	29.4 (2.3)	29.2 (2.5)	1,164 (261)	1,131 (284)	271	*S boulardii* (*n =* 135)	Till discharge	5000 × 10^6^ CFU OD	None (*n =* 136)					Mortality, sepsis, NEC	Both HM and F	Duration of stay:43 to 47 days
6.	Dilli et al. (28)	Turkey	<32 weeks and <1,500 g or transferred to NICU within first 7 days and fed enterally	28.8 (1.9)	28.2 (2.2)	1,236 (212)	1,147 (271)	200	*B lactis* (*n =* 100)	Till discharge or max of 8 weeks	5000 × 10^6^ CFU	Placebo (*n =* 100)	3 (3–4)^*^	2 (2–4)^*^	53.0	48.0	Mortality, sepsis, NEC	Both HM and F	Duration of stay: 37 days in probiotic and 50 days in placebo
7.	Dutta et al. (29)	India	27 to 33 weeks; age < 96 h; feeds of at least 15 mL/kg/day	Grp A 30.6 (1.6); grp B 31.1 (1.9) Grp C 30.9 (2.0)	30.8 (1.7)	Grp A 1,286 (265), Grp B 1,286 (265), Grp C 1,413 (296)	1,252 (309)	149	*B longum + L helveticus + L rhamnosus + Sa boulardii* (*n =* 114)	21 days	Grp A: 10^10^ CFU 12 hourly for 21 days; Grp B: 10^10^ CFU 12 hourly for 14 days; Grp C: 10^9^ CFU 12 hourly for 21 days	Placebo (*n =* 35)	72 (48, 92); 72 (54, 90); 93.5 (72, 96)^*^ h	81 (67.5, 96)^*^ h	88.6	97.1	Mortality, sepsis, NEC	Both HM and F	28 days
8.	Fernández-Carrocera et al. (30)	Mexico	<1,500 g	31.2 (26–35.4)$	31 (27–36)$	1,090 (580–1,495)$	1,170 (540–1,492)$	150	*B infantis + L casei + L rhamnosus + L plantarum + L acidophilus + S thermophilus* (*n =* 75)	-	*L acidophilus:* 1 × 10^9^ CFU/g; *L rhamnosus* 440 × 10^6^ CFU/g; *L casei* 1 × 10^9^ CFU/g; *L plantarum* 176 × 10^6^ CFU/g; *B infantis* 27.6 × 10^6^ CFU/g; *S thermophilus* 0.66 × 10^6^ CFU/g	Placebo (*n =* 75)	5 (1–23)^$^	4 (1–15)^$^	21.3	14.7	Mortality, sepsis, NEC	Both HM and F	36–38 days
9.	Gomez Rodriguez et al. (31)	Mexico	<33 weeks; 700 to 1,500 g	Grp A 30.3 (1.83); Grp B 31.3 (2.3)	-	Grp A: 1,175 (21); Grp B: 1,214 (24)		90	Grp A*: L acidophilus* (45); Grp B: *B infantis + L rhamnosus + L casei + L plantarum + L acidophilus + S thermophilus* (*n =* 45)	21 days	1000 × 10^6^ CFU	None (*n =* 45)	5	5	Grp A:60.0; Grp:80.0		Mortality, sepsis, NEC	Both HM and F	26–28 days
10.	Hariharan et al. (32)	India	Birth weight < 1,250 g; gestation < 32 weeks	28.7	29.3	945	972	196	*B bifidum + L acidophilus + Sa boulardii* (*n =* 93)	42 days	2500 × 10^6^ CFU	None (*n =* 103)					Mortality, sepsis, NEC	Both HM and F	NA
11.	Hernandez-Enriquez et al. (33)	Mexico	<34 weeks; <1,500 g					44	*L reuteri* (*n =* 24)	10 days	100 × 10^6^ CFU	Placebo (*n =* 20)					Mortality, sepsis, NEC	Both HM and F	Duration of stay: 39 days for study group vs 50 days for control group
12.	Huang et al. (34)	China	<1,500 g					183	*B adolescentis* (*n =* 95)	Till discharge		Placebo (*n =* 88)					NEC	Both HM and F	NA
13.	Li et al. (35)	China	≤34 weeks and <1,500 g	29.3 (1.3)	30.4 (1.6)	1,176 (164)	1,326 (193)	30	*B longum + B bifidum + L plantarum* (*n =* 16)	36 weeks' PMA	50,000 × 10^6^ CFU	Placebo (*n =* 14)			0	0	Mortality, sepsis	Both HM and F	28 days
14.	Matin et al. (36)	Iran	1,000–1,500 g	31.7 (2.2)	30.8 (2.3)	1,396 (139)	1,362 (143)	52	*L paracasei* (*n =* 26)	28 days	1,500 × 10^6^ CFU	Placebo (*n =* 26)					Mortality, sepsis, NEC	Both HM and F	28 days
15.	Oncel et al. (37)	Turkey	≤32 weeks, <1,500 g	28.2 (2.4)	27.9 (2.5)	1,071 (274)	1,048 (298)	400	*L reuteri* (*n =* 200)	Till discharge or death	100 × 10^6^ CFU	Placebo (*n =* 200)	1 (1–5)^$^	1 (1–5)^$^	17.0	13.0	Mortality, sepsis, NEC	Both HM and F	Duration of stay: 38 days in study group and 49 days in control
16.	Zahed Pasha et al. (38)	Iran	<1,500 g	30.24 (1.57)	30.4 (2.65)	1,245 (176)	1,223 (206)	60	*B infantis + L reuteri + L rhamnosus* (*n =* 30)	Till full feeds achieved	2,000 × 10^6^ CFU	None (*n =* 30)					Mortality, NEC	Both HM and F	Duration of stay: 32 days in study. 41 days in control
17.	Rehman et al. (39)	Pakistan	27–36 weeks; 7 days of age; < 1,500 g	32.5 (2.2)		1,320 (170)		146	Bifidobacterium species + *L acidophilus + S thermophilus + L debrauki*i (*n =* 73)	-		None (*n =* 73)					Mortality, NEC, sepsis	Both HM and F	NA
18.	Rojas et al. (40)	Columbia	<2,000 g; < 48 h old					360	*L reuteri* (*n =* 176)	Till discharge or death	100 × 10^6^CF U	Placebo (*n =* 184)					NEC, Sepsis	Both HM and F	Duration of stay: 32 days in study, 37 days in control
19.	Roy et al. (41)	India	Feeding within 72 h; < 37 weeks; < 2,500 g; < 2 weeks' postnatal age	32 (2)	32.2 (2)	1,192 (341)	1,069 (365)	112	*B longum + B bifidum + B lactis + L acidophilus* (*n =* 56)	Till discharge or 6 weeks	6,000 × 10^6^ CFU	Placebo (*n =* 56)					Mortality, sepsis, NEC	HM only	Duration of stay: 29 days in probiotic and 34 days in control
20.	Saengtawesin et al. (42)	Thailand	≤ 34 weeks; ≤ 1,500 g	31 (1.8)	30.6 (1.8)	1,250 (179)	1,208 (199)	60	*B bifidum + L acidophilus* (*n =* 31)	Till discharge or till 6 weeks	1,000 × 10^6^ CFU each OD	None (*n =* 29)			38.7	37.9	Mortality, sepsis, NEC	Both HM and F	Duration of stay: 60 days in probiotic, 57 days in control.
21.	Samanta et al. (43)	India	<32 weeks; <1,500 g	30.1 (1.6)	30.1 (1.6)	1,172 (143)	1,210 (143)	186	*B longum + B bifidum + B infantis + L acidophilus* (*n =* 91)	Till discharge	2,500 × 10^6^ CFU each OD	None (*n =* 95)	6.0 (1.4)	5.4 (1.3)			Mortality, sepsis, NEC	HM only	Duration of stay: 17 days vs. 24 days
22.	Sari et al. (44)	Turkey	< 33 weeks; < 1,500 g	29.5 (2.4)	29.7 (2.4)	1,231 (262)	1,278 (282)	221	*L sporogenes* (*n =* 110)	Till discharge	350 × 10^6^ CFU each OD	None (*n =* 111)	2	2	23.8	32.8	Mortality, NEC	Both HM and F	Duration of stay: 34 days in study, 30 days in control
23.	Serce et al. (45)	Turkey	≤ 32 weeks; ≤ 1,500g	28.8 (2.2)	28.7 (2.1)	1,126 (232)	1,162 (216)	208	*Saccharomyces boulardii* (*n =* 104)	Till discharge	500 × 10^6^ cells/kg/ dose BD	Placebo (*n =* 104)	2 (1)	1.8 (1.1)	-	-	Mortality, NEC, sepsis	Both HM and F	Duration of stay: 39 days in study, 43 days in control
24.	Shadkam et al. (46)	Iran	28–34 weeks; 1,000–1,800 g	30.9 (1.9)	31 (1.9)	1,396 (234)	1,418 (328)	60	*L reuteri* (*n =* 30)	Till 120 mL/kg/day enteral feeds		Placebo (*n =* 30)			100	100	Mortality, NEC, sepsis	HM only	NA
25.	Shashidhar et al. (47)	India	750–1,499 g	31.2 (2.1)	31 (2.1)	1,256 (185)	1,190 (208)	98	*L acidophilus + L rhamnosus + B longum + S boulardii* (*n =* 49)	Till discharge	1,250 × 10^6^ CFU OD	None (*n =* 49)					Mortality, NEC, sepsis	HM only	Duration of stay: 28 days in study, 31 days in control group
26.	Sowden et al. (48)	South Africa	750–1,500 g; < 37 weeks	29 (0.5)	30 (0.4)	1,174 (226)	1,150 (230)	200	*L acidophilus + B bifidum + B infantis* (*n =* 100)		2,000 × 10^6^ CFU/day	Placebo (*n =* 100)					Mortality, NEC	Both HM and F	NA
27.	Tewari et al. (49)	India	<34 weeks					120	*B clausii* (*n =* 59)	6 wk or discharge or death or LOS	2,400 × 10^6^ CFU	Placebo (*n =* 61)					Mortality, sepsis, NEC	HM only	NA
28.	Van Niekerk et al. (50)	South Africa	<34 weeks and < 1,250 g	28.7		987 (160)		184	*B infantis + L rhamnosus* (*n =* 91)		1,000 × 10^6^ CFU OD	Placebo (*n =* 93)					Mortality, sepsis, NEC	HM only	28 days
29.	Wu et al. (51)	China	28–34 weeks; < 1,500 g; admission within 12 hrs of birth	32.0 (2.6)	31.3 (2.8)	1,240 (180)	1,235 (164)	500	*B longum + L acidophilus + E faecalis* (*n =* 250)	Till TPN was given	10 × 10^6^ CFU/g	None (*n =* 250)			0	0	Sepsis, NEC	F only	14–16 days

P, Probiotic arm; C, Control arm; CFU, colony forming unit; SGA, Small-for-gestational age; IUGR, Intrauterine growth restriction; TPN, total parenteral nutrition; OD, once daily; BD, twice daily. Data represented as mean (SD); ^$^Data represented as median (range); ^*^Data represented as median (IQR); HM Human milk; F, Formula.

### Summary of the included studies

The characteristics of included studies have been summarized in Table 1 (23–51). About 60% of the studies were conducted in India, Turkey, Mexico, and China. The sample size ranged from 30 to 500 in the included studies. The mean gestation of enrolled neonates varied from 28 to 33 weeks, while the mean birth weight varied from 945 to 1,445 g. Almost all the studies initiated the intervention in the first week of life; most only stopped the intervention at discharge. A total of 24 different probiotics—alone or in combination—were evaluated in the included studies (Supplementary Table 6). While 12 studies evaluated a single probiotic species (mainly from the *Lactobacillus* genus), the others used two or more species in combination (mainly from the *Lactobacillus* and *Bifidobacterium* genera). The probiotic dosage varied from 10 × 10^6^ to 50,000 × 10^6^ colony-forming units (CFU). Among the studies that reported the type of milk received by the neonates, the exclusive breast milk feeding proportion varied from 13 to 100%.

### Risk of bias assessment

The risk of bias assessment of the included studies is shown in Supplementary Figure 1A. More than half of the studies had an unclear or high risk of bias arising from the randomization process; about 30% of the included studies had a high risk of bias due to deviations from the intended intervention (Supplementary Figures 1A, B). Only 11 studies (38%) had a low risk of bias (Supplementary Figure 1B).

#### Outcomes

##### Mortality

**Figure 2:**
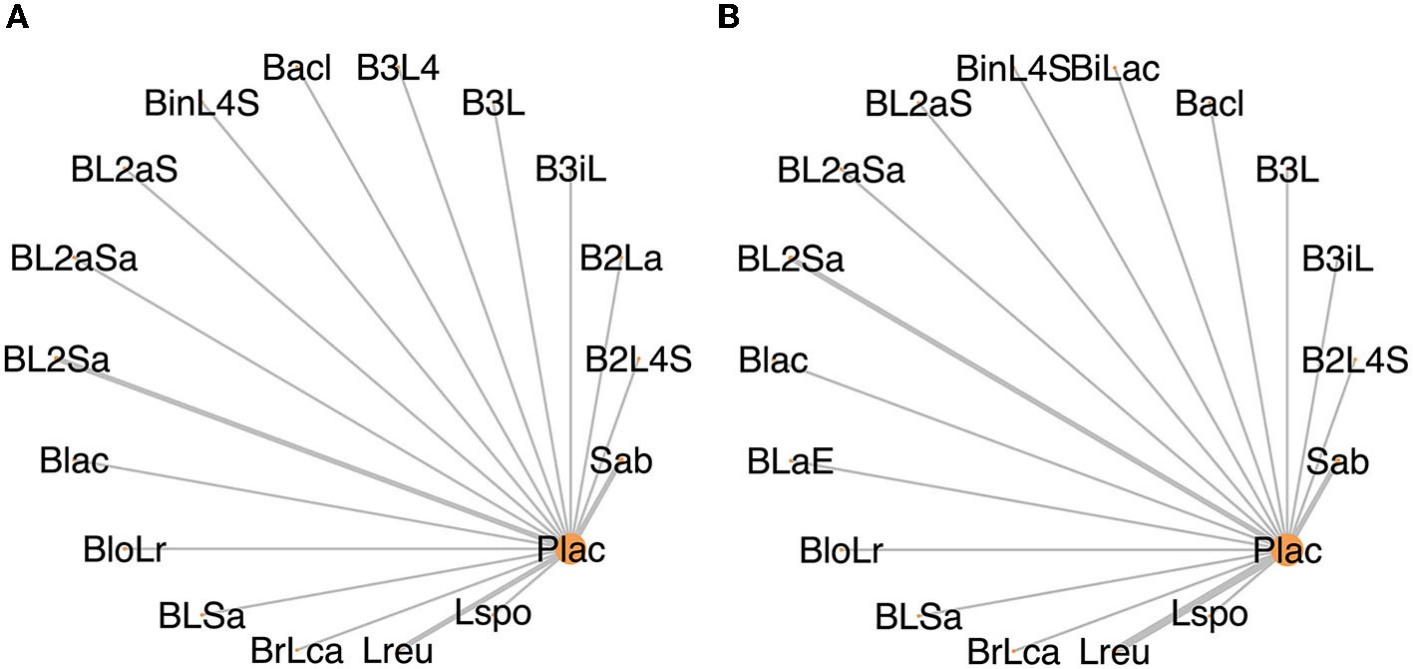
**(A)** Network plot depicting the studies included for mortality. **(B)** Network plot depicting the studies included for sepsis. The nodes represent the interventions evaluated in the network. The size of the nodes is proportional to the number of patients assigned to the intervention, while the thickness of the lines connecting the nodes is proportional to the number of pairwise trials that evaluated the interventions (shown as numbers along the lines). Refer to Figure 3 for the expansion of the abbreviations.

A total of 26 studies involving 3,863 neonates provided information on all-cause mortality. Six studies had zero events in at least one of the treatment arms and were dropped in the final analysis. The remaining 20 studies had enrolled 3,527 neonates, of whom 267 (7.6%) died. Figure 2A illustrates the network plot—each intervention arm has been compared with the standard reference arm (placebo) but not among themselves, thereby precluding the formation of any closed loop in the network. Based on the visual examination of the leverage plots and comparison of the DIC values of the fixed- and random-effects model (Supplementary Figures 2A, B), we chose the fixed-effect model for estimating the effect size and credible intervals. The trace and density plots demonstrated good convergence of the model (Supplementary Figure 3A).

**Figure 3:**
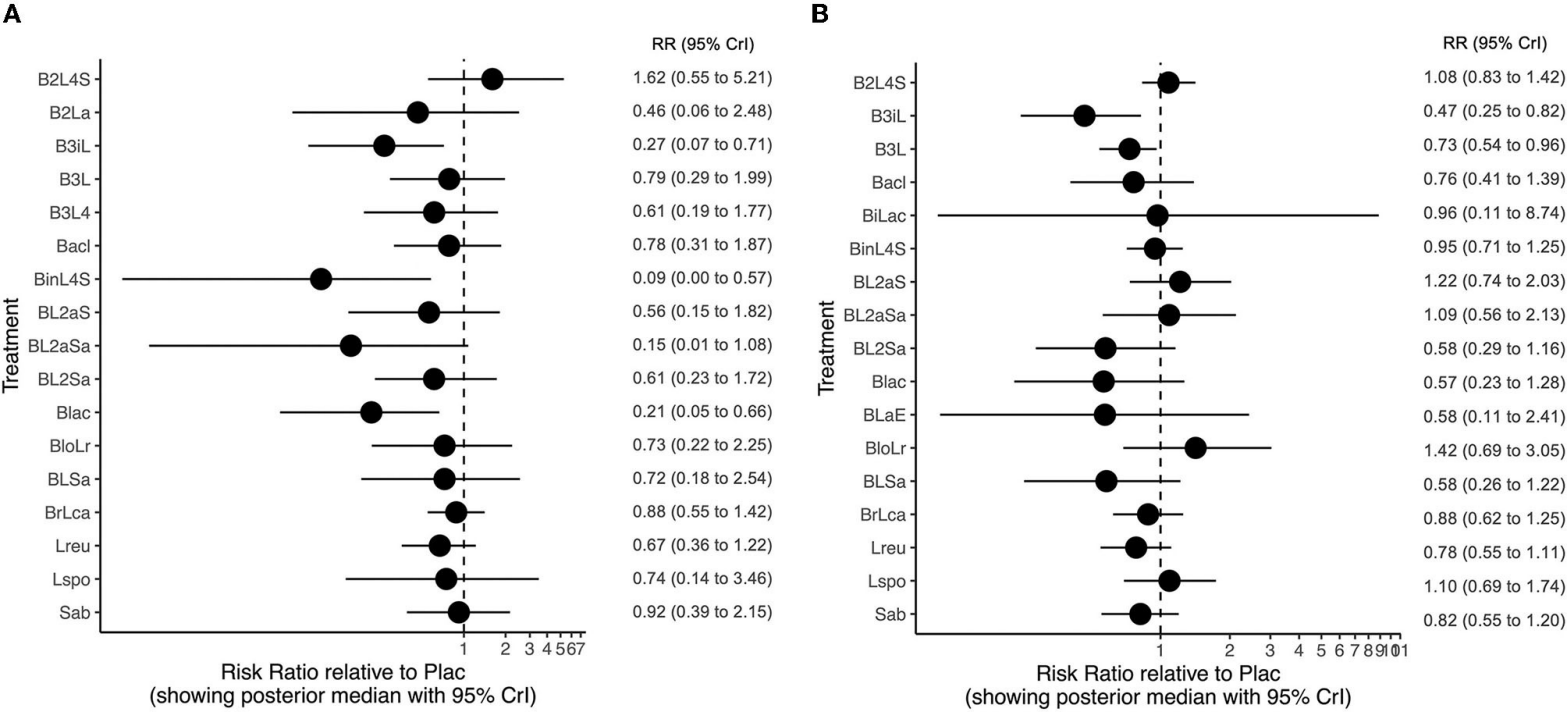
**(A)** Forest plots depicting the relative risks of different probiotic species compared with placebo for mortality. **(B)** Forest plots depicting the relative risks of different probiotic species compared with placebo for sepsis. Sab, *Saccharomyces boulardii; L spo, Lactobacillus sporogenes; L reu, L reuteri; BrLca, B breve*+*L casei; BLSa, B bifidum*+*L acidophilus*+*Sa boulardii; BloLr, B longum*+*L rhamnosus; BLaE; L acidophilus*+ *b infantis*+ *bacillus cereus*+ *E fecalis; Blac, B lactis; BL2Sa, B longum*+*L helveticus*+*L rhamnosus*+*Sa boulardii; BL2aSa, B longum*+*L acidophilus*+*L rhamnosus*+*Sa boulardii; BL2aS, B spp*+*L acidophilus*+*S thermophilus*+*L delbrueckii; BinL4S, B infantis*+*L rhamnosus*+*L casei*+*L plantarum*+*L acidophilus*+*S thermophilus;BiLac, B bifidum*+*L acidophilus; Bacl, Ba clausii; B3L4, B bifidum*+ *B longum*+*B infantis*+*L rhamnosus*+*L paracasei*+*L casei*+*L acidophilus*+*L lactis; B3L, B longum*+*B bifidum*+*B lactis*+*L acidophilus; B3iL, B longum*+*B bifidum*+*B infantis*+*L acidophilus; B2L4S, B longum*+ *B breve*+*L acidophilus*+*L rhamnosus*+*L bulgaricus*+*L casei*+*S thermophiles*.

**Figure 4:**
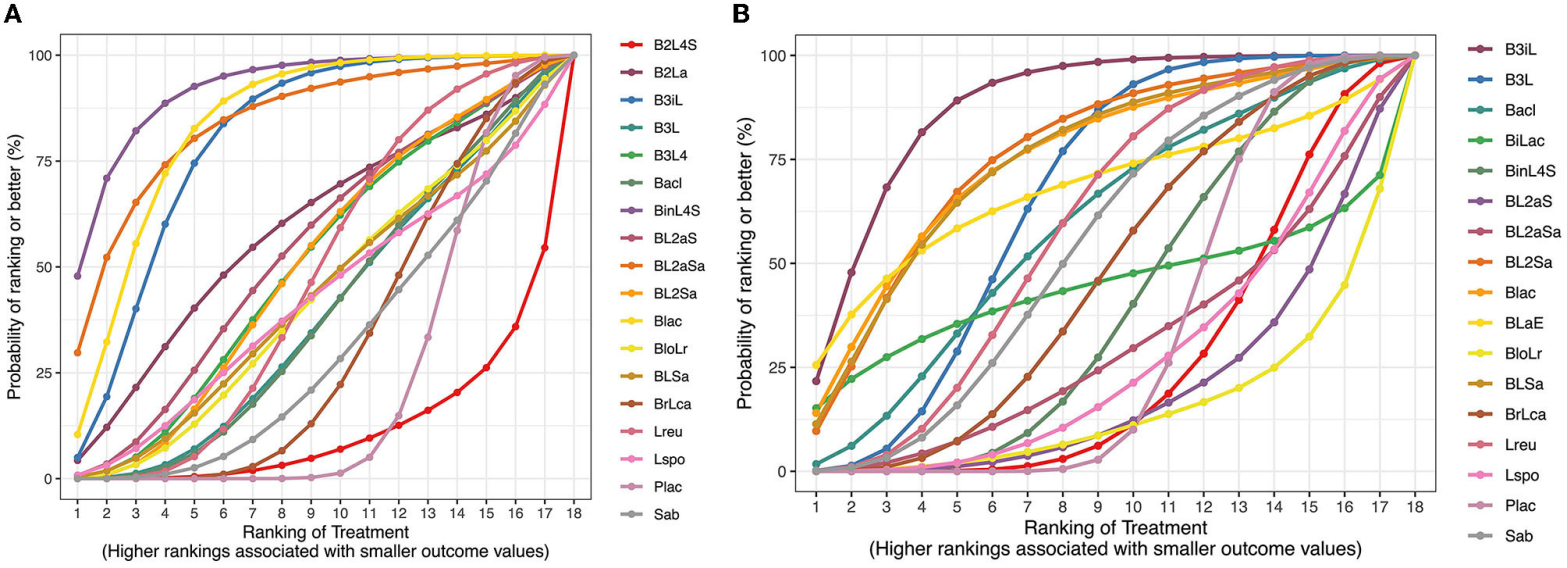
**(A)** SUCRA plot of the network meta-analysis for mortality. **(B)** SUCRA plot of the network meta-analysis for sepsis. Refer to Figure 3 for the expansion of the abbreviations.

When compared with the placebo, three probiotic arms, namely, *B lactis* alone (“B lac”; RR 0.21; 95% CrI 0.05 to 0.66; low certainty of evidence); the combination of *B longum, B bifidum, B infantis, and L acidophilus* (“B3iL”; RR 0.26, 95% CrI 0.07 to 0.72; very low certainty of evidence); and that of *B infantis, L rhamnosus, L casei, L plantarum, L acidophilus, and S thermophilus* (“BinL4S”; RR 0.09, 95% CrI 0.003 to 0.576; low certainty of evidence) may reduce the risk of mortality (Figure 3A, Supplementary Table 8). SUCRA values ranked “BinL4S” (SUCRA 0.92), “BL2aSa” (combination of *B longum, L acidophilus, L rhamnosus*, and *Sa boulardii*; SUCRA 0.84;), “B lac” (SUCRA 0.84), and “B3iL” (SUCRA 0.80; Figure 4A) as the most beneficial interventions. The league plot (Supplementary Figure 4A) of the network estimates confirmed the findings of the SUCRA plot. The split between direct and indirect evidence could not be checked because of the absence of closed loops in the network. However, the deviance contribution plot (Supplementary Figure 5A) showed most points near or above the line of equality, suggesting a lack of critical inconsistency.

#### Sepsis

Twenty-four studies involving 4,314 neonates reported on the incidence of sepsis. All but two studies (23, 42) had documented culture-positive sepsis. Three studies had zero events in at least one arm and were dropped in the final analysis. The remaining 21 studies enrolled 4,112 neonates, of whom 825 (20.1%) were diagnosed with sepsis. Figure 2B illustrates the network plot—each probiotic arm has been compared with the standard reference arm (placebo) but not among themselves. The fixed-effect model was used to estimate the effect size and credible intervals after examining the leverage plots and comparing the DIC values of the fixed- and random-effects models (Supplementary Figures 2C, D).

When compared with the placebo, two probiotic arms, namely, the combinations of *B longum, B bifidum, B infantis*, and *L acidophilus* (“B3iL”; RR 0.47, 95% CrI 0.25 to 0.83; very low certainty of evidence) and *B longum, B bifidum, B lactis*, and *L acidophilus* (“B3L”; RR 0.73; 95% CrI 0.54 to 0.96; low certainty of evidence) may result in a reduction in the incidence of sepsis (Figure 3B, Supplementary Table 9). SUCRA values ranked “B3iL” (SUCRA 0.88), “BL2Sa” (combination of *B longum, L helveticus, L rhamnosus*, and *Sa boulardii*; SUCRA 0.76), and “B lac” (*B lactis*; SUCRA 0.75; Figure 4B) as the most beneficial interventions. The league plot (Supplementary Figure 4B) of the network estimates confirmed the findings of the SUCRA plot. The split between direct and indirect evidence could not be checked. The deviance contribution plot (Supplementary Figure 5B) did not suggest any critical inconsistency.

#### Necrotizing enterocolitis

A total of 28 studies involving 4,876 neonates reported the incidence of NEC. All but two studies provided the risk of NEC stage 2 or more; two studies Rehman et al. (39) and Roy et al. (41)—did not mention the stage of NEC. Nine studies with zero events in at least one study arm were dropped from the analysis. The remaining 19 studies enrolled 3,527 neonates, of whom 65 (1.8%) had NEC. Supplementary Figure 6 illustrates the network plot wherein each probiotic arm has been compared with the standard reference arm (placebo) but not among themselves. The fixed-effect model was used to estimate the effect size and credible intervals after examining the leverage plots and comparing the DIC values of the fixed- and random-effects models (Supplementary Figures 2E, F). The trace and density plots demonstrated good convergence of the model (Supplementary Figure 3B).

Six probiotic regimens, namely *B lactis* (“B lac”; RR 0.09, 95% CrI 0.01–0.32; low certainty of evidence), *L reuteri* (“Lreu”; RR 0.40, 95% CrI 0.23–0.68; low certainty of evidence), and the combinations *of B bifidum, B longum, B infantis, L rhamnosus, L paracasei, L casei, L acidophilus*, and *L lactis* (“B3L4”; RR 0.10, 95% CrI 0.003–0.66; very low certainty of evidence), *B longum, L acidophilus*, and *E fecalis* (“BLaE”; RR 0.13, 95% CrI 0.01–0.51; low certainty of evidence), *Bifidobacterium spp., L acidophilus, S thermophilus*, and *L delbrueckii* (“BL2aS”; RR 0.19, 95% CrI 0.02–0.78; low certainty of evidence), *B longum, B bifidum, B infantis*, and *L acidophilus* (“B3iL”; RR 0.31; 95% CrI 0.10–0.78; very low certainty of evidence) may result in a reduction in the incidence of NEC (Supplementary Figure 7). SUCRA values ranked “B lac” (SUCRA 0.88), “B3L4” (SUCRA 0.82), and “BLaE” (SUCRA 0.80; Supplementary Figure 8) as the most beneficial interventions. The league plot (Supplementary Figure 4C) of the network estimates confirmed the findings of the SUCRA plot.

### Safety outcomes and subgroup analyses

Fifteen studies that evaluated the risk of probiotic-related sepsis as a safety outcome found no incidence of culture-positive sepsis attributable to the probiotic administered in any of the arms (27–31, 36, 37, 40, 42–45, 47, 49, 50).

On subgroup analyses by the type of milk received by the enrolled neonates, two probiotic arms, namely, *B lactis* alone (“B lac”; RR 0.21; 95% CrI 0.05 to 0.66; low certainty of evidence) and that of *B infantis, L rhamnosus, L casei, L plantarum, L acidophilus, and S thermophilus* (“BinL4S”; RR 0.09, 95% CrI 0.003 to 0.58) possibly reduced the risk of mortality (Supplementary Figure 9A) among those receiving either breastmilk or formula feeds. In contrast, the combination of *B longum, B bifidum, B infantis*, and *L acidophilus* (“B3iL”; RR 0.26; 95% CrI 0.07 to 0.71) may reduce the mortality risk in exclusively breastmilk-fed neonates (Supplementary Figure 9B). None of the probiotics reduced the incidence of sepsis among neonates receiving breastmilk or formula feeds (Supplementary Figure 10A); however, among those receiving only breastmilk, two probiotic combinations, namely, *B longum, B bifidum, B infantis, and L acidophilus* (“B3iL”; RR 0.47, 95% CrI 0.25 to 0.82) and *B longum, B bifidum, B lactis*, and *L acidophilus* (“B3L”; RR 0.73, 95% CrI 0.54 to 0.96) possibly reduced the risk of sepsis (Supplementary Figure 10B).

## Discussion

The results of the current review suggest that the combination of *B longum, B bifidum, B infantis*, and *L acidophilus* (“B3iL”) may reduce the risks of mortality, sepsis, and NEC in preterm very low birth weight neonates when compared to placebo, but the evidence was very uncertain; the single probiotic species—*B lactis*—may reduce the incidence of mortality and NEC, with the certainty of the evidence being low. The individual study that compared “B3iL” with the placebo did not show a significant reduction in either mortality or sepsis, possibly because of the small sample size and low event rate. The network meta-analysis probably improved the precision of the result. In addition to “B3iL” and *B lactis*, four more probiotic combinations may reduce the incidence of NEC. The certainty of the evidence was, however, low.

The review findings are concordant with that of the previous network meta-analysis by van den Akker et al., which reported that the probiotic combination of *B longum, B bifidum, B infantis, and L acidophilus* (“B3iL”) reduced the incidence of mortality and late-onset sepsis while the single probiotic species *B lactis* reduced the risk of NEC (52). However, the other probiotic combinations found to reduce the incidence of NEC—*L rhamnosus* GG or the combination of *B infantis* and *L acidophilus*—were not shown to be beneficial in the current study. The other network meta-analysis by Morgan et al. found the combinations of *Lactobacillus spp*. and *Bifidobacterium spp*. (mainly, *L rhamnosus* GG and *B longum* subsp *infantis; Lactobacillus casei* and *B breve)* to be among the most effective regimens in reducing the incidence of all-cause mortality and NEC (53); it did not find any probiotic species to be beneficial in reducing the risk of culture-proven sepsis.

The discrepancy in results wasn't unexpected given the focus of the current review on *only* the studies from low- and middle-income countries (cf. previous reviews that included all studies irrespective of the settings). The other reviews did not provide the subgroup analyses of the studies from LMICs, which precludes direct head-to-head comparison with the current review. Notwithstanding these issues, the discordant results could be because of the differences in the maternal genital tract and gut microbiome, exposure to broad-spectrum antibiotics in the antenatal and postnatal periods, resistant environmental flora in the delivery areas and neonatal units, mode of delivery, rates of intrauterine growth restriction, exclusive breastfeeding rates, and use of fortifiers. The predominance of Gram-negative pathogens among neonates with early-onset sepsis in LMICs, as opposed to that by group B streptococci in neonates from high-income countries (HIC), could indicate a qualitatively different vaginal flora and gut microbiome among mothers from the two settings (54, 55). The mode of delivery and breastfeeding rates have been demonstrated to influence the gut microbiome of preterm neonates (56, 57). Together, these factors underscore the potential problems in extrapolating the results of probiotic studies from HICs to LMIC settings.

The clinical practice and public health implications of the current network meta-analysis's results are unknown. There is uncertain evidence on the effects of the probiotic combination of *B longum, B bifidum, B infantis, and L acidophilus*, the only regimen found to be beneficial in reducing the incidence of all three outcomes. Only one study involving 186 neonates evaluated its efficacy. The ESPGHAN Working Group for Probiotics and Prebiotics (52), which chose a minimum number of 247 infants per group to be studied before making recommendations, did not consider the said probiotic combination because of the small numbers enrolled (52). Moreover, the ESPGHAN group has cautioned against using probiotic strains that produce D-lactate because of the lack of safety data in preterm neonates. *L acidophilus* is a partially D-lactate-producing strain. On the other hand, the Working Group has conditionally recommended using *L rhamnosus GG* to reduce the risk of NEC in preterm neonates. None of the studies included in the review have evaluated its efficacy in neonates from LMICs. The recent WHO guidelines for the care of preterm and low-birth-weight infants also did not make any recommendations on the type, formulation, dose, timing, or duration of probiotics due to a lack of sufficient evidence (58).

Our review suggested that the single probiotic species *B lactis* may reduce the risk of mortality and NEC. Unfortunately, it is not commonly available in India and possibly other LMICs (Supplementary Table 9). There is an urgent need to examine (a) the efficacy of *B lactis, L rhamnosus* GG, or their combinations to identify the optimal probiotic species for use in LMIC settings and (b) the safety of probiotic combinations containing *L reuteri* or *L acidophilus* that produce D-lactate in preterm neonates from LMICs.

The current review is arguably the first species-specific network meta-analysis involving studies from LMICs. The overarching goal was to identify the optimal probiotic species that provides the maximal benefits in reducing the risks of mortality and sepsis in preterm neonates from these settings, which would not have been possible with the traditional pair-wise meta-analyses comparing any probiotics with placebo. However, none of the included studies compared one probiotic species with another, thus precluding obtaining direct and network estimates for each comparison and checking the consistency between the direct and indirect estimates. Therefore, we did not use or interpret the estimates from indirect comparisons among the probiotic regimens. The other critical limitation of the review was that all the trials were relatively small, and only one study each had evaluated almost all probiotic regimens. Finally, we had to drop studies with zero events from the analysis to avoid convergence issues and prevent getting spurious estimates.

To conclude, no firm conclusions can be made on the optimal probiotic species to be used in preterm very low birth weight neonates in LMICs because of the lack of direct comparisons between different probiotics and the low to very low certainty of the evidence for the efficacy of the two probiotics found to reduce mortality and necrotizing enterocolitis. Future studies should evaluate the efficacy of *B lactis, L rhamnosus* GG, or their combinations to guide clinical practice and policymaking in these settings.

## Data availability statement

The original contributions presented in the study are included in the article/Supplementary material, further inquiries can be directed to the corresponding author.

## Author contributions

MS: had full access to all of the data in the study and takes responsibility for the integrity of the data and the accuracy of the data analysis, administrative, technical, or material support, and supervision. DT, AS, and MS: concept, design, acquisition, analysis, and interpretation of data. DT and AS: drafting of the manuscript and statistical analysis. All authors: critical revision of the manuscript for important intellectual content.

## Supplementary material

Supplementary Figure 1 Risk of bias of the included studies: **(A)** overall plot and **(B)** traffic light plot.

Supplementary Figure 2 Fixed effect model of the network meta-analysis for **(A)** Mortality, **(C)** Sepsis, and **(E)** NEC and random effect model for **(B)** Mortality, **(D)** Sepsis, and **(F)** NEC.

Supplementary Figure 3 Trace and density plot for **(A)** Mortality and **(B)** Sepsis.

Supplementary Figure 4 League table heatmap of the network meta-analysis for **(A)** Mortality, **(B)** Sepsis, and **(C)** NEC. The decoding of the probiotics has been given in Supplementary Table 6. The values in each cell represent the relative risk (and 95% credible intervals) of the treatment on the top, compared to the treatment on the left. A double asterisk indicates statistical significance.

Supplementary Figure 5 Deviance contribution plots of the network meta-analysis for **(A)** Mortality, **(B)** Sepsis, and **(C)** NEC.

Supplementary Figure 6 Network plot depicting the studies included for the secondary outcome- NEC.

Supplementary Figure 7 Forest plot depicting the relative risks of different probiotic species compared with placebo for the secondary outcome- NEC.

Supplementary Figure 8 SUCRA plot of the network meta-analysis for the secondary outcome- NEC. The decoding of the probiotics has been given in Supplementary Table 6.

Supplementary Figure 9 Forest plot depicting the relative risks of different probiotic species compared with placebo for mortality among patients fed **(A)** Both human milk and formula **(B)** Human milk only

Supplementary Figure 10 Forest plot depicting the relative risks of different probiotic species compared with placebo for sepsis among patients fed **(A)** Both human milk and formula **(B)** Human milk only.

Supplementary Table 1 Deviations in the protocol.

Supplementary Table 2 Search strategy from electronic databases.

Supplementary Table 3 Excluded studies after full-text screening.

Supplementary Table 4 Studies awaiting classification.

Supplementary Table 5 Characteristics of ongoing studies.

Supplementary Table 6 Coding of probiotics used alone or in combination used in the study.

Supplementary Table 7 Summary of confidence rating of various probiotic interventions compared to placebo for mortality.

Supplementary Table 8 Summary of confidence rating of various probiotic interventions compared to placebo for sepsis.

Supplementary Table 9 Commonly used probiotic preparations for neonatal use in India.

Supplementary Table 10 PRISMA-NMA checklist.
