# Supplementary figures and images for: Probiotics for the prevention of mortality and sepsis in preterm very low birth weight neonates from low- and middle-income countries: a Bayesian network meta-analysis
Source: Front Nutr. 2023 Jun 14;10:1133293. doi: 10.3389/fnut.2023.1133293 (PMC10300419; doi:10.3389/fnut.2023.1133293)

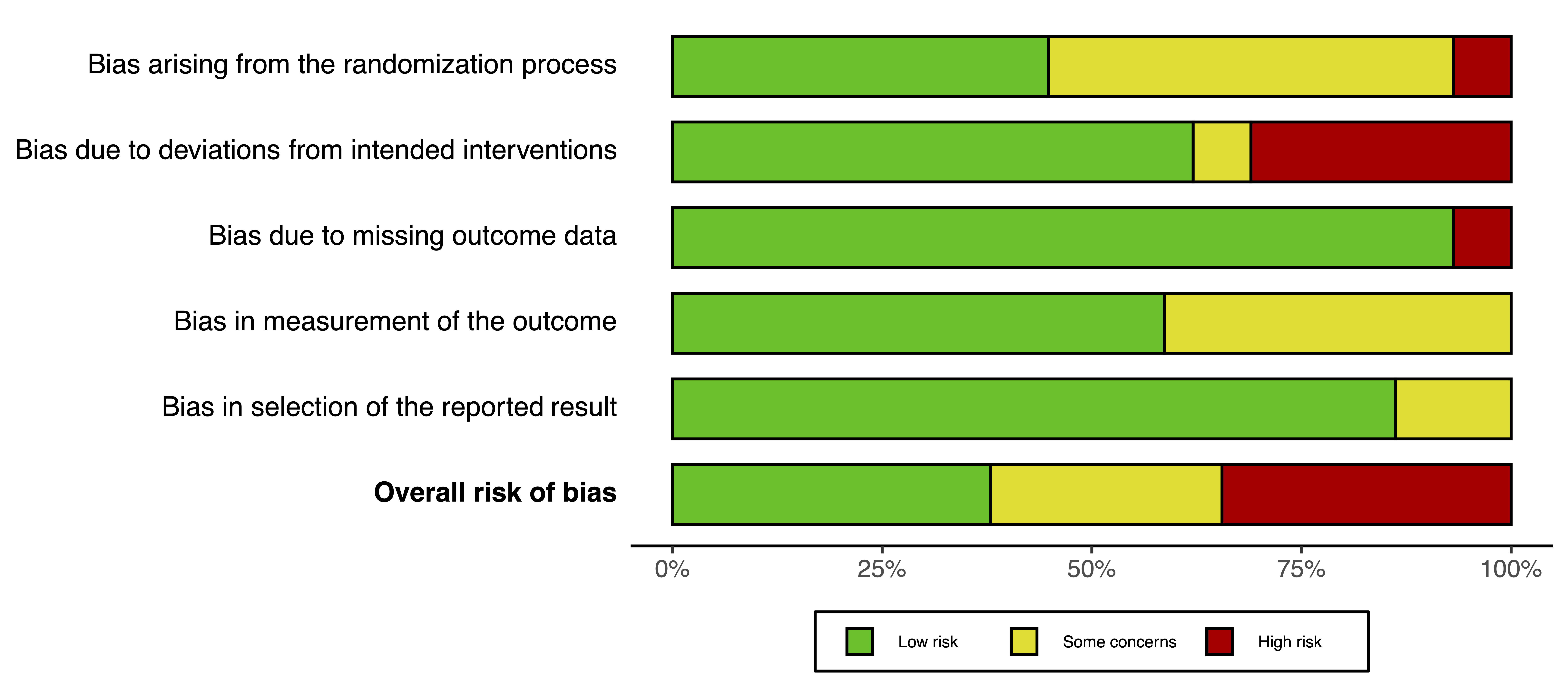

Supplement: Supplementary Figure 1 — Risk of bias of the included studies: (A) overall plot and (B) traffic light plot. [file Data_Sheet_2.ZIP › Suppl figures/Supplementary Figure 1 (A).jpg]

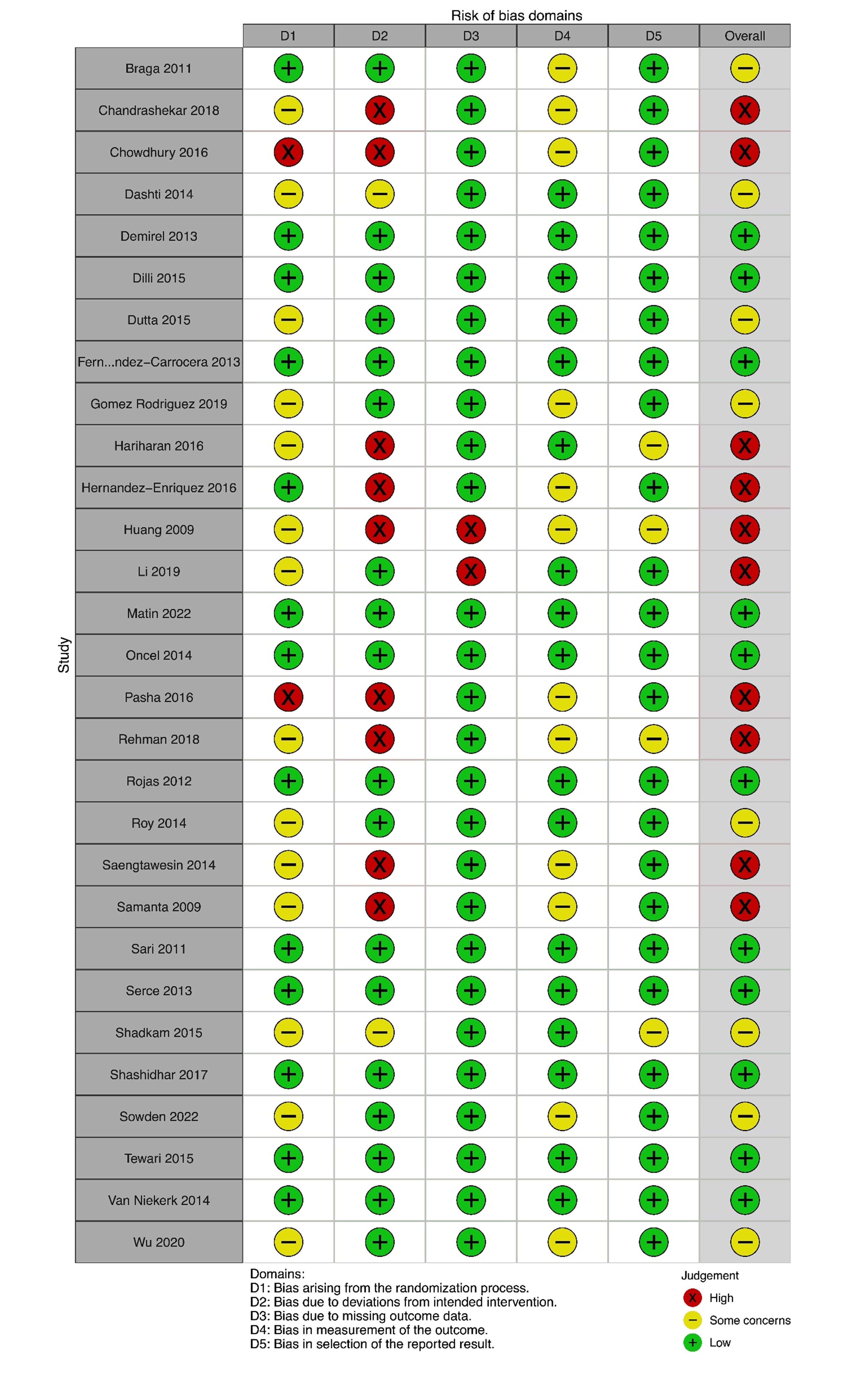

Supplement: Supplementary Figure 1 — Risk of bias of the included studies: (A) overall plot and (B) traffic light plot. [file Data_Sheet_2.ZIP › Suppl figures/Supplementary Figure 1 (B).jpg]

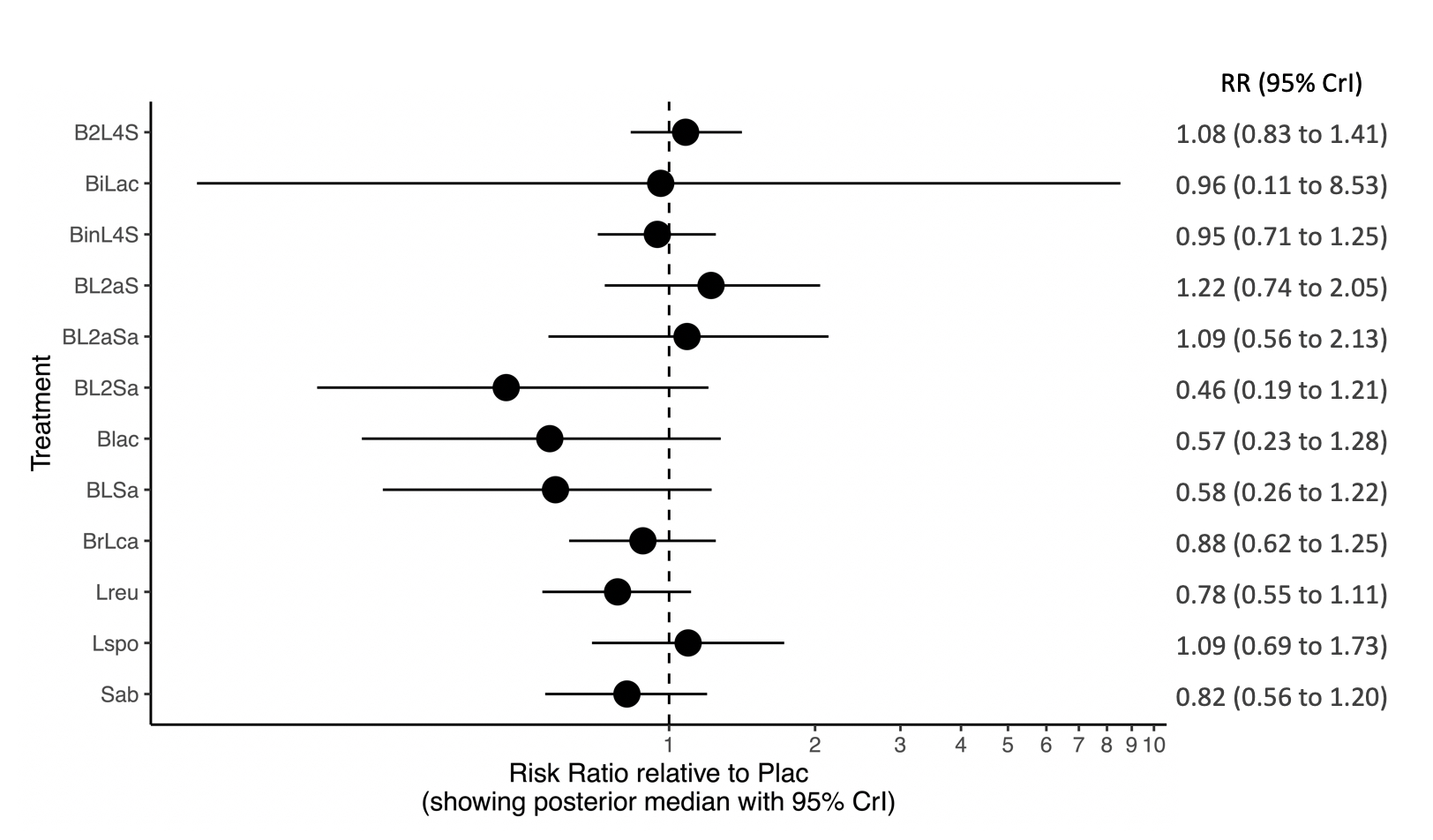

Supplement: Supplementary Figure 1 — Risk of bias of the included studies: (A) overall plot and (B) traffic light plot. [file Data_Sheet_2.ZIP › Suppl figures/Supplementary Figure 10 (A).jpg]

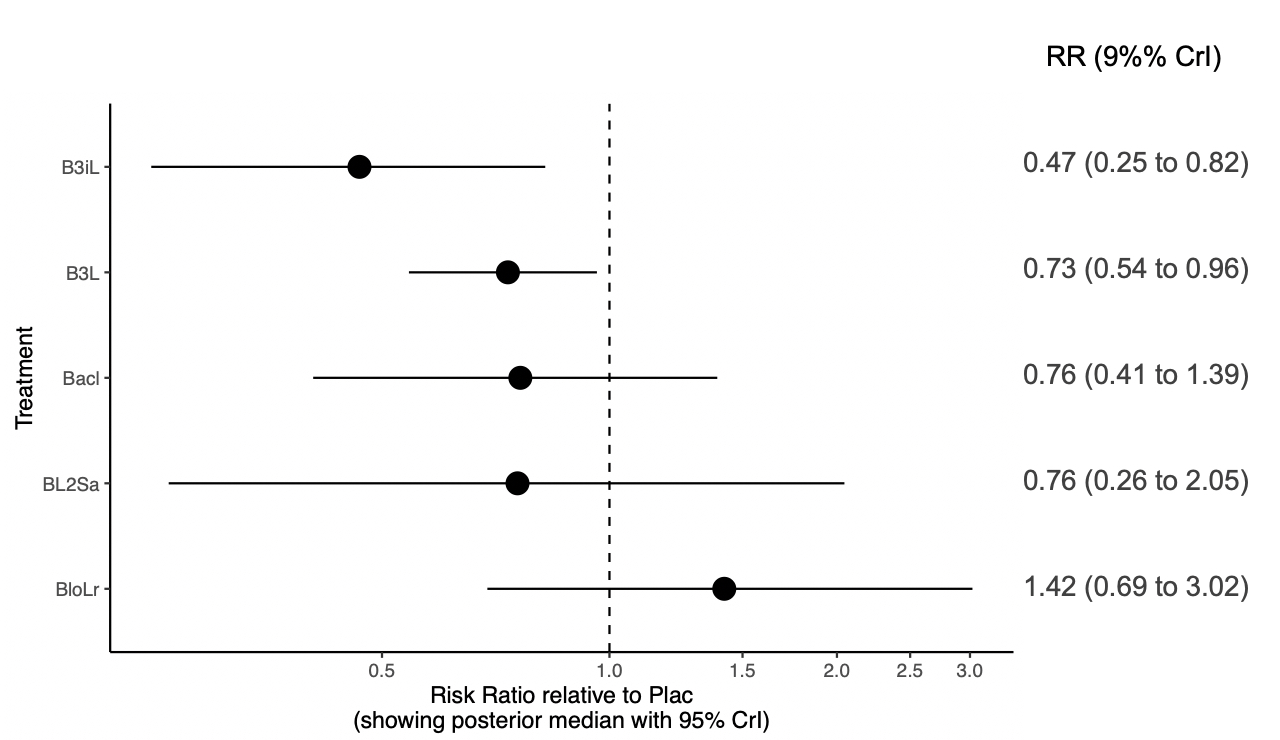

Supplement: Supplementary Figure 1 — Risk of bias of the included studies: (A) overall plot and (B) traffic light plot. [file Data_Sheet_2.ZIP › Suppl figures/Supplementary figure 10 (B).jpg]

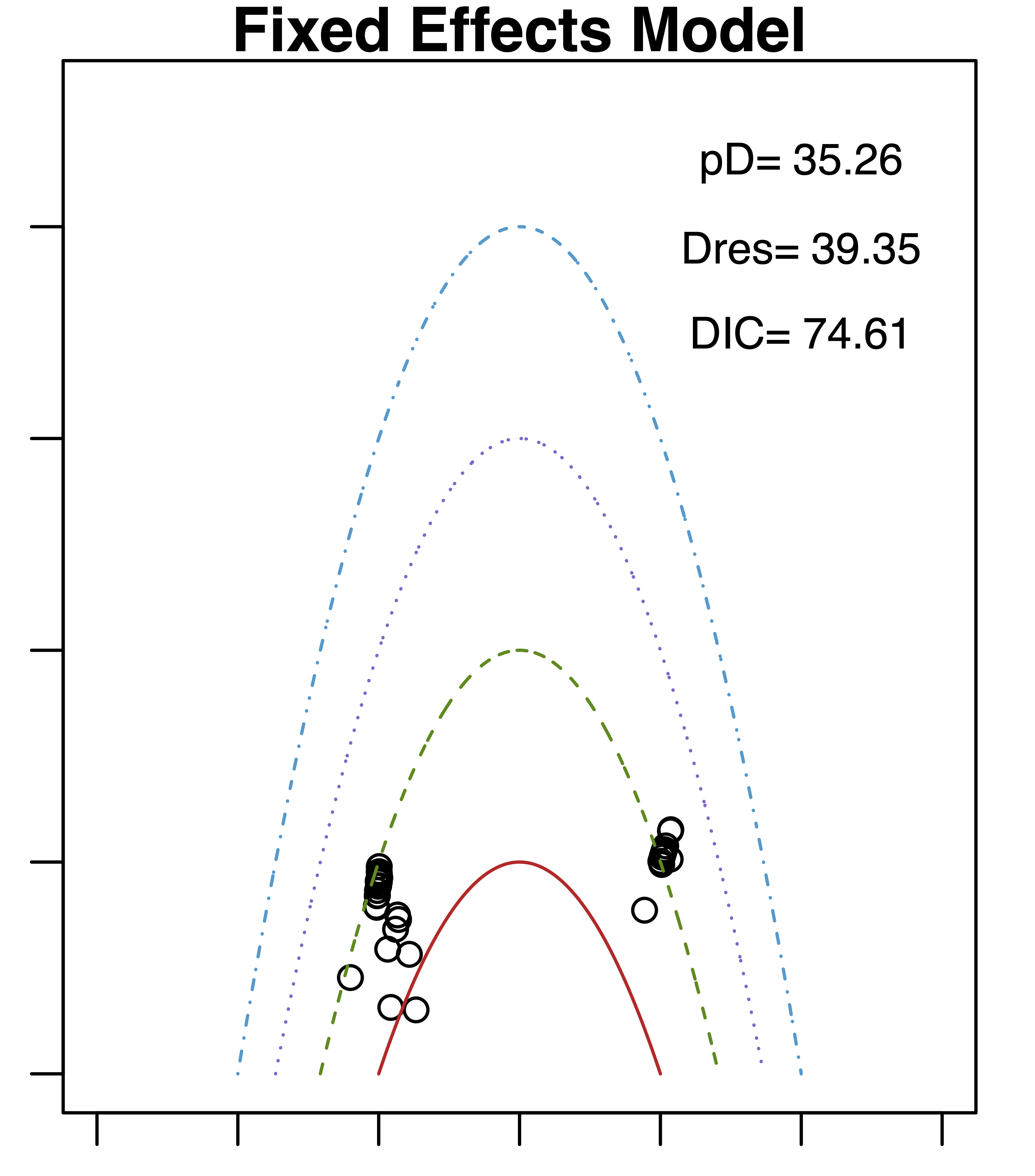

Supplement: Supplementary Figure 1 — Risk of bias of the included studies: (A) overall plot and (B) traffic light plot. [file Data_Sheet_2.ZIP › Suppl figures/Supplementary figure 2 (A).jpg]

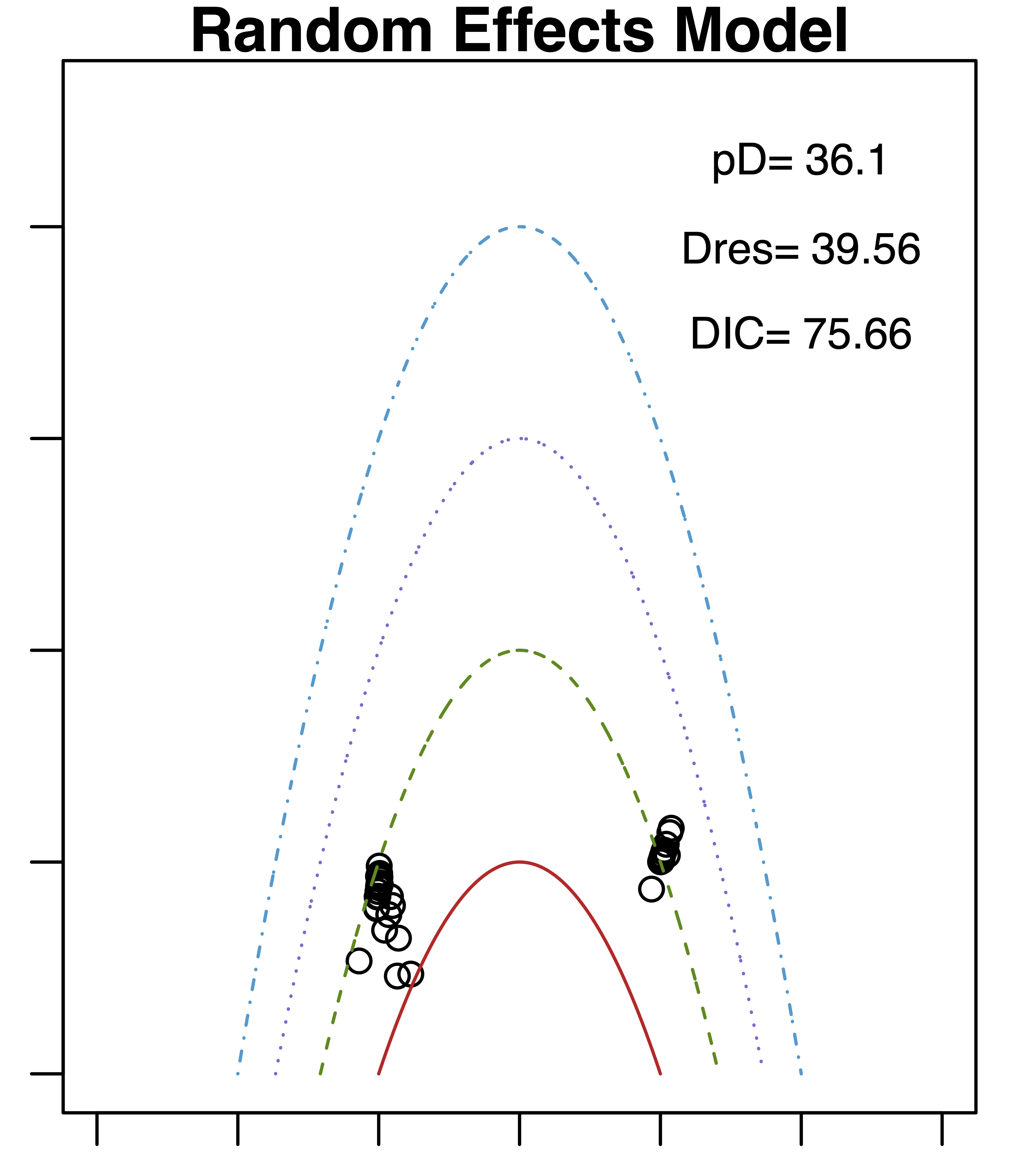

Supplement: Supplementary Figure 1 — Risk of bias of the included studies: (A) overall plot and (B) traffic light plot. [file Data_Sheet_2.ZIP › Suppl figures/Supplementary Figure 2 (B).jpg]

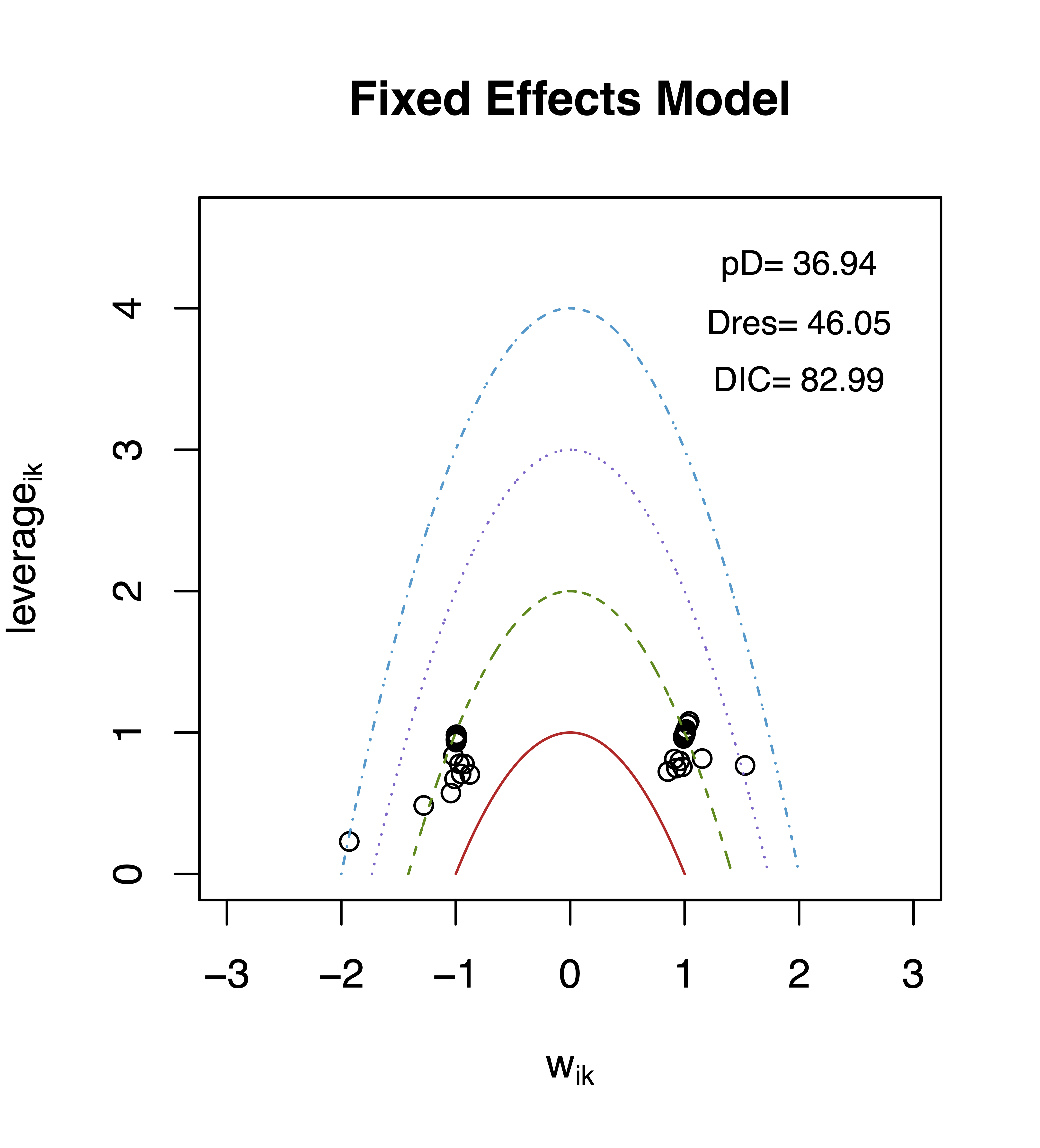

Supplement: Supplementary Figure 1 — Risk of bias of the included studies: (A) overall plot and (B) traffic light plot. [file Data_Sheet_2.ZIP › Suppl figures/Supplementary figure 2 (C).jpg]

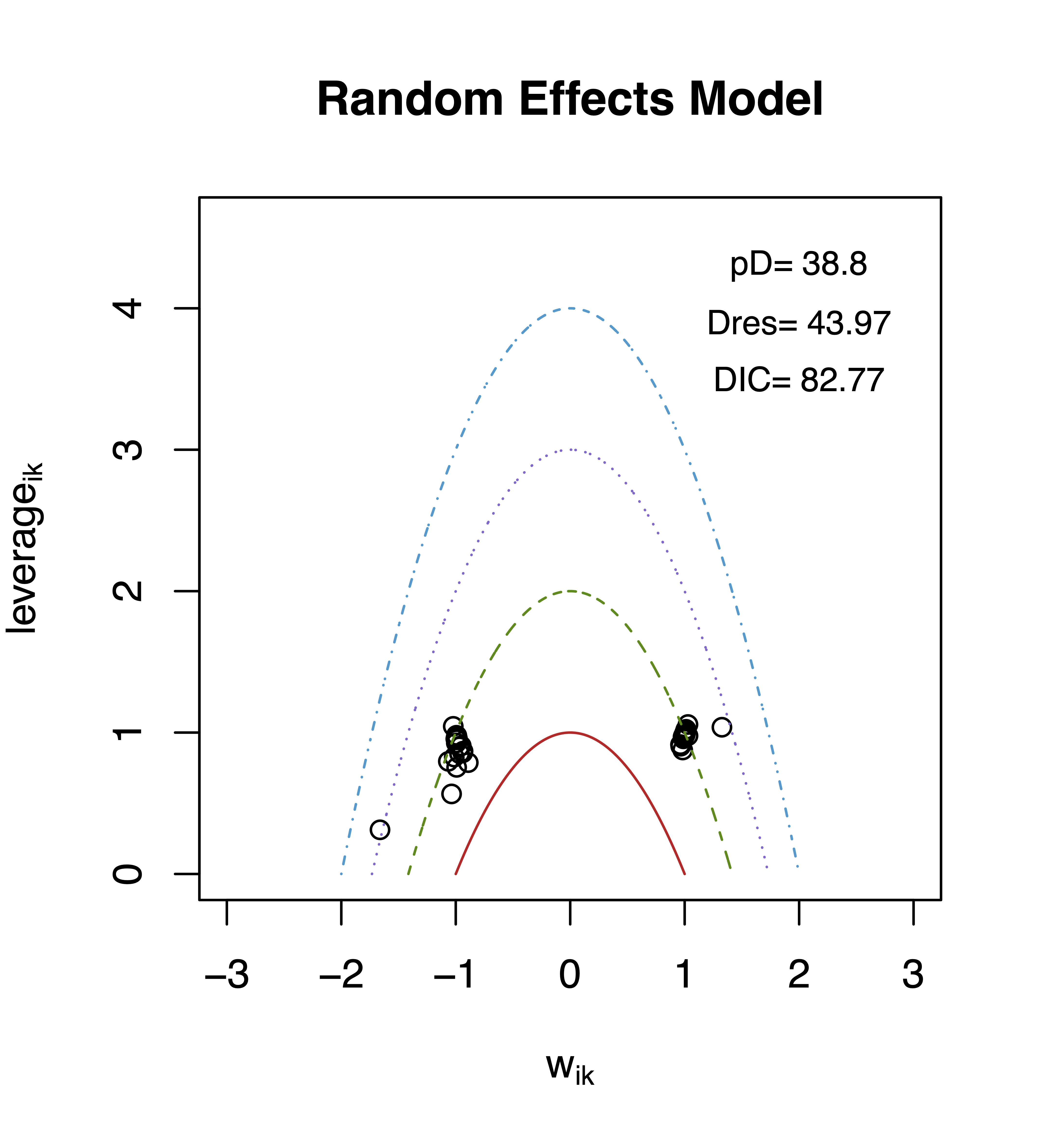

Supplement: Supplementary Figure 1 — Risk of bias of the included studies: (A) overall plot and (B) traffic light plot. [file Data_Sheet_2.ZIP › Suppl figures/Supplementary figure 2 (D).jpg]

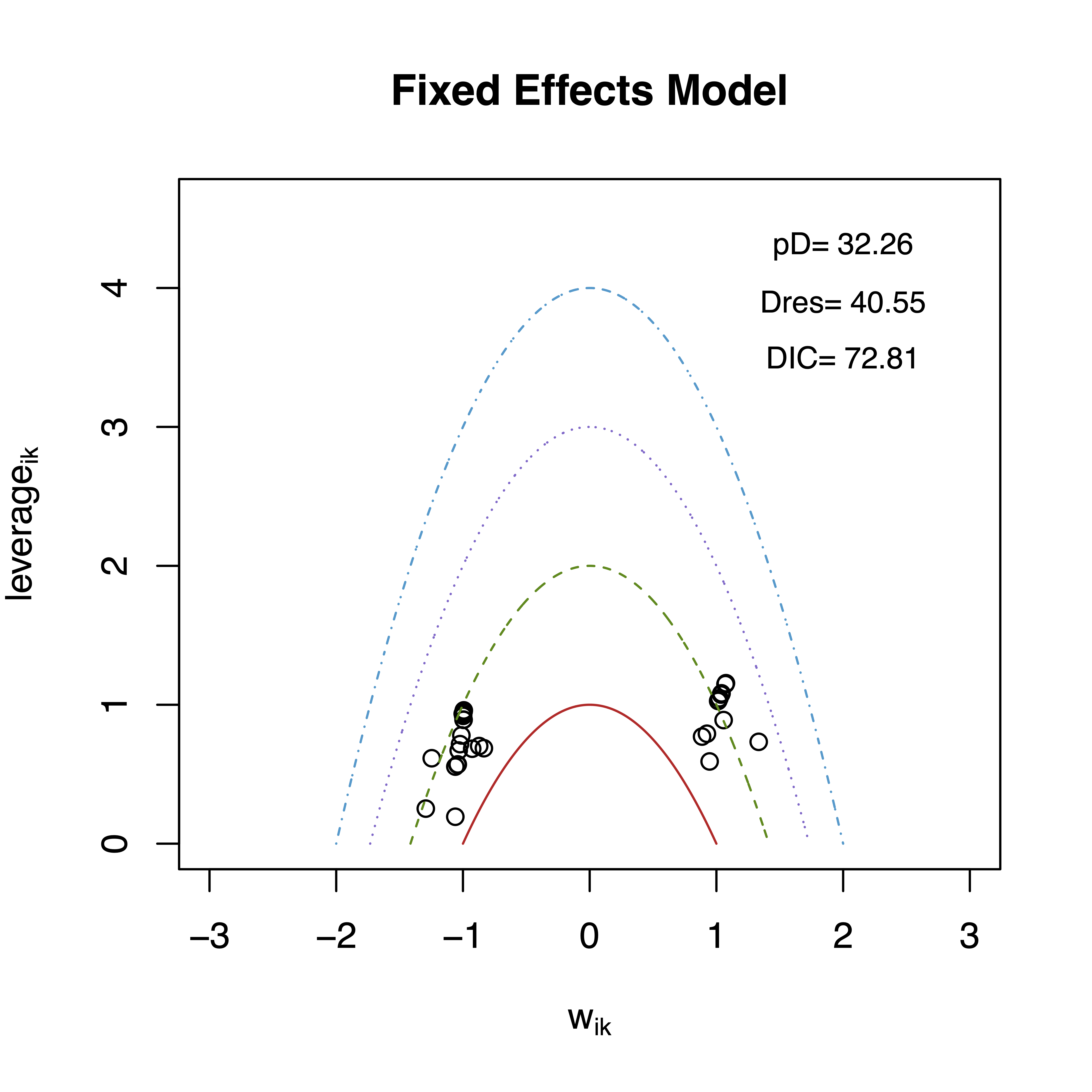

Supplement: Supplementary Figure 1 — Risk of bias of the included studies: (A) overall plot and (B) traffic light plot. [file Data_Sheet_2.ZIP › Suppl figures/Supplementary figure 2 (E).jpg]

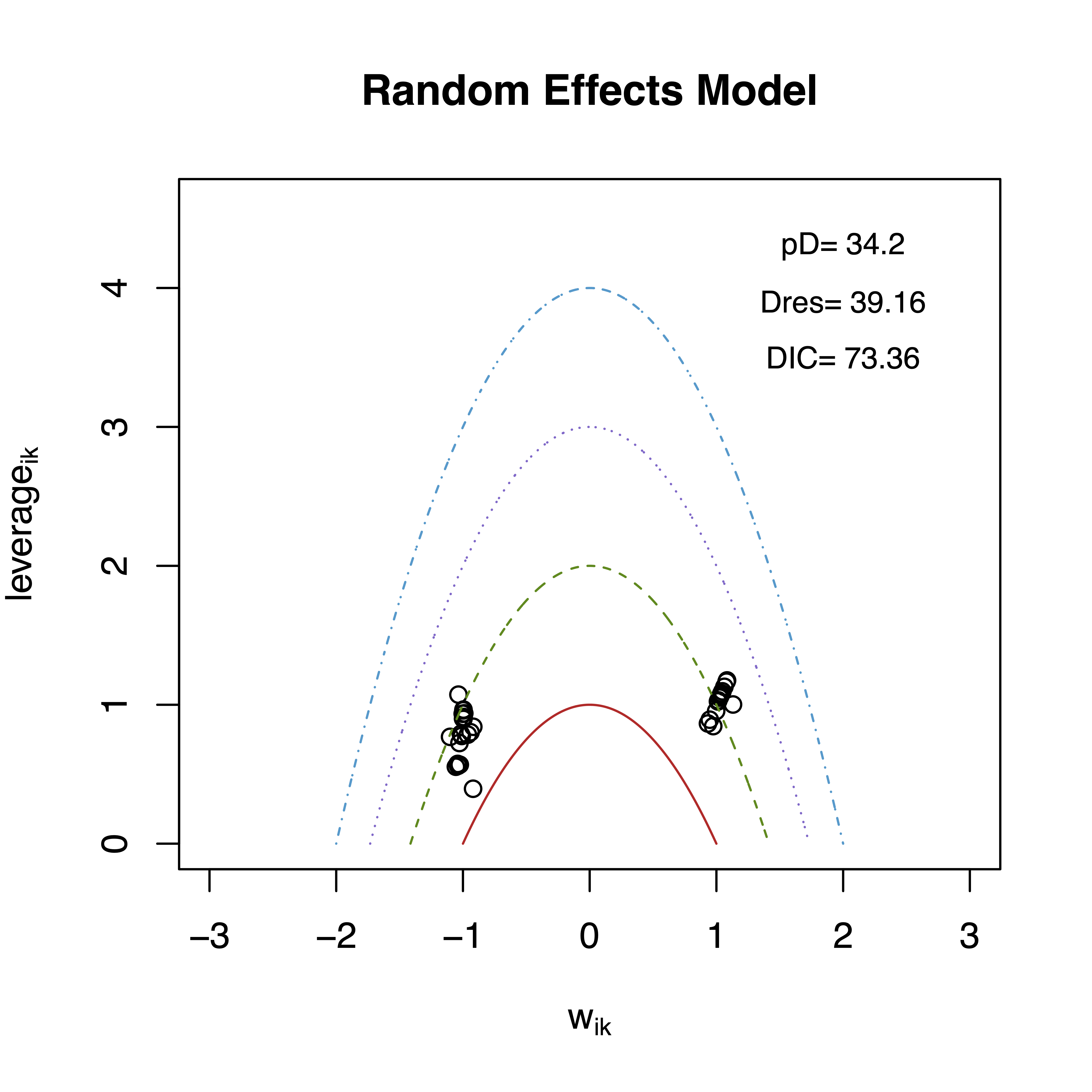

Supplement: Supplementary Figure 1 — Risk of bias of the included studies: (A) overall plot and (B) traffic light plot. [file Data_Sheet_2.ZIP › Suppl figures/Supplementary figure 2 (F).jpg]

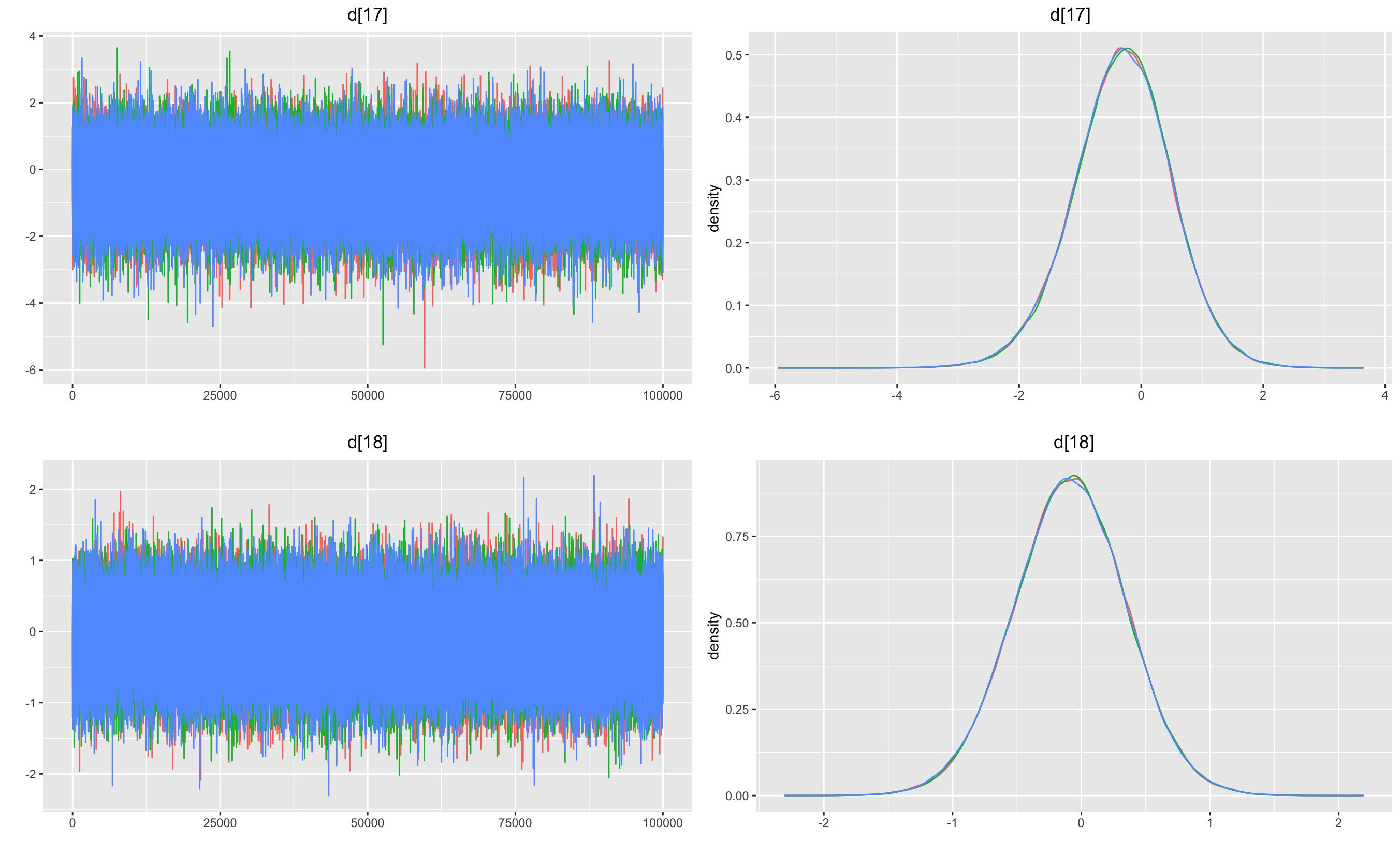

Supplement: Supplementary Figure 1 — Risk of bias of the included studies: (A) overall plot and (B) traffic light plot. [file Data_Sheet_2.ZIP › Suppl figures/Supplementary figure 3 (A).jpg]

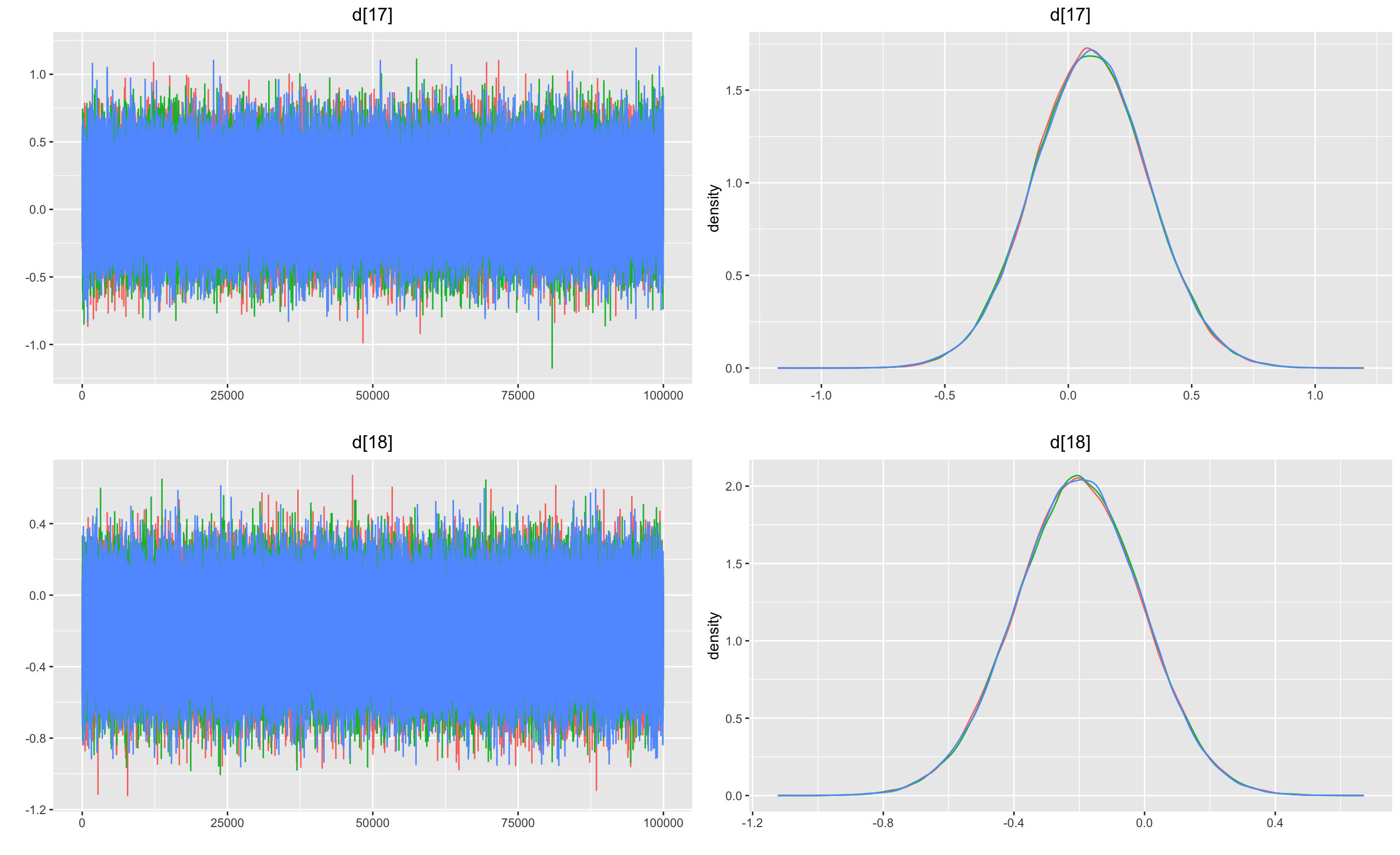

Supplement: Supplementary Figure 1 — Risk of bias of the included studies: (A) overall plot and (B) traffic light plot. [file Data_Sheet_2.ZIP › Suppl figures/Supplementary figure 3 (B).jpg]

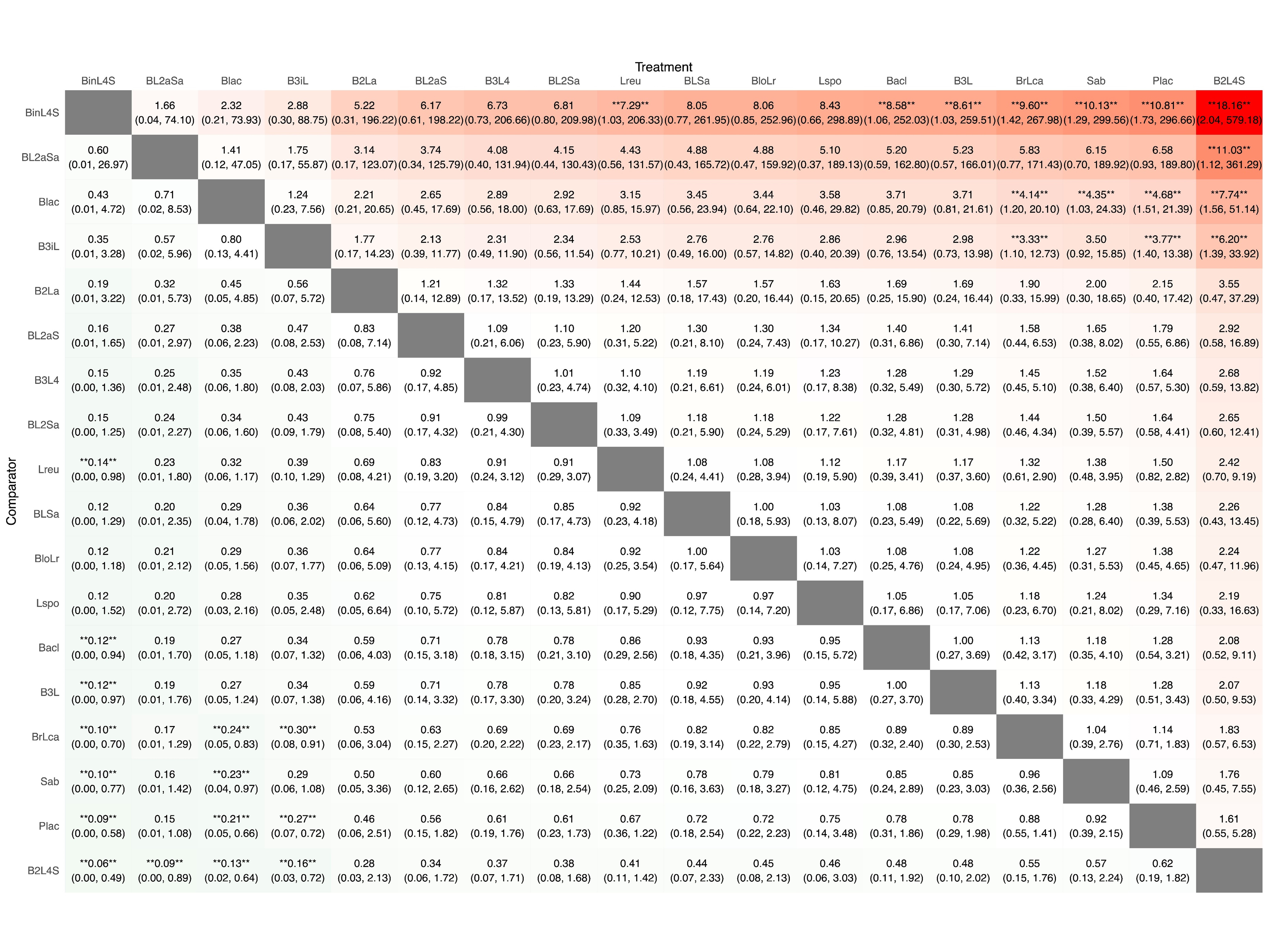

Supplement: Supplementary Figure 1 — Risk of bias of the included studies: (A) overall plot and (B) traffic light plot. [file Data_Sheet_2.ZIP › Suppl figures/Supplementary figure 4 (A).jpg]

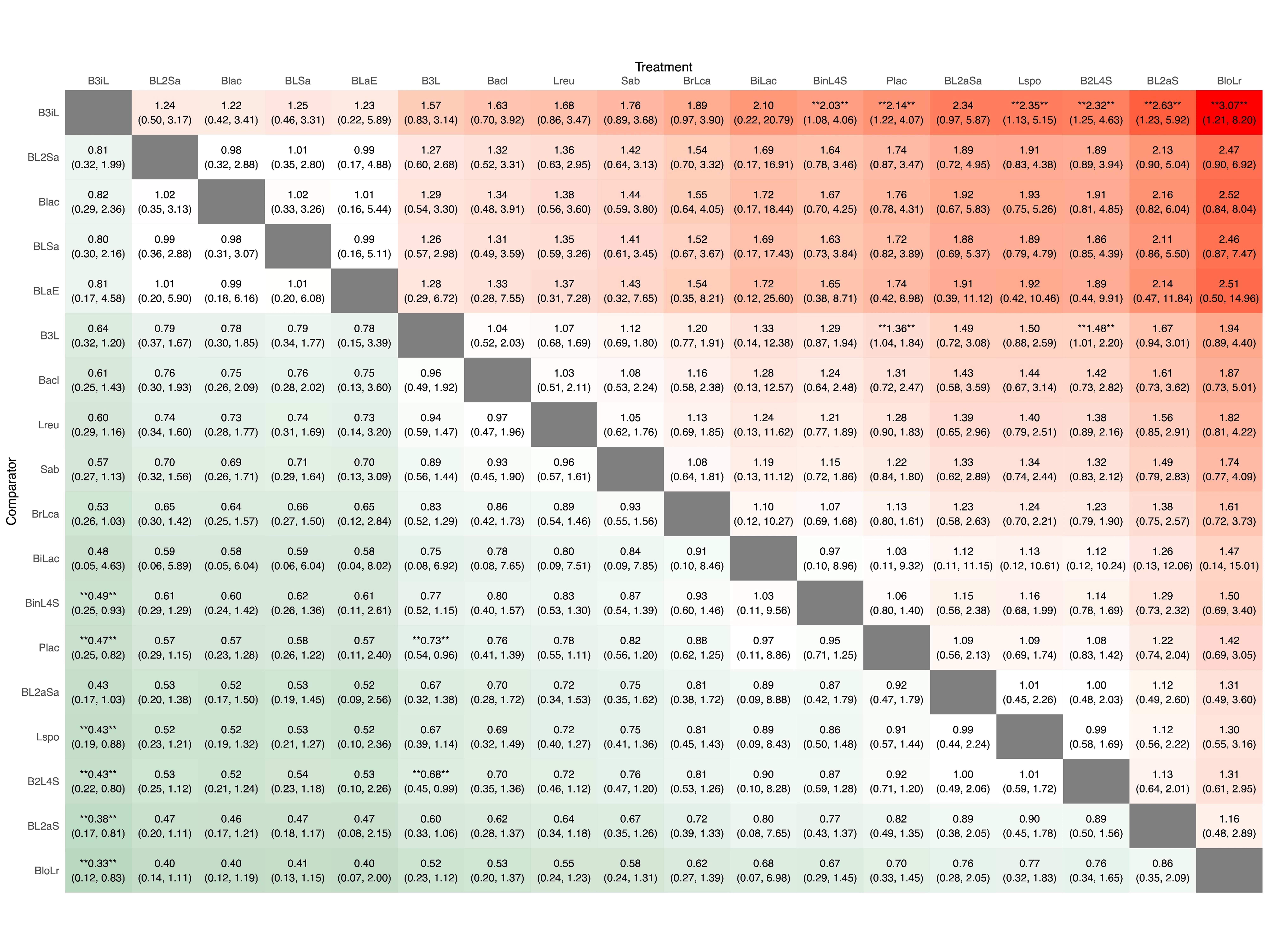

Supplement: Supplementary Figure 1 — Risk of bias of the included studies: (A) overall plot and (B) traffic light plot. [file Data_Sheet_2.ZIP › Suppl figures/Supplementary figure 4 (B).jpg]

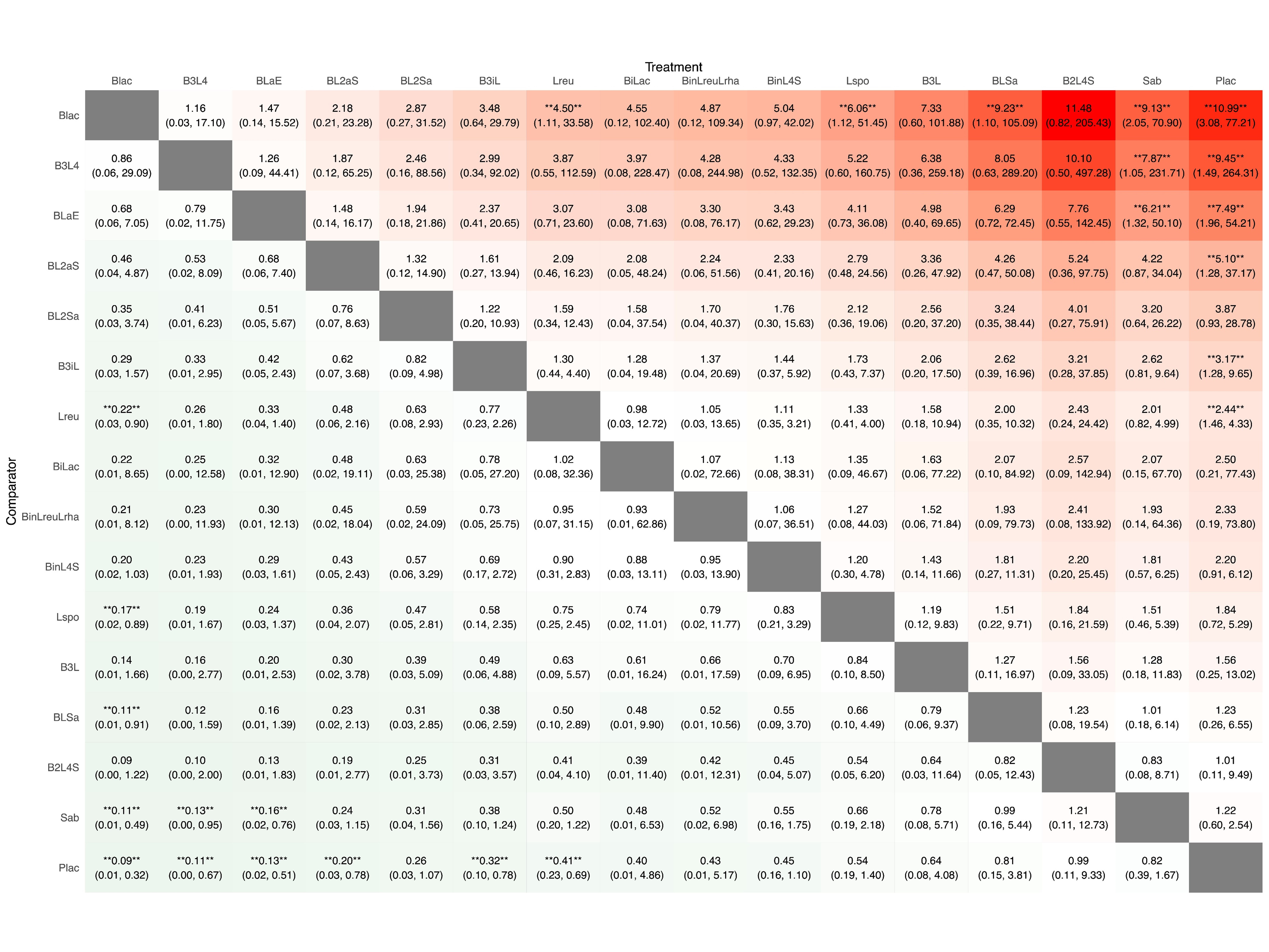

Supplement: Supplementary Figure 1 — Risk of bias of the included studies: (A) overall plot and (B) traffic light plot. [file Data_Sheet_2.ZIP › Suppl figures/Supplementary figure 4 (C).jpg]

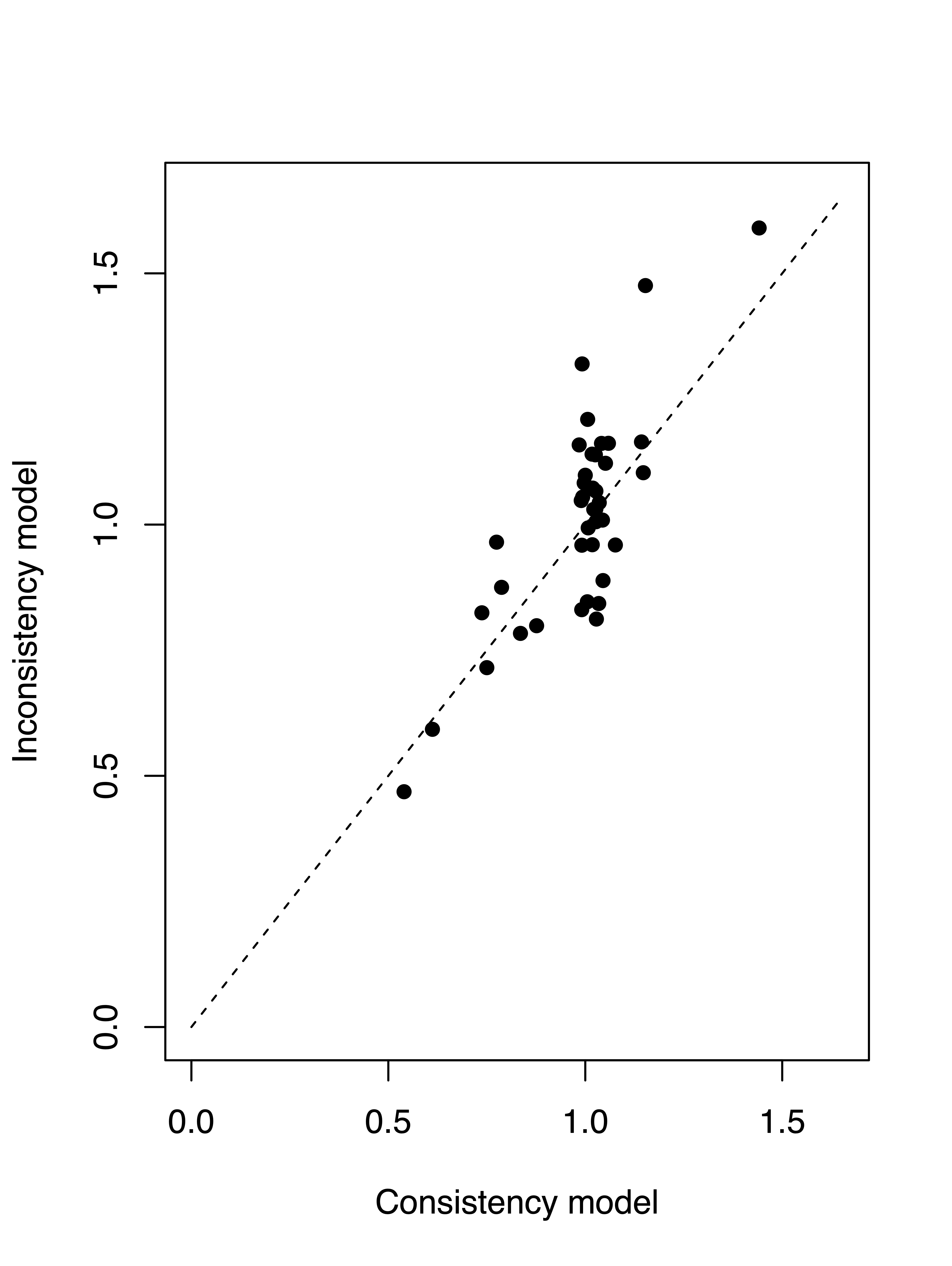

Supplement: Supplementary Figure 1 — Risk of bias of the included studies: (A) overall plot and (B) traffic light plot. [file Data_Sheet_2.ZIP › Suppl figures/Supplementary figure 5 (A).jpg]

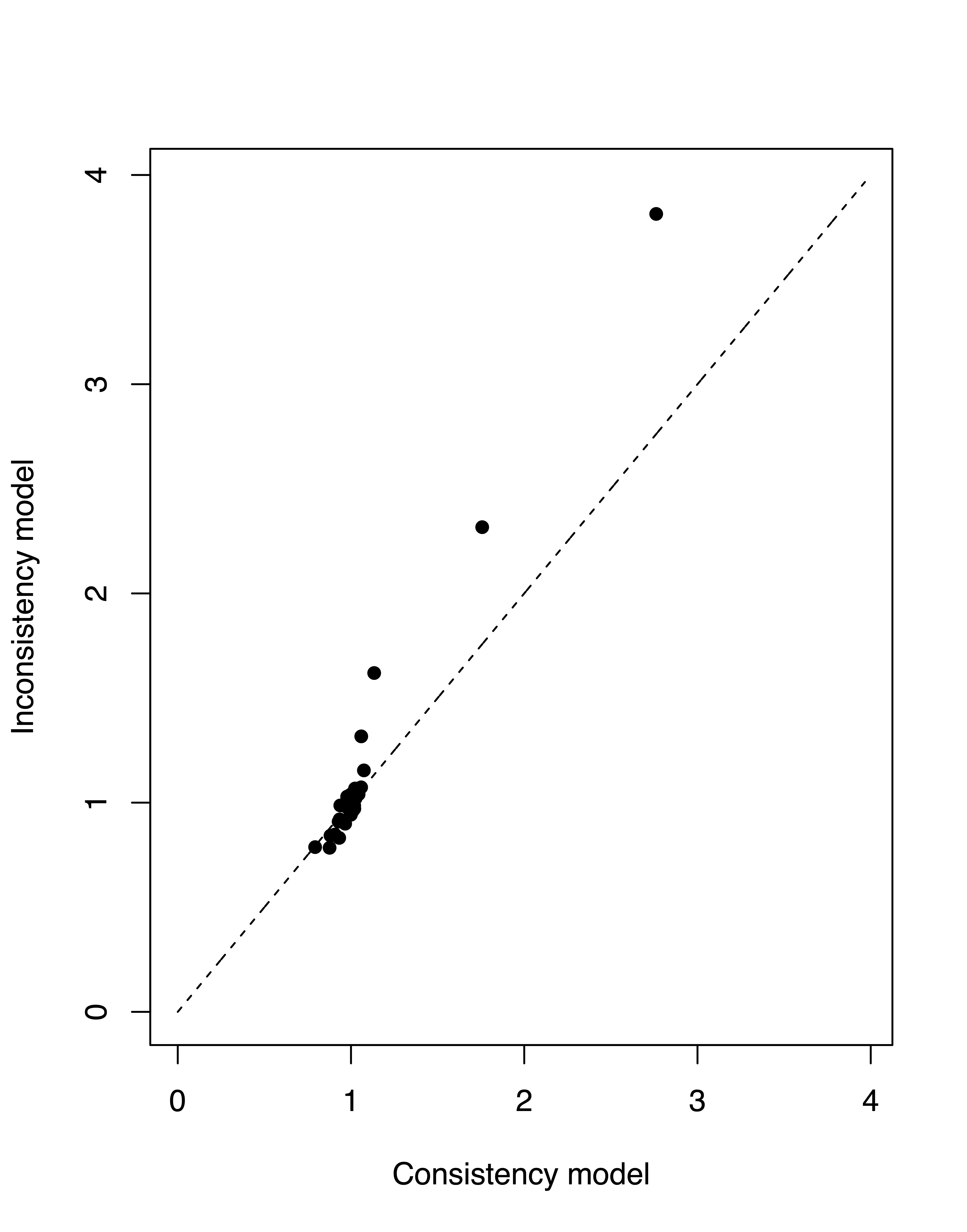

Supplement: Supplementary Figure 1 — Risk of bias of the included studies: (A) overall plot and (B) traffic light plot. [file Data_Sheet_2.ZIP › Suppl figures/Supplementary figure 5 (B).jpg]

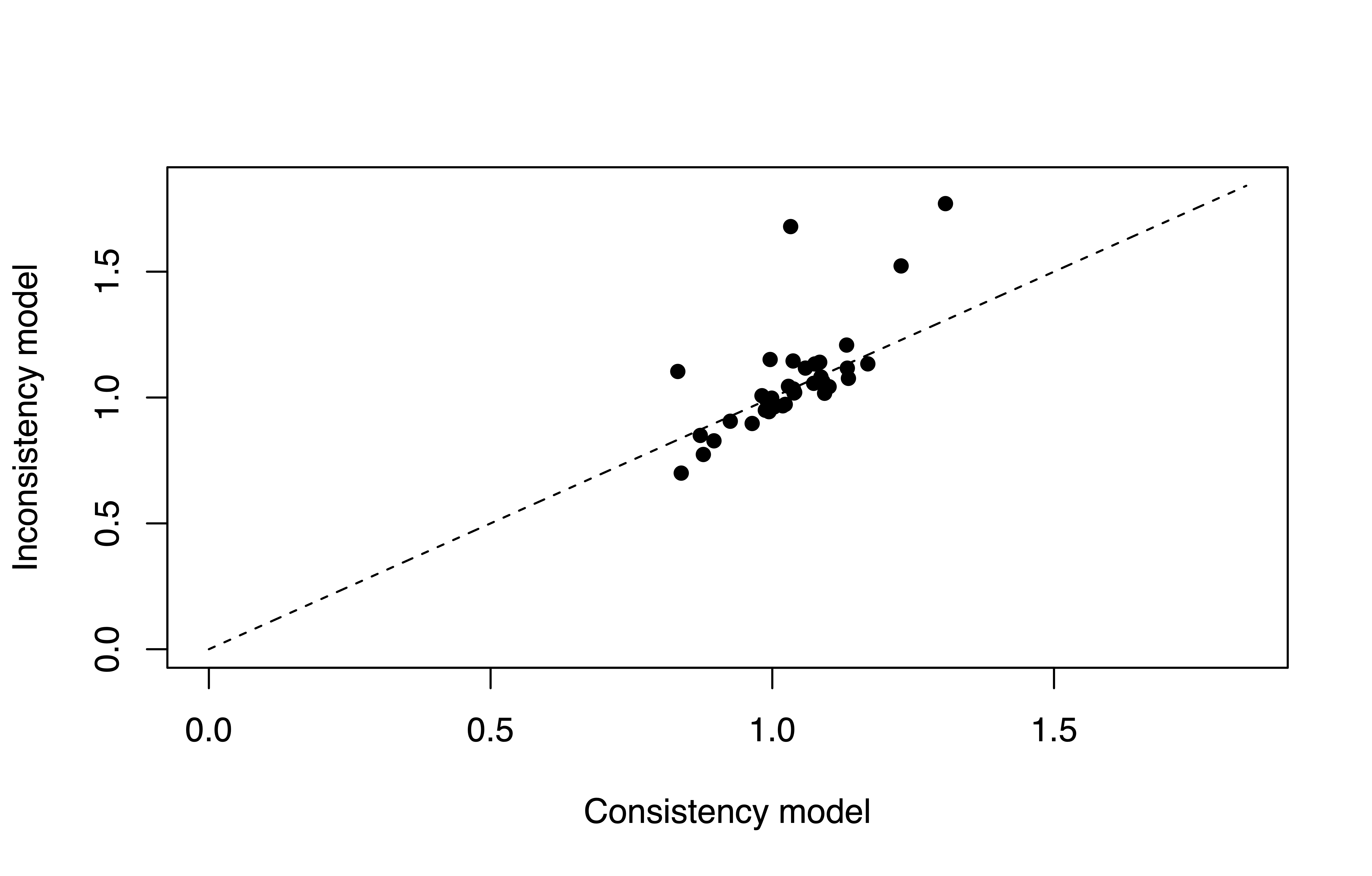

Supplement: Supplementary Figure 1 — Risk of bias of the included studies: (A) overall plot and (B) traffic light plot. [file Data_Sheet_2.ZIP › Suppl figures/Supplementary figure 5 (C).jpg]

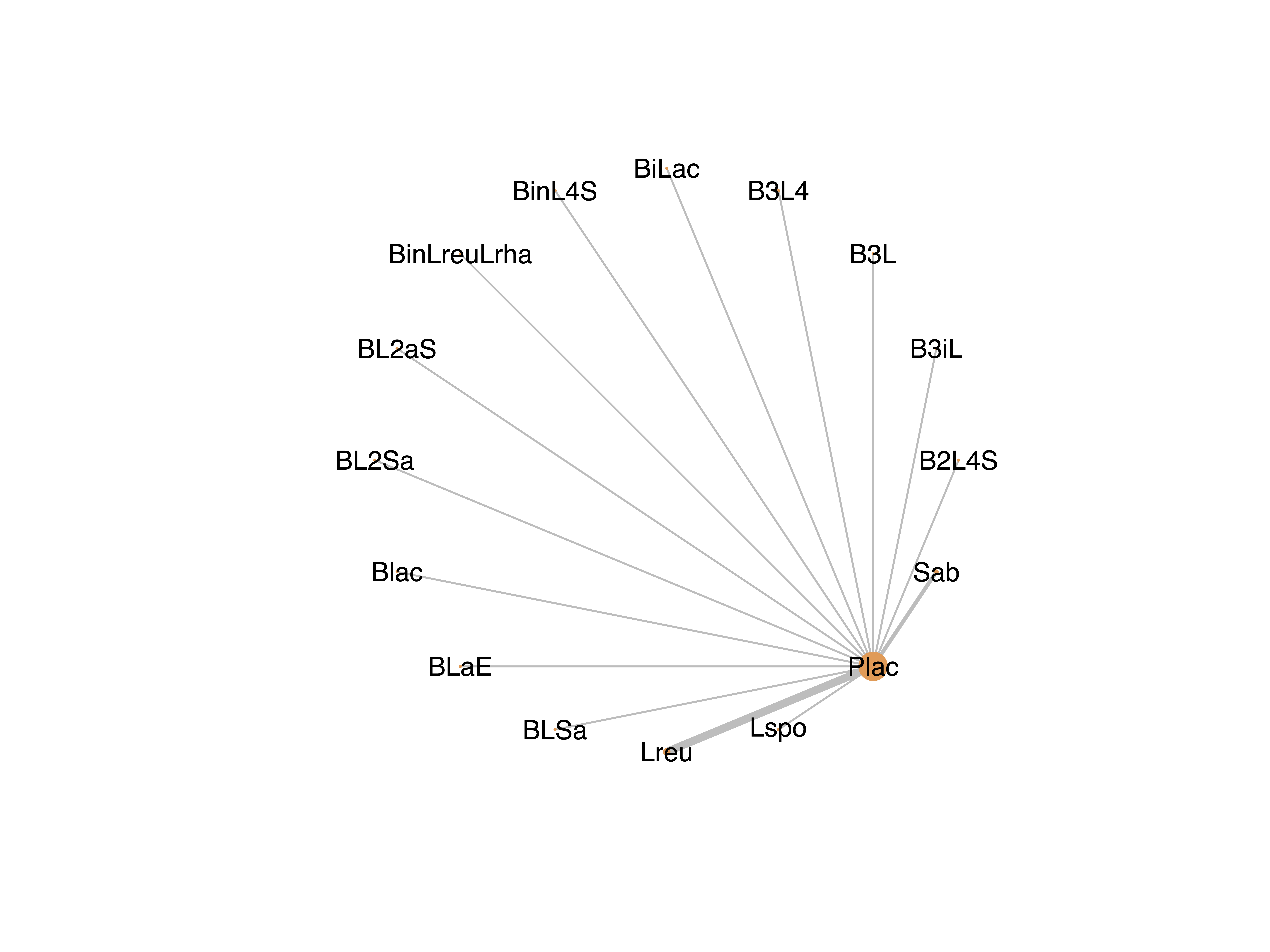

Supplement: Supplementary Figure 1 — Risk of bias of the included studies: (A) overall plot and (B) traffic light plot. [file Data_Sheet_2.ZIP › Suppl figures/Supplementary figure 6.jpg]

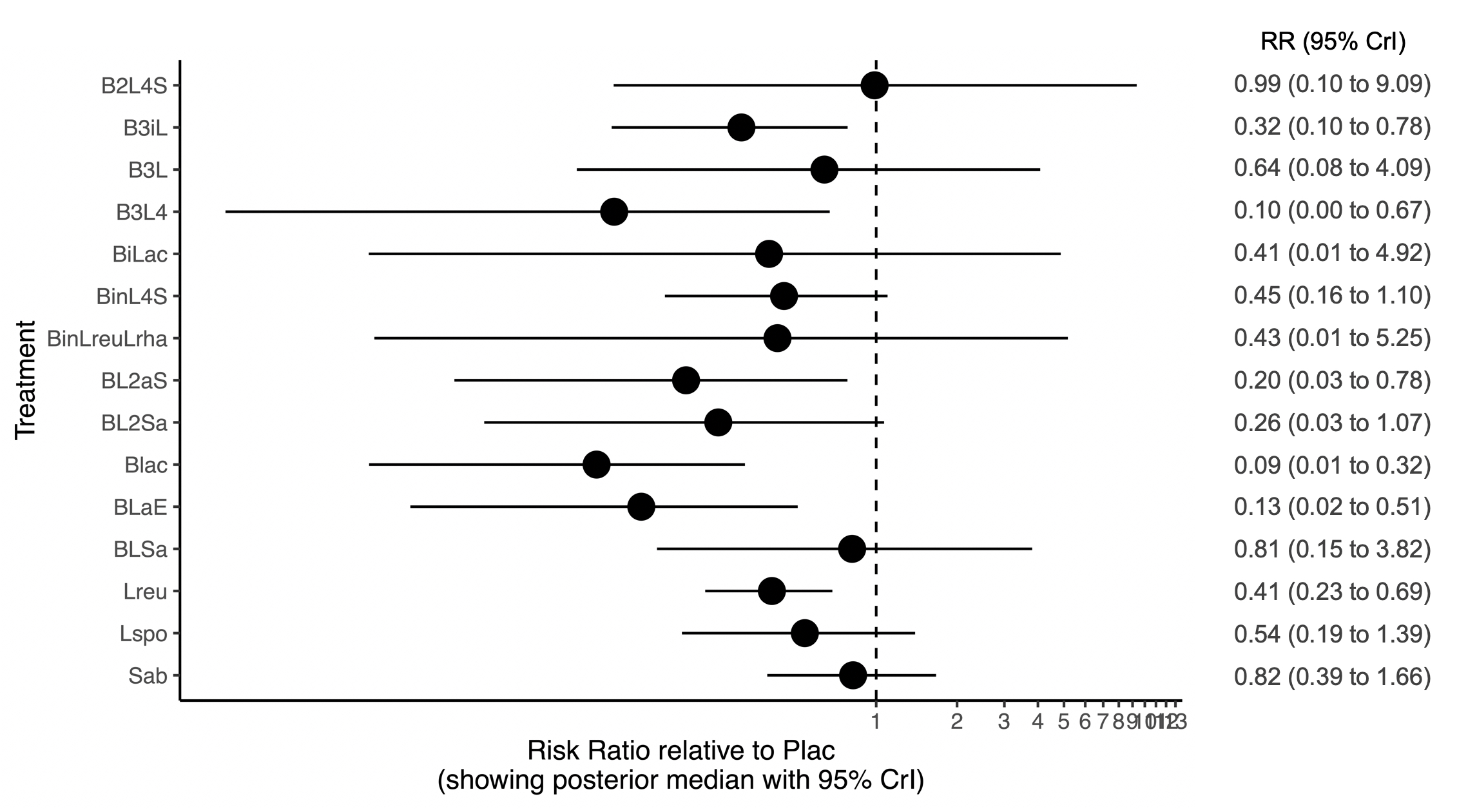

Supplement: Supplementary Figure 1 — Risk of bias of the included studies: (A) overall plot and (B) traffic light plot. [file Data_Sheet_2.ZIP › Suppl figures/Supplementary Figure 7_NEC_Forest.jpg]

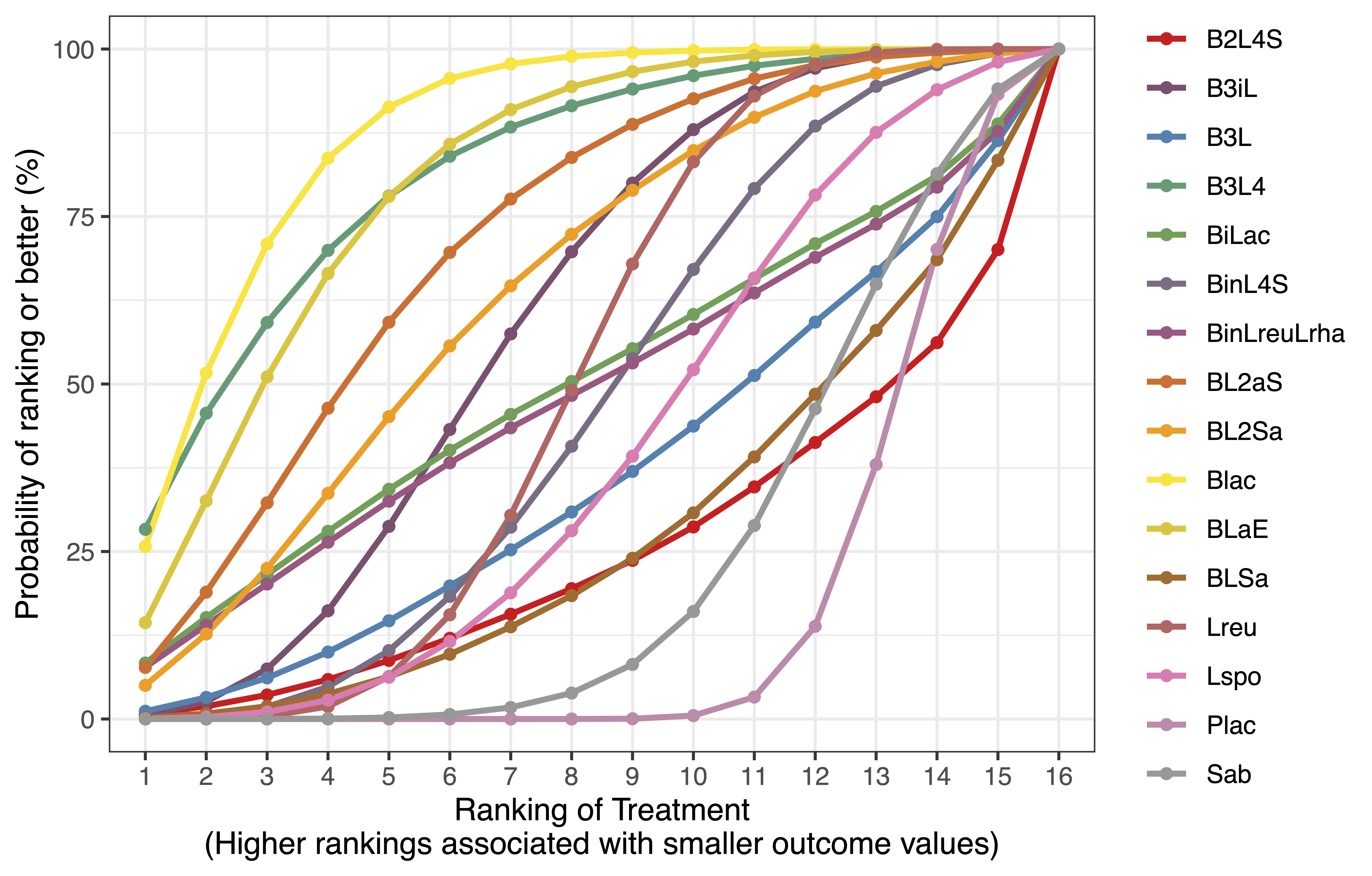

Supplement: Supplementary Figure 1 — Risk of bias of the included studies: (A) overall plot and (B) traffic light plot. [file Data_Sheet_2.ZIP › Suppl figures/Supplementary Figure 8.jpg]

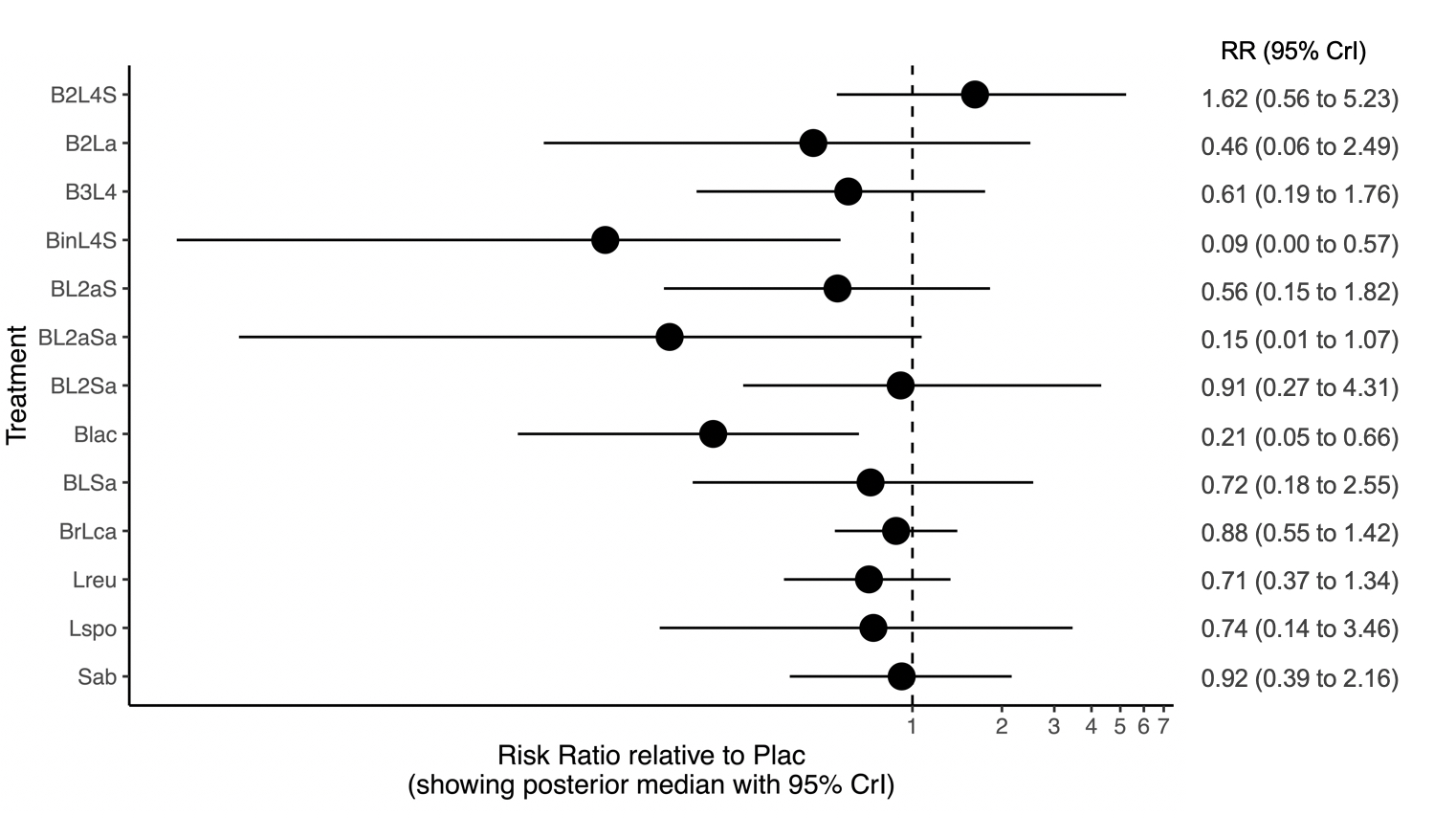

Supplement: Supplementary Figure 1 — Risk of bias of the included studies: (A) overall plot and (B) traffic light plot. [file Data_Sheet_2.ZIP › Suppl figures/Supplementary figure 9 (A).jpg]

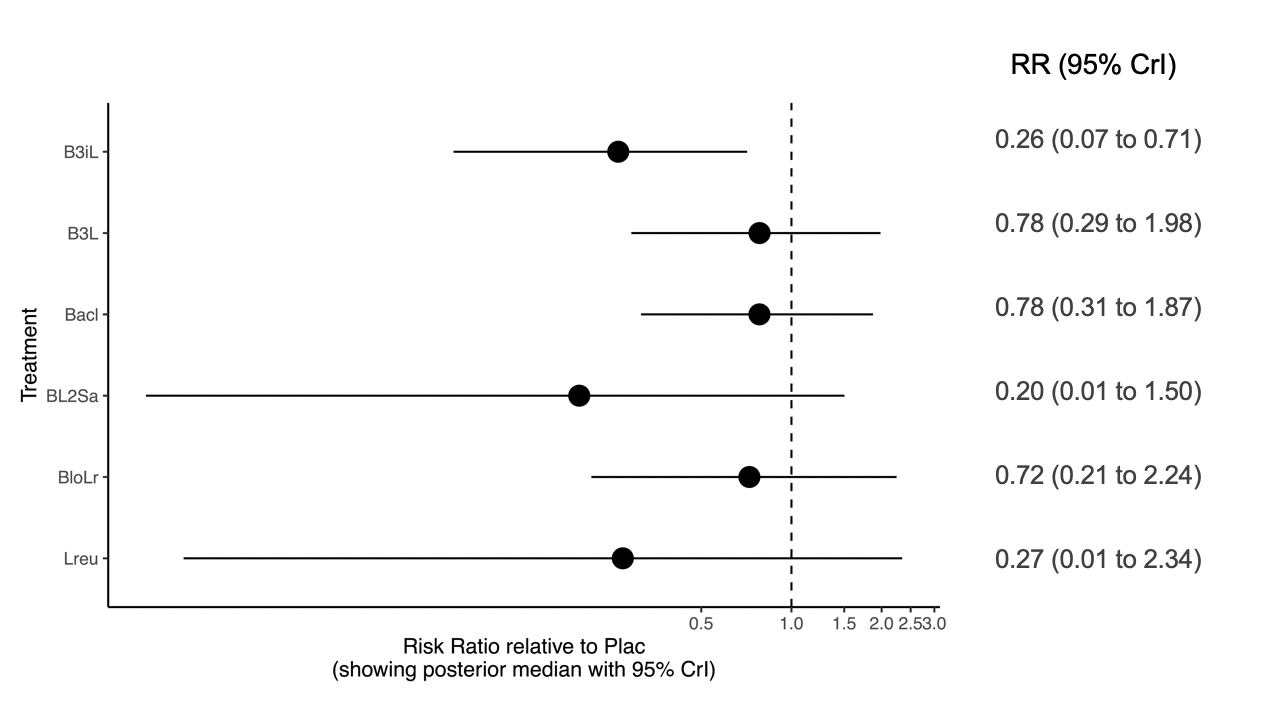

Supplement: Supplementary Figure 1 — Risk of bias of the included studies: (A) overall plot and (B) traffic light plot. [file Data_Sheet_2.ZIP › Suppl figures/Supplementary figure 9 (B).jpg]
