# Supplementary material for: Probiotics for the prevention of mortality and sepsis in preterm very low birth weight neonates from low- and middle-income countries: a Bayesian network meta-analysis
Source: Front Nutr. 2023 Jun 14;10:1133293. doi: 10.3389/fnut.2023.1133293 (PMC10300419; doi:10.3389/fnut.2023.1133293)
Supplement: Supplementary Table 1 — Deviations in the protocol. [file Data_Sheet_1.zip › Supplementary Table 1.docx]

**Supplementary Table 1: Deviations in the manuscript from the published protocol**

| **S. No** | **Section** | **Published protocol** | **Final systematic review manuscript** | **Reason for the changes** |
| --- | --- | --- | --- | --- |
|  | Type of study | Randomized controlled trials will be included | The review included both RCTs and quasi RCTs | To be more inclusive. A few studies from low-and middle-income countries were quasi-RCTs. |
|  | Search strategy | MEDLINE via Pubmed, Embase and CENTRAL will be searched | References of previous network meta-analysis were hand-searched to identify missing articles | Not to miss eligible articles identified by previous systematic reviews on the topic |
|  | Patient population | Very preterm (<32 weeks) or very low birth weight neonates (VLBW; <1500 g) | In addition to the studies that included *only* VLBW or very preterm neonates, we also included those that enrolled more mature or heavier neonates provided the mean gestation of the neonates was <32 weeks or birth weight was <1.5 kg. If the gestation/ birthweight data was unavailable, at least 50% of neonates must have been born before 32 weeks or had a birth weight of <1.5 kg to be eligible for inclusion. | To be more inclusive and comprehensive in identification of eligible studies |
|  | Patient population | Country not specified | Studies belonging to low- and middle-income countries (LMIC) were included in the final analysis. | Given the focus of the current Research Topic – low- and middle-income countries – we restricted the scope of our review to only LMICs. |
|  | Exclusion | Not specified | Studies with crossover design were be excluded | The outcomes of our review – mortality, sepsis, and NEC – cannot be addressed by cross-over trials. |
|  | Strategy for data synthesis | No mention regarding studies with zero event in any arm | Studies with zero events in any one of the arms were dropped. | Studies with zero events in one of the arms could result in imprecise estimates that could affect the ranking probabilities and other results of a network meta-analysis. |
