# Supplementary material for: Probiotics for the prevention of mortality and sepsis in preterm very low birth weight neonates from low- and middle-income countries: a Bayesian network meta-analysis
Source: Front Nutr. 2023 Jun 14;10:1133293. doi: 10.3389/fnut.2023.1133293 (PMC10300419; doi:10.3389/fnut.2023.1133293)
Supplement: Supplementary Table 1 — Deviations in the protocol. [file Data_Sheet_1.zip › Supplementary Table 2.docx]

**Supplementary table 2: Search strategy from electronic databases**

| Database | Query | Search terms | Hits |
| --- | --- | --- | --- |
| Medline (via PubMed) | #1 | (((((((((Probiotics) OR ("probiotics"[Title/Abstract])) OR (bifidobacterium[MeSH Terms])) OR (bifidobacterium[Title/Abstract])) OR (lactobacillus[MeSH Terms])) OR ("lactobacillus"[Title/Abstract])) OR (saccharomyces)) OR ("saccharomyces"[Title/Abstract])) OR ((((((((((((prebiotics) OR ("prebiotics"[Title/Abstract])) OR (oligosaccharides)) OR ("oligosaccharides"[Title/Abstract])) OR ("galactooligosaccharides"[Title/Abstract])) OR ("fructooligosaccharides"[Title/Abstract])) OR (lactulose)) OR ("lactulose"[Title/Abstract])) OR (inulin)) OR ("inulin"[Title/Abstract])) OR (lactoferrin)) OR (Lactoferrin[Title/Abstract]))) OR (((synbiotics) OR ("synbiotic"[Title/Abstract])) OR ((Probiotic[Title/Abstract]) AND (Prebiotic[Title/Abstract]))) | 3,45,932 |
|  | #2 | ((((((((((infant, newborn[MeSH Terms]) OR (premature birth)) OR (neonat*[Title/Abstract])) OR ((("newborn"[Title/Abstract]) OR ("new born"[Title/Abstract]) ) OR ("newly born"[Title/Abstract]))) OR (((("preterm"[Title/Abstract]) OR ("preterms"[Title/Abstract]) ) OR ("pre term"[Title/Abstract])) OR ("pre terms"[Title/Abstract]))) OR ((((preemie*[Title/Abstract]) ) OR (premie[Title/Abstract])) OR (premies[Title/Abstract]))) OR ((Prematur*[Title/Abstract]) AND (((Birth*[Title/Abstract]) OR ("born"[Title/Abstract])) OR (deliver*[Title/Abstract])))) OR ((Low birthweight*[Title/Abstract]) OR (Low birth weight*[Title/Abstract]))) OR ((("lbw"[Title/Abstract]) OR ("vlbw"[Title/Abstract])) OR ("elbw"[Title/Abstract]))) OR (infant*[Title/Abstract])) OR ((baby[Title/Abstract]) OR ("babies"[Title/Abstract])) | 11,32,787 |
|  | #3 | (((((((("randomized controlled trial"[Publication Type]) OR ("controlled clinical trial"[Publication Type])) OR ("randomized"[Title/Abstract])) OR ("placebo"[Title/Abstract])) OR (Drug Therapy[MeSH Subheading])) OR ("randomly"[Title/Abstract])) OR ("trial"[Title/Abstract])) OR ("groups"[Title/Abstract])) NOT ((animals[MeSH Terms]) NOT (humans[MeSH Terms])) | 46,64,014 |
|  | #4 | #1 AND #2 AND #3 AND ("1946/01/01"[Date - Publication] : "2022/07/31"[Date - Publication]) | 3050 |
| Embase | #1 | ('probiotic agent' OR 'probiotic':ti,ab,kw OR 'bifidobacterium'/exp OR 'bifidobacterium':ti,ab,kw OR 'lactobacillus'/exp OR 'lactobacillus':ti,ab,kw OR ('saccharomyces' OR 'saccharomyces boulardii' OR 'saccharomyces cerevisiae') OR 'saccharomyces':ti,ab,kw) OR ('prebiotic agent' OR 'prebiotic':ti,ab,kw OR 'oligosaccharide'/exp OR 'oligosaccharide':ti,ab,kw OR 'galactose oligosaccharide' OR ('galacto-oligosaccharide':ti,ab,kw OR 'galactooligosaccharide':ti,ab,kw) OR ('fructose oligosaccharide' OR 'fructo-oligosaccharide':ti,ab,kw OR 'fructooligosaccharide':ti,ab,kw OR 'fos':ti,ab,kw OR 'foss':ti,ab,kw) OR 'lactulose' OR 'lactulose':ti,ab,kw OR 'inulin' OR 'inulin':ti,ab,kw OR 'lactoferrin' OR 'lactoferrin':ti,ab,kw) OR ('synbiotic agent' OR 'synbiotic':ti,ab,kw OR (probiotic$:ti,ab,kw AND prebiotic$:ti,ab,kw)) | 948913 |
|  | #2 | newborn OR 'prematurity' OR ('neonat$':ti,ab,kw OR 'neo nat$':ti,ab,kw) OR ('newborn$':ti,ab OR 'new born$':ti,ab OR 'newly born$':ti,ab) OR ('preterm':ti,ab OR 'preterms':ti,ab OR 'pre term':ti,ab OR 'pre terms':ti,ab) OR ('preemie*':ti,ab OR 'preemie':ti,ab OR 'preemies':ti,ab) OR (prematur* NEXT/3 (birth* OR born OR deliver*)):ti,ab OR ('low birthweight':ti,ab OR 'low birth weights':ti,ab) OR ('lbw':ti,ab OR 'vlbw':ti,ab OR 'elbw':ti,ab) OR 'infan*':ti,ab OR ('baby':ti,ab OR 'babies':ti,ab) | 1315296 |
|  | #3 | ('randomized controlled trial' OR 'controlled clinical trial' OR 'random*':ti,ab OR 'randomization' OR 'intermethod comparison' OR 'placebo':ti,ab OR ('compare':ti OR 'compared':ti OR 'comparison':ti) OR ((evaluated:ab OR evaluate:ab OR evaluating:ab OR assessed:ab OR assess:ab) AND (compare:ab OR compared:ab OR comparing:ab OR comparison:ab)) OR (open NEXT/1 label):ti,ab OR ((double OR single OR doubly OR singly) NEXT/1 (blind OR blinded OR blindly)):ti,ab OR 'double blind procedure' OR 'parallel group*':ti,ab OR (crossover:ti,ab OR 'cross over':ti,ab) OR ((assign* OR match OR matched OR allocation) NEXT/5 (alternate OR group* OR intervention* OR patient* OR subject* OR participant*)):ti,ab OR (assigned:ti,ab OR allocated:ti,ab) OR (controlled NEXT/7 (study OR design OR trial)):ti,ab OR (volunteer:ti,ab OR volunteers:ti,ab) OR 'human experiment') NOT (((random* NEXT/1 sampl* NEXT/7 ('cross section*' OR questionnaire* OR survey* OR database*)):ti,ab NOT ('comparative study' OR 'controlled study' OR 'randomized controlled':ti,ab OR 'randomly assigned':ti,ab)) OR ('cross-sectional study' NOT ('randomized controlled trial' OR 'controlled clinical study' OR 'controlled study' OR 'randomized controlled':ti,ab OR 'control group*':ti,ab)) OR (((case NEXT/1 control*) AND random*) NOT 'randomized controlled trial') OR ('systematic review' NOT (trial:ti OR study:ti)) OR (nonrandom*:ti,ab NOT random*:ti,ab) OR 'random field*':ti,ab OR ('random-cluster' NEXT/3 'sampl*'):ti,ab OR (('review':ab AND [review]/lim) NOT trial:ti) OR ('we searched':ab AND (review:ti OR [review]/lim)) OR 'update review':ab OR (databases NEXT/4 searched):ab OR ((rat:ti OR rats:ti OR mouse:ti OR mice:ti OR swine:ti OR porcine:ti OR murine:ti OR sheep:ti OR lambs:ti OR pigs:ti OR piglets:ti OR rabbit:ti OR rabbits:ti OR cat:ti OR cats:ti OR dog:ti OR dogs:ti OR cattle:ti OR bovine:ti OR monkey:ti OR monkeys:ti OR trout:ti OR marmoset*:ti) AND 'animal experiment') OR ('animal experiment' NOT ('human experiment' OR human))) | 5120253 |
|  | #4 | #1 AND #2 AND #3 AND [1974-2021]/py | 6400 |
| CENTRAL | #1 | MeSH descriptor: [Probiotics] explode all trees OR (probiotic*):ti,ab,kw OR MeSH descriptor: [Bifidobacterium] explode all trees OR (bifidobacterium*):ti,ab,kw OR MeSH descriptor: [Lactobacillus] explode all trees OR (lactobacill*):ti,ab,kw OR MeSH descriptor: [Saccharomyces boulardii] OR (Saccharomyces*):ti,ab,kw OR MeSH descriptor: [Prebiotics] explode all trees OR (Prebiotic*):ti,ab,kw OR MeSH descriptor: [Oligosaccharides] explode all trees OR (oligosaccharide*):ti,ab,kw OR MeSH descriptor: [Inulin] explode all trees OR (Inulin):ti,ab,kw OR (fructooligosaccharide* or fructo-oligosaccharide* or FOS or FOSs or galacto-oligosaccharide* or galactooligosaccharide*):ti,ab,kw OR MeSH descriptor: [Lactoferrin] explode all trees OR (lactoferrin*):ti,ab,kw OR MeSH descriptor: [Lactulose] explode all trees OR (lactulose*):ti,ab,kw OR MeSH descriptor: [Synbiotics] explode all trees OR (synbiotic*):ti,ab,kw OR (((probiotic* and prebiotic*) NEAR/4 combin*)):ti,ab,kw | 17577 |
|  | #2 | MeSH descriptor: [Infant, Newborn] explode all trees OR MeSH descriptor: [Premature Birth] explode all trees OR (neonat*):ti,ab,kw OR (neo-nat*):ti,ab,kw OR (newborn or new born* or newly born*):ti,ab,kw OR (preterm or preterms or (pre term) or (pre terms)):ti,ab,kw OR (preemie* or premie or premies):ti,ab,kw OR (prematur* near/3 (birth* or born or deliver*)):ti,ab,kw OR (low near/3 (birthweight* or birth weight*)):ti,ab,kw OR (lbw or vlbw or elbw):ti,ab,kw OR (infan* or baby or babies):ti,ab,kw | 106768 |
|  | #3 | #1 AND #2 | 3268 |
