# Supplementary material for: Probiotics for the prevention of mortality and sepsis in preterm very low birth weight neonates from low- and middle-income countries: a Bayesian network meta-analysis
Source: Front Nutr. 2023 Jun 14;10:1133293. doi: 10.3389/fnut.2023.1133293 (PMC10300419; doi:10.3389/fnut.2023.1133293)
Supplement: Supplementary Table 1 — Deviations in the protocol. [file Data_Sheet_1.zip › Supplementary Table 3.docx]

**Supplementary table 3: List of excluded studies after full text screening**

| S. No. | Study reference | Reasons for exclusion |
| --- | --- | --- |
|  | Martí M, Spreckels JE, Ranasinghe PD, Wejryd E, Marchini G, Sverremark-Ekström E, Jenmalm MC, Abrahamsson T. Effects of Lactobacillus reuteri supplementation on the gut microbiota in extremely preterm infants in a randomized placebo-controlled trial. Cell Rep Med. 2021 Feb 22;2(3):100206. doi: 10.1016/j.xcrm.2021.100206. PMID: 33763652; PMCID: PMC7974321. | Different outcome of a study which is excluded as not done in LMIC |
|  | Oncel MY, Arayici S, Sari FN, Simsek GK, Yurttutan S, Erdeve O, Saygan S, Uras N, Oguz SS, Dilmen U. Comparison of Lactobacillus reuteri and nystatin prophylaxis on Candida colonization and infection in very low birth weight infants. J Matern Fetal Neonatal Med. 2015;28(15):1790-4. doi: 10.3109/14767058.2014.968842. Epub 2014 Oct 9. PMID: 25245226. | Wrong comparator |
|  | Moreno-Sanz B, Montes MT, Manzano S, Espinosa-Martos I, Cárdenas N, Esteban S, Cruz M, Jiménez E, de Pipaón MS. Randomized, Double-Blind, Placebo-Controlled Study to Assess the Effect of Two Probiotics on the Preterms' Gut Microbiota. J Pediatr Gastroenterol Nutr. 2022 Jun 1;74(6):e153-e159. doi: 10.1097/MPG.0000000000003427. Epub 2022 Feb 25. PMID: 35221319. | Wrong outcomes |
|  | Dehghan K, Karimi S, Alilu L. The Effect of Probiotics on Late-Onset Sepsis in Very Preterm Infants: A Randomized Clinical Trial. Int J Pediatr 2018; 6(10): 8371-79. DOI: 10.22038/ijp.2018.31304.2768. | Wrong intervention |
|  | Underwood MA, Kalanetra KM, Bokulich NA, Lewis ZT, Mirmiran M, Tancredi DJ, Mills DA. A comparison of two probiotic strains of bifidobacteria in premature infants. J Pediatr. 2013 Dec;163(6):1585-1591.e9. doi: 10.1016/j.jpeds.2013.07.017. Epub 2013 Aug 29. PMID: 23993139; PMCID: PMC3842430. | Wrong study design |
|  | Watkins C, Murphy K, Dempsey EM, O'Shea CA, Murphy BP, O'Toole PW, Ross RP, Stanton C, Ryan CA. Dose-interval study of a dual probiotic in preterm infants. Arch Dis Child Fetal Neonatal Ed. 2019 Mar;104(2):F159-F164. doi: 10.1136/archdischild-2017-313468. Epub 2018 Jun 20. PMID: 29925539. | Wrong study design |
|  | Plummer EL, Bulach DM, Murray GL, Jacobs SE, Tabrizi SN, Garland SM; ProPrems Study Group. Gut microbiota of preterm infants supplemented with probiotics: sub-study of the ProPrems trial. BMC Microbiol. 2018 Nov 13;18(1):184. doi: 10.1186/s12866-018-1326-1. PMID: 30424728; PMCID: PMC6234596. | Different outcome of a study which is excluded as not done in LMIC |
|  | Vakiliamini M, Babaei H, Mohammadi M, Habibi R, Motamed H. Intestinal Colonization Rate of Candida albicans among Low Birth Weight Neonates after Using Oral Synbiotic Supplementation: A Randomized Placebo-controlled Trial. Iranian Journal of Neonatology. 2020 Sep: 11(3). DOI: 10.22038/ijn.2020.40131.1651 | Wrong intervention |
|  | Woodman, T.; Strunk, T.; Simmer, K.; Currie, A. Evaluating the combintion of bovine lactoferrin and bifidobacterium breve m-16v in human milk for prevention of infection and inflammation in preterm neonates. ournal of Paediatrics and Child Health 2017;53 | Wrong intervention |
|  | Patole S, Keil AD, Chang A, Nathan E, Doherty D, Simmer K, Esvaran M, Conway P. Effect of Bifidobacterium breve M-16V supplementation on fecal bifidobacteria in preterm neonates--a randomised double blind placebo controlled trial. PLoS One. 2014 Mar 3;9(3):e89511. doi: 10.1371/journal.pone.0089511. PMID: 24594833; PMCID: PMC3940439. | Wrong outcomes |
|  | Fleming P, Panton N, Wilks M, Millar M, Costeloe K. Comparing serum bacterial DNA and lipopolysaccharide in preterm infants randomised to bifidobacterium breve BBG-001 versus placebo. Journal of paediatrics and child health, 2015, 51, 67- | Different outcome of a study which is excluded as not done in LMIC |
|  | Yamasaki C, Totsu S, Uchiyama A, Nakanishi H, Masumoto K, Washio Y, Shuri K, Ishida S, Imai K, Kusuda S. Effect of Bifidobacterium administration on very-low-birthweight infants. Pediatr Int. 2012 Oct;54(5):651-6. doi: 10.1111/j.1442-200X.2012.03649.x. Epub 2012 Jul 10. PMID: 22507386. | Wrong comparator |
|  | N Zampieri, A Pietrobelli, F Camoglio,. The use of Lactobacillus paracasei in Bell's stage 2 of necrotizing enterocolitis in very low birth weight infants: Preliminary report. Early Human Development, Volume 87, Supplement, 2011, Page S98, | Wrong patient population |
|  | Millar M, Seale J, Greenland M, Hardy P, Juszczak E, Wilks M, Panton N, Costeloe K, Wade WG. The Microbiome of Infants Recruited to a Randomised Placebo-controlled Probiotic Trial (PiPS Trial). EBioMedicine. 2017 Jun;20:255-262. doi: 10.1016/j.ebiom.2017.05.019. Epub 2017 May 17. PMID: 28571671; PMCID: PMC5478240. | Different outcome of a study which is excluded as not done in LMIC |
|  | Kerry et al. The Effects of Lactoferrin Supplementation on the Intestinal Microbiota of Premature receiving Probiotics. 2014 (conference abstract) | Wrong comparator |
|  | Amini E, Dalili H, Niknafs N, Shariat M, Nakhostin M, et al. The Effect of Probiotics in Prevention of Necrotising Enterocolitis in Preterm Neonates in Comparison with Control Group. Iran J Pediatr. 2017;27(6):e7663. doi: 10.5812/ijp.7663. | Wrong intervention |
|  | Sari FN, Eras Z, Dizdar EA, Erdeve O, Oguz SS, Uras N, Dilmen U. Do oral probiotics affect growth and neurodevelopmental outcomes in very low-birth-weight preterm infants? Am J Perinatol. 2012 Sep;29(8):579-86. doi: 10.1055/s-0032-1311981. Epub 2012 May 7. PMID: 22566113. | Included study with different outcome |
|  | Agrawal S, Pestell CF, Granich J, Rao S, Nathan E, Wray JA, Whitehouse AJO, Patole S. Difficulties in developmental follow-up of preterm neonates in a randomised-controlled trial of Bifidobacterium breve M16-V - Experience from Western Australia. Early Hum Dev. 2020 Dec;151:105165. doi: 10.1016/j.earlhumdev.2020.105165. Epub 2020 Aug 22. PMID: 32871454. | Different outcome of a study which is excluded as not done in LMIC |
|  | Hu XY, Zhou YX, Xu SZ, Lin YY. [Effects of probiotics on feeding intolerance in low birth weight premature infants]. Zhongguo Dang Dai Er Ke Za Zhi. 2010 Sep;12(9):693-5. Chinese. PMID: 20849715. | Wrong patient population |
|  | Lee SJ, Cho SJ, Park EA. Effects of probiotics on enteric flora and feeding tolerance in preterm infants. Neonatology. 2007;91(3):174-9. doi: 10.1159/000097449. Epub 2006 Nov 29. PMID: 17377402. | Wrong patient population |
|  | El C, Satar M, Yildizdas H, et al O-222 Evaluation Of The Influence Of Bifidobakterium Lactis 2011 And Hindiba Inulin On Feeding Intolerance And Necrotising Enterocolitis In Premature Babies Archives of Disease in Childhood 2014;99:A110. | Wrong intervention |
|  | Cui X, Shi Y, Gao S, Xue X, Fu J. Effects of Lactobacillus reuteri DSM 17938 in preterm infants: a double-blinded randomized controlled study. Ital J Pediatr. 2019 Nov 9;45(1):140. doi: 10.1186/s13052-019-0716-9. PMID: 31706331; PMCID: PMC6842458. | Wrong patient population |
|  | Bartoschek, B.; Heinzel, E.; Meng-Hentschel, J.; Schöndorf, D.; Gortner, L.; Herrmann, M.; Von Müller, L. Culture-independent evaluation of preterm infant's gut microbiota following probiotic substitution. International Journal of Medical Microbiology 2012;302 (Conference abstract) | Wrong outcomes |
|  | Manzoni P, Stolfi I, Messner H, Cattani S, Laforgia N, Romeo MG, Bollani L, Rinaldi M, Gallo E, Quercia M, Maule M, Mostert M, Decembrino L, Magaldi R, Mosca F, Vagnarelli F, Memo L, Betta PM, Stronati M, Farina D; Italian Task Force for the Study and Prevention of Neonatal Fungal Infections–the Italian Society of Neonatology. Bovine lactoferrin prevents invasive fungal infections in very low birth weight infants: a randomized controlled trial. Pediatrics. 2012 Jan;129(1):116-23. doi: 10.1542/peds.2011-0279. Epub 2011 Dec 19. PMID: 22184648. | Wrong intervention |
|  | Li X, Peng Y, Li Z, Christensen B, Heckmann AB, Stenlund H, Lönnerdal B, Hernell O. Feeding Infants Formula With Probiotics or Milk Fat Globule Membrane: A Double-Blind, Randomized Controlled Trial. Front Pediatr. 2019 Aug 21;7:347. doi: 10.3389/fped.2019.00347. PMID: 31552203; PMCID: PMC6736587. | Wrong patient population |
|  | Dasopoulou M, Briana DD, Boutsikou T, Karakasidou E, Roma E, Costalos C, Malamitsi-Puchner A. Motilin and gastrin secretion and lipid profile in preterm neonates following prebiotics supplementation: a double-blind randomized controlled study. JPEN J Parenter Enteral Nutr. 2015 Mar;39(3):359-68. doi: 10.1177/0148607113510182. Epub 2013 Nov 14. PMID: 24233255. | Wrong intervention |
|  | Serce Pehlevan O, Benzer D, Gursoy T, Aktas Cetin E, Karatekin G, OvaliMD F. Cytokine responses to symbiotic and lactoferrin combination in very low birth weight neonates: a randomized control trial. Arch Argent Pediatr. 2020 Feb;118(1):e8-e15. English, Spanish. doi: 10.5546/aap.2020.eng.e8. PMID: 31984696. | Wrong intervention |
|  | Nandhini LP, Biswal N, Adhisivam B, Mandal J, Bhat B V, Mathai B. Synbiotics for decreasing incidence of necrotizing enterocolitis among preterm neonates - a randomized controlled trial. J Matern Fetal Neonatal Med. 2016 Mar;29(5):821-5. doi: 10.3109/14767058.2015.1019854. Epub 2015 Mar 10. PMID: 25754214. | Wrong intervention |
|  | Serce Pehlevan O, Benzer D, Gursoy T, Karatekin G, Ovali F. Synbiotics use for preventing sepsis and necrotizing enterocolitis in very low birth weight neonates: a randomized controlled trial. Clin Exp Pediatr. 2020 Jun;63(6):226-231. doi: 10.3345/cep.2019.00381. Epub 2020 Feb 5. PMID: 32023397; PMCID: PMC7303425. | Wrong intervention |
|  | Aydin B, Dilli D, Erol S, et al 1634 The Effects of Synbiotic use on Morbidity and Mortality in Premature Infants: A Prospective Randomized Controlled Trial Archives of Disease in Childhood 2012;97:A462. | Wrong intervention |
|  | Armanian AM, Sadeghnia A, Hoseinzadeh M, Mirlohi M, Feizi A, Salehimehr N, Saee N, Nazari J. The Effect of Neutral Oligosaccharides on Reducing the Incidence of Necrotizing Enterocolitis in Preterm infants: A Randomized Clinical Trial. Int J Prev Med. 2014 Nov;5(11):1387-95. PMID: 25538834; PMCID: PMC4274545. | Wrong intervention |
|  | Fleming P, Wilks M, Eaton S, Panton N, Hutchinson R, Akyempon A, Hardy P, Millar MR, Costeloe K. Bifidobacterium breve BBG-001 and intestinal barrier function in preterm babies: Exploratory Studies from the PiPS Trial. Pediatr Res. 2021 May;89(7):1818-1824. doi: 10.1038/s41390-020-01135-5. Epub 2020 Sep 18. PMID: 32947603. | Different outcome of a study which is excluded as not done in LMIC |
|  | Agrawal S, Rao S, Nathan EA, Patole S. Effect of probiotics on C-reactive protein levels in preterm infants: Secondary analysis of a randomized controlled trial. J Neonatal Perinatal Med. 2018;11(2):165-171. doi: 10.3233/NPM-181763. PMID: 29843270. | Different outcome of a study which is excluded as not done in LMIC |
|  | Jalali, S.Z.; Shiri, M.R.; Shirazi, M.G. Effect of probiotics on full intestinal feeding in premature infants: A double blind, clinical trial. Iran. J. Pediatrics 2020, 30, e100139. | Wrong patient population |
|  | Li Y, Shimizu T, Hosaka A, Kaneko N, Ohtsuka Y, Yamashiro Y. Effects of bifidobacterium breve supplementation on intestinal flora of low birth weight infants. Pediatr Int. 2004 Oct;46(5):509-15. doi: 10.1111/j.1442-200x.2004.01953.x. PMID: 15491374. | Wrong patient population |
|  | Zhu HJ, Cai Y. [Effects of bacillus bifidus supplementation on the immunity in very-low-birth-weight infants]. Zhongguo Dang Dai Er Ke Za Zhi. 2011 Dec;13(12):944-6. Chinese. PMID: 22172255. | Wrong outcomes; Full text NA, article in Chinese; |
|  | Qiao LX, Zhu WY, Zhang HY, Wang H. Effect of early administration of probiotics on gut microflora and feeding in pre-term infants: a randomized controlled trial. J Matern Fetal Neonatal Med. 2017 Jan;30(1):13-16. doi: 10.3109/14767058.2016.1163674. Epub 2016 Mar 29. PMID: 26956782. | Wrong outcomes |
|  | Hua XT, Tang J, Mu DZ. [Effect of oral administration of probiotics on intestinal colonization with drug-resistant bacteria in preterm infants]. Zhongguo Dang Dai Er Ke Za Zhi. 2014 Jun;16(6):606-9. Chinese. PMID: 24927436. | Wrong patient population |
|  | Xu L, Wang Y, Wang Y, Fu J, Sun M, Mao Z, Vandenplas Y. A double-blinded randomized trial on growth and feeding tolerance with Saccharomyces boulardii CNCM I-745 in formula-fed preterm infants. J Pediatr (Rio J). 2016 May-Jun;92(3):296-301. doi: 10.1016/j.jped.2015.08.013. Epub 2016 Mar 3. PMID: 26946967. | Wrong patient population |
|  | Esaiassen E, Hjerde E, Cavanagh JP, Pedersen T, Andresen JH, Rettedal SI, Støen R, Nakstad B, Willassen NP, Klingenberg C. Effects of Probiotic Supplementation on the Gut Microbiota and Antibiotic Resistome Development in Preterm Infants. Front Pediatr. 2018 Nov 16;6:347. doi: 10.3389/fped.2018.00347. PMID: 30505830; PMCID: PMC6250747. | Wrong study design; wrong patient population |
|  | Patole SK, Keil AD, Nathan E, Doherty D, Esvaran M, Simmer KN, Conway P. Effect of Bifidobacterium breve M-16V supplementation on faecal bifidobacteria in growth restricted very preterm infants - analysis from a randomised trial. J Matern Fetal Neonatal Med. 2016 Dec;29(23):3751-5. doi: 10.3109/14767058.2016.1147554. Epub 2016 Feb 26. PMID: 26821074. | Different outcome of a study which is excluded as not done in LMIC |
|  | Jacobs SE, Hickey L, Donath S ProPremsStudy Groups, et al Probiotics, prematurity and neurodevelopment: follow-up of a randomised trial BMJ Paediatrics Open 2017;1:e000176. doi: 10.1136/bmjpo-2017-000176 | Different outcome of a study which is excluded as not done in LMIC |
|  | Mohan R, Koebnick C, Schildt J, Schmidt S, Mueller M, Possner M, Radke M, Blaut M. Effects of Bifidobacterium lactis Bb12 supplementation on intestinal microbiota of preterm infants: a double-blind, placebo-controlled, randomized study. J Clin Microbiol. 2006 Nov;44(11):4025-31. doi: 10.1128/JCM.00767-06. Epub 2006 Sep 13. PMID: 16971641; PMCID: PMC1698302. | Wrong patient population |
|  | Plummer EL, Danielewski JA, Garland SM, Su J, Jacobs SE, Murray GL. The effect of probiotic supplementation on the gut microbiota of preterm infants. J Med Microbiol. 2021 Aug;70(8):001403. doi: 10.1099/jmm.0.001403. PMID: 34431764; PMCID: PMC8513625. | Different outcome of a study which is excluded as not done in LMIC |
|  | Awad H, Mokhtar H, Imam SS, Gad GI, Hafez H, Aboushady N. Comparison between killed and living probiotic usage versus placebo for the prevention of necrotizing enterocolitis and sepsis in neonates. Pak J Biol Sci. 2010 Mar 15;13(6):253-62. doi: 10.3923/pjbs.2010.253.262. PMID: 20506712. | retracted paper |
|  | Sreenivasa B, Sunil Kumar P, Suresh Babu MT, Ragavendra K. Role of synbiotics in the prevention of necrotizing enterocolitis in preterm neonates: a randomized controlled trial. Int J Contemp Pediatr 2015;2:127-30. | Wrong intervention |
|  | Clinical trial of multistrain probiotics (similac tri-blend) in preterm very low birth weight (vlbw) infants: effects on inflammation and immunomodulation. 2022 Eastern Regional Meeting Journal of Investigative Medicine 2022;70:977-1202.(Conference abstract) | Wrong outcomes |
|  | Havranek T, Al-Hosni M, Armbrecht E. Probiotics supplementation increases intestinal blood flow velocity in extremely low birth weight preterm infants. J Perinatol. 2013 Jan;33(1):40-4. doi: 10.1038/jp.2012.37. Epub 2012 Mar 22. PMID: 22441111. | Different outcome of a study which is excluded as not done in LMIC |
|  | Totsu S, Terahara M, Kusuda S. Probiotics and the development of very low birthweight infants: follow-up study of a randomised trial. BMJ Paediatr Open. 2018 Apr 17;2(1):e000256. doi: 10.1136/bmjpo-2018-000256. PMID: 29687082; PMCID: PMC5911150. | Different outcome of a study which is excluded as not done in LMIC |
|  | Stansbridge EM, Walker V, Hall MA, Smith SL, Millar MR, Bacon C, Chen S. Effects of feeding premature infants with Lactobacillus GG on gut fermentation. Arch Dis Child. 1993 Nov;69(5 Spec No):488-92. doi: 10.1136/adc.69.5_spec_no.488. PMID: 8285751; PMCID: PMC1029590. | Different outcome of a study which is excluded as not done in LMIC |
|  | Güney-Varal İ, Köksal N, Özkan H, Bağcı O, Doğan P. The effect of early administration of combined multi-strain and multi-species probiotics on gastrointestinal morbidities and mortality in preterm infants: A randomized controlled trial in a tertiary care unit. Turk J Pediatr. 2017;59(1):13-19. doi: 10.24953/turkjped.2017.01.003. PMID: 29168358. | Wrong intervention |
|  | Chi C, Xue Y, Liu R, Wang Y, Lv N, Zeng H, Buys N, Zhu B, Sun J, Yin C. Effects of a formula with a probiotic Bifidobacterium lactis Supplement on the gut microbiota of low birth weight infants. Eur J Nutr. 2020 Jun;59(4):1493-1503. doi: 10.1007/s00394-019-02006-4. Epub 2019 Jun 13. PMID: 31197506. | Wrong patient population |
|  | Ren YF, Wang LL. [Effects of probiotics on intestinal bacterial colonization in premature infants]. Zhongguo Dang Dai Er Ke Za Zhi. 2010 Mar;12(3):192-4. Chinese. PMID: 20350428. | Wrong patient population |
|  | Zeber-Lubecka N, Kulecka M, Ambrozkiewicz F, Paziewska A, Lechowicz M, Konopka E, Majewska U, Borszewska-Kornacka M, Mikula M, Cukrowska B, Ostrowski J. Effect of Saccharomyces boulardii and Mode of Delivery on the Early Development of the Gut Microbial Community in Preterm Infants. PLoS One. 2016 Feb 26;11(2):e0150306. doi: 10.1371/journal.pone.0150306. PMID: 26918330; PMCID: PMC4769247. | Wrong patient population |
|  | Hoy-Schulz YE, Jannat K, Roberts T, Zaidi SH, Unicomb L, Luby S, Parsonnet J. Safety and acceptability of Lactobacillus reuteri DSM 17938 and Bifidobacterium longum subspecies infantis 35624 in Bangladeshi infants: a phase I randomized clinical trial. BMC Complement Altern Med. 2016 Feb 2;16:44. doi: 10.1186/s12906-016-1016-1. PMID: 26832746; PMCID: PMC4736167. | Wrong patient population |
|  | Abdulkadir B, Nelson A, Skeath T, Marrs EC, Perry JD, Cummings SP, Embleton ND, Berrington JE, Stewart CJ. Routine Use of Probiotics in Preterm Infants: Longitudinal Impact on the Microbiome and Metabolome. Neonatology. 2016;109(4):239-47. doi: 10.1159/000442936. Epub 2016 Feb 10. PMID: 26859305. | Wrong study design |
|  | van Best N, Trepels-Kottek S, Savelkoul P, Orlikowsky T, Hornef MW, Penders J. Influence of probiotic supplementation on the developing microbiota in human preterm neonates. Gut Microbes. 2020 Nov 9;12(1):1-16. doi: 10.1080/19490976.2020.1826747. PMID: 33095113; PMCID: PMC7588225. | Wrong study design |
|  | Mohan R, Koebnick C, Schildt J, Mueller M, Radke M, Blaut M. Effects of Bifidobacterium lactis Bb12 supplementation on body weight, fecal pH, acetate, lactate, calprotectin, and IgA in preterm infants. Pediatr Res. 2008 Oct;64(4):418-22. doi: 10.1203/PDR.0b013e318181b7fa. PMID: 18552710. | Wrong outcomes |
|  | Tanaka K, Nakamura Y, Terahara M, Yanagi T, Nakahara S, Furukawa O, Tsutsui H, Inoue R, Tsukahara T, Koshida S. Poor Bifidobacterial Colonization Is Associated with Late Provision of Colostrum and Improved with Probiotic Supplementation in Low Birth Weight Infants. Nutrients. 2019 Apr 13;11(4):839. doi: 10.3390/nu11040839. PMID: 31013872; PMCID: PMC6520773. | Wrong study design |
|  | Sinha A, Gupta SS, Chellani H, Maliye C, Kumari V, Arya S, Garg BS, Gaur SD, Gaind R, Deotale V, Taywade M, Prasad MS, Thavraj V, Mukherjee A, Roy M. Role of probiotics VSL#3 in prevention of suspected sepsis in low birthweight infants in India: a randomised controlled trial. BMJ Open. 2015 Jul 10;5(7):e006564. doi: 10.1136/bmjopen-2014-006564. PMID: 26163028; PMCID: PMC4499724. | Wrong patient population |
|  | Zampieri N, Pietrobelli A, Biban P, Soffiati M, Dall'agnola A, Camoglio FS. Lactobacillus paracasei subsp. paracasei F19 in Bell's stage 2 of necrotizing enterocolitis. Minerva Pediatr. 2013 Aug;65(4):353-60. PMID: 24051968. | Wrong patient population |
|  | Indrio F, Riezzo G, Raimondi F, Bisceglia M, Cavallo L, Francavilla R. The effects of probiotics on feeding tolerance, bowel habits, and gastrointestinal motility in preterm newborns. J Pediatr. 2008 Jun;152(6):801-6. doi: 10.1016/j.jpeds.2007.11.005. Epub 2007 Dec 26. PMID: 18492520. | Wrong outcomes |
|  | Sinha AP, Gupta SS, Poluru R, Raut AV, Arora NK, Pandey RM, Sahu AR, Bethou A, Sazawal S, Parida S, Bavdekar A, Saili A, Gaind R, Kapil A, Garg BS, Maliye C, Jain M, Mahajan KS, Dhingra P, Pradhan KC, Kawade AS, Nangia S, Mukherjee A, Rasaily R, Sharma RS; ProSPoNS study Group. Evaluating the efficacy of a multistrain probiotic supplementation for prevention of neonatal sepsis in 0-2-month-old low birth weight infants in India-the "ProSPoNS" Study protocol for a phase III, multicentric, randomized, double-blind, placebo-controlled trial. Trials. 2021 Apr 1;22(1):242. doi: 10.1186/s13063-021-05193-w. PMID: 33794969; PMCID: PMC8017823. | Wrong patient population |
|  | Cui X, Shi Y, Gao S, Xue X, Fu J. Effects of Lactobacillus reuteri DSM 17938 in preterm infants: a double-blinded randomized controlled study. Ital J Pediatr. 2019 Nov 9;45(1):140. doi: 10.1186/s13052-019-0716-9. PMID: 31706331; PMCID: PMC6842458. | Wrong patient population |
|  | Ali, F.; Leach, S.; Wang, A.; Lay, C.; Lui, K. The influence of feeding and probiotics on microbiota acquisition of very premature infants during the first year of life. J Paediatr Child Health, 2019, 55: 3-3. https://doi.org/10.1111/jpc.14409_1 | Wrong study design |
|  | Mansoor Aslamzai, Abdul Rahim Raheen, Mohammad Farouq Hamidi et al. The Effect of Dual Strain Probiotic on the Growth and Feeding Intolerance in Premature Neonates of Kabul city: A Randomized Clinical Trial, 17 November 2020, PREPRINT (Version 1) available at Research Square [https://doi.org/10.21203/rs.3.rs-102536/v1] | Wrong patient population |
|  | Zhang AM, Sun ZQ, Zhang LM. Mosapride combined with probiotics on gastrointestinal function and growth in premature infants. Exp Ther Med. 2017 Jun;13(6):2675-2680. doi: 10.3892/etm.2017.4340. Epub 2017 Apr 13. PMID: 28587329; PMCID: PMC5450587. | Wrong intervention |
|  | Jalali S Z , shiri M R , Ghassab Shirazi M. Effect of Probiotics on Full Intestinal Feeding in Premature Infants: A Double Blind, Clinical Trial. Iran J Pediatr.30(3):e100139. doi: 10.5812/ijp.100139. | Wrong outcomes |
|  | Dongol Singh S S , Klobassa DS , Resch B , Urlesberger B , Shrestha RP . Placebo Controlled Introduction of Prophylactic Supplementation of Probiotics to Decrease the Incidence of Necrotizing Enterocolitis at Dhulikhel Hospital in Nepal. Kathmandu Univ Med J (KUMJ). 2017 Oct.-Dec.;15(60):319-323. PMID: 30580349. | Wrong patient population |
|  | De Pipaón, M.S.; Sanz-Gadea, B.M.; Palomino, E.E.; Bueno, M.T.M.; Manzano, S.; Espinosa-Martos et al. Influence of a combination of Lactobacillus and Bifidobacterium strains in the early colonization of preterm infant' gastrointestinal tract. 6th World Congress of PGHAN: Abstracts. Journal of Pediatric Gastroenterology and Nutrition: May 2021 - Volume 72 - Issue - p 1-1313 doi: 10.1097/MPG.0000000000003177 (Conference abstract) | Wrong outcomes |
|  | Samara J, Moossavi S, Alshaikh B, Ortega VA, Pettersen VK, Ferdous T, Hoops SL, Soraisham A, Vayalumkal J, Dersch-Mills D, Gerber JS, Mukhopadhyay S, Puopolo K, Tompkins TA, Knights D, Walter J, Amin H, Arrieta MC. Supplementation with a probiotic mixture accelerates gut microbiome maturation and reduces intestinal inflammation in extremely preterm infants. Cell Host Microbe. 2022 May 11;30(5):696-711.e5. doi: 10.1016/j.chom.2022.04.005. PMID: 35550672. | Wrong outcome |
|  | Ren B. Preventive effect of Bifidobacterium tetravaccine tablets in premature infants with necrotizing enterocolitis. Journal of pediatric pharmacy, 2010, 16(2), 24‐25 | Wrong patient population |
|  | Kaban RK, Wardhana, Hegar B, Rohsiswatmo R, Handryastuti S, Amelia N, Muktiarti D, Indrio F, Vandenplas Y. Lactobacillus reuteri DSM 17938 Improves Feeding Intolerance in Preterm Infants. Pediatr Gastroenterol Hepatol Nutr. 2019 Nov;22(6):545-553. doi: 10.5223/pghn.2019.22.6.545. Epub 2019 Nov 7. PMID: 31777720; PMCID: PMC6856506. | Wrong patient population |
|  | Bayani G, Mafinezhad S, Ehteshammanesh H, Sharifian E, Esmati M, Akbarian Sanavi M, Mollazadeh S. Effect of Probiotics on Enteral Milk Tolerance and Prevention of Necrotizing Enterocolitis in Preterm Neonates. Iranian Journal of Neonatology. 2021 Apr: 12(2). DOI: 10.22038/ijn.2021.51421.1909 | Wrong patient population |
|  | Hussain M, Jabeen S, Subhani R U H. Role of probiotics in prevention of nectrotizing enterocolitis in preterm low birth weight neonates. PJMHS Vol. 10, NO. 2, APR – JUN 2016; 455-459 | Wrong patient population |
|  | Wang S Q, Wang Q R. Clinical effects of probiotics in prevention of neonatal necrotizing enterocolitis | Wrong patient population |
|  | Chou IC, Kuo HT, Chang JS, Wu SF, Chiu HY, Su BH, Lin HC. Lack of effects of oral probiotics on growth and neurodevelopmental outcomes in preterm very low birth weight infants. J Pediatr. 2010 Mar;156(3):393-6. doi: 10.1016/j.jpeds.2009.09.051. Epub 2009 Nov 14. PMID: 19914635. | Different outcome of a study which is excluded as not done in LMIC |
|  | Suhair A Othman. Probiotics use in a neonatal unit in Port Sudan / Sudan: This combination is effective and should be part of the routine care. Medical Science, 2018, 22(89), 18-22 | Wrong study design |
|  | Underwood, Mark A. et al. “A Randomized Placebo-controlled Comparison of 2 Prebiotic/Probiotic Combinations in Preterm Infants: Impact on Weight Gain, Intestinal Microbiota, and Fecal Short-chain Fatty Acids.” Journal of Pediatric Gastroenterology and Nutrition 48 (2009): 216–225. | Wrong intervention |
|  | Romeo MG, Romeo DM, Trovato L, Oliveri S, Palermo F, Cota F, Betta P. Role of probiotics in the prevention of the enteric colonization by Candida in preterm newborns: incidence of late-onset sepsis and neurological outcome. J Perinatol. 2011 Jan;31(1):63-9. doi: 10.1038/jp.2010.57. Epub 2010 Apr 22. PMID: 20410904; PMCID: PMC3016918. | Wrong patient population |
|  | Agarwal R, Sharma N, Chaudhry R, Deorari A, Paul VK, Gewolb IH, Panigrahi P. Effects of oral Lactobacillus GG on enteric microflora in low-birth-weight neonates. J Pediatr Gastroenterol Nutr. 2003 Mar;36(3):397-402. doi: 10.1097/00005176-200303000-00019. PMID: 12604982. | Wrong outcomes |
|  | Armanian AM, Sadeghnia A, Hoseinzadeh M, Mirlohi M, Feizi A, Salehimehr N, Torkan M, Shirani Z. The effect of neutral oligosaccharides on fecal microbiota in premature infants fed exclusively with breast milk: A randomized clinical trial. J Res Pharm Pract. 2016 Jan-Mar;5(1):27-34. doi: 10.4103/2279-042X.176558. PMID: 26985433; PMCID: PMC4776544. | Wrong intervention |
|  | van den Berg JP, Westerbeek EA, Bröring-Starre T, Garssen J, van Elburg RM. Neurodevelopment of Preterm Infants at 24 Months After Neonatal Supplementation of a Prebiotic Mix: A Randomized Trial. J Pediatr Gastroenterol Nutr. 2016 Aug;63(2):270-6. doi: 10.1097/MPG.0000000000001148. PMID: 26859091. | Wrong intervention |
|  | Ishizeki S, Sugita M, Takata M, Yaeshima T. Effect of administration of bifidobacteria on intestinal microbiota in low-birth-weight infants and transition of administered bifidobacteria: a comparison between one-species and three-species administration. Anaerobe. 2013 Oct;23:38-44. doi: 10.1016/j.anaerobe.2013.08.002. Epub 2013 Aug 26. PMID: 23988359. | Wrong outcomes |
|  | Kuwelker, K., Langeland, N., Löhr, I.H. et al. Use of probiotics to reduce infections and death and prevent colonization with extended-spectrum beta-lactamase (ESBL)-producing bacteria among newborn infants in Tanzania (ProRIDE Trial): study protocol for a randomized controlled clinical trial. Trials 22, 312 (2021). https://doi.org/10.1186/s13063-021-05251-3 | Wrong patient population |
|  | S. Agrawal, C.F. Pestell, J. Granich, S. Rao, E. Nathan, J.A. Wray, A.J.O. Whitehouse, S. Patole. Difficulties in developmental follow-up of preterm neonates in a randomised-controlled trial of Bifidobacterium breve M16-V — Experience from Western Australia, Early Human Development 2020;151:105165,ISSN 0378-3782, | Different outcome of a study which is excluded as not done in LMIC |
|  | Patole SK, Keil AD, Nathan E, Doherty D, Esvaran M, Simmer KN, Conway P. Effect of Bifidobacterium breve M-16V supplementation on faecal bifidobacteria in growth restricted very preterm infants - analysis from a randomised trial. J Matern Fetal Neonatal Med. 2016 Dec;29(23):3751-5. doi: 10.3109/14767058.2016.1147554. Epub 2016 Feb 26. PMID: 26821074. | Different outcome of a study which is excluded as not LMIC |
|  | Al-Hosni M, Duenas M, Hawk M, Stewart LA, Borghese RA, Cahoon M, et al. Probiotics-supplemented feeding in extremely low-birth-weight infants. *Journal of Perinatology* 2012;**32**(4):253–9. | Study done in USA: Not LMIC |
|  | Alshaikh B, Samara J, Moossavi S, Ferdous T, Soraisham A, Dersch-Mills D, Arrieta MC, Amin H. Multi-strain probiotics for extremely preterm infants: a randomized controlled trial. Pediatr Res. 2022 Mar 21. doi: 10.1038/s41390-022-02004-z. Epub ahead of print. PMID: 35314794. | Study done in Canada: Not LMIC |
|  | Athalye-Jape G, Esvaran M, Patole S, Simmer K, Nathan E, Doherty D, Keil A, Rao S, Chen L, Chandrasekaran L, Kok C, Schuster S, Conway P. Effect of single versus multistrain probiotic in extremely preterm infants: a randomised trial. BMJ Open Gastroenterol. 2022 Feb;9(1):e000811. doi: 10.1136/bmjgast-2021-000811. PMID: 35185013; PMCID: PMC8860036. | Study done in Australia: Not LMIC |
|  | Bin-Nun A, Bromiker R, Wilschanski M, Kaplan M, Rudensky B, Caplan M, Hammerman C. Oral probiotics prevent necrotizing enterocolitis in very low birth weight neonates. J Pediatr. 2005 Aug;147(2):192-6. doi: 10.1016/j.jpeds.2005.03.054. PMID: 16126048. | Study done in Israel: Not LMIC |
|  | Chrzanowska-Liszewska D, Seliga-Siwecka J, Kornacka MK. The effect of Lactobacillus rhamnosus GG supplemented enteral feeding on the microbiotic flora of preterm infants-double blinded randomized control trial. Early Hum Dev. 2012 Jan;88(1):57-60. doi: 10.1016/j.earlhumdev.2011.07.002. Epub 2011 Nov 4. PMID: 22055271. | Study done in Poland: Not LMIC |
|  | Costeloe K, Hardy P, Juszczak E, Wilks M, Millar MR; Probiotics in Preterm Infants Study Collaborative Group. Bifidobacterium breve BBG-001 in very preterm infants: a randomised controlled phase 3 trial. Lancet. 2016 Feb 13;387(10019):649-660. doi: 10.1016/S0140-6736(15)01027-2. Epub 2015 Nov 28. PMID: 26628328. | Study done in UK: Not LMIC |
|  | Costalos C, Skouteri V, Gounaris A, Sevastiadou S, Triandafilidou A, Ekonomidou C, Kontaxaki F, Petrochilou V. Enteral feeding of premature infants with Saccharomyces boulardii. Early Hum Dev. 2003 Nov;74(2):89-96. doi: 10.1016/s0378-3782(03)00090-2. PMID: 14580749. | Study done in Greece: Not LMIC |
|  | Dani C, Biadaioli R, Bertini G, Martelli E, Rubaltelli FF. Probiotics feeding in prevention of urinary tract infection, bacterial sepsis and necrotizing enterocolitis in preterm infants. A prospective double-blind study. Biol Neonate. 2002 Aug;82(2):103-8. doi: 10.1159/000063096. PMID: 12169832. | Study done in Italy: Not LMIC |
|  | Fujii T, Ohtsuka Y, Lee T, Kudo T, Shoji H, Sato H, Nagata S, Shimizu T, Yamashiro Y. Bifidobacterium breve enhances transforming growth factor beta1 signaling by regulating Smad7 expression in preterm infants. J Pediatr Gastroenterol Nutr. 2006 Jul;43(1):83-8. doi: 10.1097/01.mpg.0000228100.04702.f8. PMID: 16819382. | Study done in Japan: Not LMIC |
|  | Hays S, Jacquot A, Gauthier H, Kempf C, Beissel A, Pidoux O, Jumas-Bilak E, Decullier E, Lachambre E, Beck L, Cambonie G, Putet G, Claris O, Picaud JC. Probiotics and growth in preterm infants: A randomized controlled trial, PREMAPRO study. Clin Nutr. 2016 Aug;35(4):802-11. doi: 10.1016/j.clnu.2015.06.006. Epub 2015 Jul 16. PMID: 26220763. | Study done in France: Not LMIC |
|  | Hikaru U, Koichi S, Yayoi S, Hiromichi S, Hiroaki S, Yoshikazu O. Bifidobacteria prevents preterm infants from developing infection and sepsis. International Journal of Probiotics and Prebiotics 2010;5(1):33-6. | Study done in Japan: Not LMIC |
|  | Indrio F, Riezzo G, Tafuri S, Ficarella M, Carlucci B, Bisceglia M, Polimeno L, Francavilla R. Probiotic Supplementation in Preterm: Feeding Intolerance and Hospital Cost. Nutrients. 2017 Aug 31;9(9):965. doi: 10.3390/nu9090965. PMID: 28858247; PMCID: PMC5622725. | Study done in Italy: Not LMIC |
|  | Jacobs SE, Tobin JM, Opie GF, Donath S, Tabrizi SN, Pirotta M, et al. Probiotic effects on late-onset sepsis in very preterm infants: a randomized controlled trial. Pediatrics 2013;132(6):1055-62. [DOI: 10.1542/peds.2013-1339] [PMID: 24249817] | Study done in Australia/ New Zealand: Not LMIC |
|  | Kanic Z, Turk DM, Burja S, Kanic V, Dinevski D. Influence of a combination of probiotics on bacterial infections in very low birthweight newborns. Wiener Klinische Wochenschrift 2015;127:S210-5. [DOI: 10.1007/s00508-015-0845-0] [DOI: 26373743] | Study done in Slovenia: Not LMIC |
|  | Kitajima H, Sumida Y, Tanaka R, Yuki N, Takayama H, Fujimura M. Early administration of Bifidobacterium breve to preterm infants: randomised controlled trial. Archives of Disease in Childhood. Fetal and Neonatal Edition 1997;76(2):F101-7. [DOI: 10.1136/fn.76.2.f101] [PMID: 9135288] | Study done in Japan: Not LMIC |
|  | Chou IC, Kuo HT, Chang JS, Wu SF, Chiu HY, Su BH, et al. Lack of effects of oral probiotics on growth and neurodevelopmental outcomes in preterm very low birth weight infants. Journal of Pediatrics 2010;156(3):393-6. [DOI: 10.1016/j.jpeds.2009.09.051] [PMID: 19914635] | Study done in Taiwan: Not LMIC |
|  | Lin HC, Hsu CH, Chen HL, Chung MY, Hsu JF, Lien RI, et al. Oral probiotics prevent necrotizing enterocolitis in very low birth weight preterm infants: a multicenter, randomized, controlled trial. Pediatrics 2008;122(4):693–700. [DOI: 10.1542/ peds.2007-3007] [PMID: 18829790] | Study done in Taiwan: Not LMIC |
|  | Manzoni P, Mostert M, Leonessa ML, Priolo C, Farina D, Monetti C, et al. Oral supplementation with Lactobacillus casei subspecies rhamnosus prevents enteric colonization by Candida species in preterm neonates: a randomized study. Clinical Infectious Diseases 2006;42(12):1735-42. [DOI: 10.1086/504324] [PMID: 16705580] | Study done in Italy : Not LMIC |
|  | Mihatsch WA, Vossbeck S, Eikmanns B, Hoegel J, Pohlandt F. Effect of Bifidobacterium lactis on the incidence of nosocomial infections in very-low-birth-weight infants: a randomized controlled trial. Neonatology 2010;98(2):156–63. [DOI: 10.1159/000280291] [PMID: 20234140] | Study done in Germany: Not LMIC |
|  | Millar MR, Bacon C, Smith SL, Walker V, Hall MA. Enteral feeding of premature infants with Lactobacillus GG. Archives of Disease in Childhood 1993;69(5 Spec No):483-7. [DOI: 10.1136/ adc.69.5_spec_no.483] [PMID: 8285750] | Study done in UK: Not LMIC |
|  | Oshiro T, Nagata S, Wang C, Takahashi T, Tsuji H, Asahara T, et al. Bifidobacterium supplementation of colostrum and breast milk enhances weight gain and metabolic responses associated with microbiota establishment in very-preterm infants. Biomedicine Hub 2019;4(3):1-10. [DOI: 10.1159/000502935] [PMID: 31993433] | Study done in Japan: Not LMIC |
|  | Patole S, Keil AD, Chang A, Nathan E, Doherty D, Simmer K, et al. Effect of Bifidobacterium breve M-16V supplementation on fecal bifidobacteria in preterm neonates - a randomised double blind placebo controlled trial. PLOS One 2014;9(3):e89511. [DOI: 10.1371/journal.pone.008951] [PMID: 24594833] | Study done in Australia: Not LMIC |
|  | Reuman PD, Duckworth DH, Smith KL, Kagan R, Bucciarelli RL, Ayoub EM. Lack of effect of Lactobacillus on gastrointestinal bacterial colonization in premature infants. Pediatric Infectious Disease 1986;5(6):663-8. [DOI: 10.1097/00006454-198611000-00013] [PMID: 3099269] | Study done in USA: Not LMIC |
|  | Rougé C, Piloquet H, Butel MJ, Berger B, Rochat F, Ferraris L, et al. Oral supplementation with probiotics in very low- birth-weight preterm infants: a randomized,double-blind, placebo-controlled trial. American Journal of Clinical Nutrition 2009;89(6):1828-35. [DOI: 10.3945/ajcn.2008.26919] [PMID: 19369375] | Study done in France: Not LMIC |
|  | Sadowska-Krawczenko IK, Polak P, Wietlicka-Piszcz A, Szajewska H. Lactobacilllus rhamnosus ATC A07FA for preventing necrotizing enterocolitis in very-low-birth-weight preterm infants: a randomized controlled trial (preliminary results). Polish Journal of Pediatrics 2012;87(2):139-45. | Study done in Poland: Not LMIC |
|  | Stratiki Z, Costalos C, Sevastiadou S, Kastanidou O, Skouroliakou M, Giakoumatou A, et al. The effect of a bifidobacteria supplemented bovine milk on intestinal permeability of preterm infants. Early Human Development 2007;83(9):575–9. [DOI: 10.1016/j.earlhumdev.2006.12.002] [PMID: 17229535] | Study done in Greece: Not LMIC |
|  | Strus M, Helwich E, Lauterbach R, Rzepecka-Węglarz B, Nowicka K, Wilińska M, et al. Effects of oral probiotic supplementation on gut Lactobacillus and Bifidobacterium populations and the clinical status of low-birth-weight preterm neonates: a multicenter randomized, double-blind,placebo- controlled trial. Infection and Drug Resistance 2018;11:1557-71. [DOI: 10.2147/IDR.S166348] [PMID: 30288066] | Study done in Poland: Not LMIC |
|  | Totsu S, Yamasaki C, Terahara M, Uchiyama A, Kusuda S, Probiotics Study Group in Japan. Bifidobacterium and enteral feeding in preterm infants: cluster-randomized trial. Pediatrics International 2014;56(5):714-9. [DOI: 10.1111/ped.12330] [PMID: 24617812] | Study done in Japan: Not LMIC |
|  | Wang C, Shoji H, Sato H, Nagata S, Ohtsuka Y, Shimizu T, et al. Effects of oral administration of bifidobacterium breve on fecal lactic acid and short-chain fatty acids in low birth weight infants. Journal of Pediatric Gastroenterology and Nutrition 2007;44(2):252-7. [DOI: 10.1097/01.mpg.0000252184.89922.5f] [PMID: 17255840] | Study done in Japan: Not LMIC |
|  | Wejryd E, Marchini G, Frimmel V, Jonsson B, Abrahamsson T. Probiotics promoted head growth in extremely low birthweight infants in a double-blind placebo-controlled trial. Acta Paediatrica 2019;108(1):62-9. [DOI: 10.1111/apa.14497] [PMID: 29999201] | Study done in Sweeden : Not LMIC |
