# Supplementary material for: Probiotics for the prevention of mortality and sepsis in preterm very low birth weight neonates from low- and middle-income countries: a Bayesian network meta-analysis
Source: Front Nutr. 2023 Jun 14;10:1133293. doi: 10.3389/fnut.2023.1133293 (PMC10300419; doi:10.3389/fnut.2023.1133293)
Supplement: Supplementary Table 1 — Deviations in the protocol. [file Data_Sheet_1.zip › Supplementary Table 4.docx]

| **Supplementary table 4: Studies awaiting classification** | | |
| --- | --- | --- |
| S No. | Study Reference | Comments |
|  | Lin et al. Early oral probiotics and lactoferrin reduces necrotizing enterocolitis and or late-onset sepsis for preterm very low birth weight infants. | Full text NA |
|  | JPRN-UMIN000023354: Effects of Bifidobacterium breve (BBG-01) administration in Neonatal Intensive Care Unit cared preterm infants to gain enterobacterial flora | Trial protocol; no published results |
|  | Uhlemann M, Heine W, Mohr C, Plath C, Pap S. Wirkungen oral verabreichter Bifidobakterien auf die intestinale Mikroflora von Früh- und Neugeborenen [Effects of oral administration of bifidobacteria on intestinal microflora in premature and newborn infants]. Z Geburtshilfe Neonatol. 1999 Sep-Oct;203(5):213-7. German. PMID: 10596415. | Article in German, NEC is outcome; Full text NA |
|  | Hou et al. Clinical observation of probiotics and baby touch to treat feeding intolerance of premature infant | Full text NA |
|  | Di, M; Li, X . Effects of Bifidobacterium supplementation for prevention of necrotizing enterocolitis in preterm infants: a randomized, controlled trial | Full text and abstract NA |
|  | Ahmad W, Sultan AN, Younas I, Khan MA, Naveed M, Safdar MB. Frequency of necrotizing enterocolitis in preterm infants treated with prophylactic probiotics versus controls. Pakistan journal of medical and health sciences, 2020, 14(3), 929‐931 | No communication from author regarding probiotic strain, mean GA is more but weight is <1500; |
|  | Coleta E, Gheonea M, Sarbu M. Oral supplementation with probiotics in premature infants-a randomised clinical trial. In: Intensive Care Medicine. 24th Annual Meeting of the European Society of Paediatric and Neonatal Intensive Care edition. Vol. 39. Rotterdam, Netherlands, 2013:S113. | Full text NA, conference abstract only |
|  | Punnahitananda S, Thaithumyanon P, Soongsawang K. Nosocomial infection and necrotizing enterocolitis in preterm neonates treated with Lactobacillus acidophilus and Bifidobacterium infantis in a neonatal intensive care unit: a randomized controlled study. In: 14th Congress of the Federation of Asia Oceania Perinatal Societies. Bangkok, Thailand, 2006. | Full text NA, Conference abstract only |
|  | CTRI/2018/04/013401: Role of prophylactic microbial supplements in prevention of blood stream infection and intestinal tract injury in premature neonates | Trial protocol; no published results |
|  | NCT01454661. Probiotics and Early Microbial Contact in Preterm Neonates | Trial protocol; no published results |
|  | CTRI/2018/01/011079: Probiotics for prevention of necrotising enterocoilitis in preterm neonates | Trial protocol; no published results |
|  | CTRI/2017/06/008907: To study the effects of two probiotic strain in preterm infants | Trial protocol; no published results |
|  | NCT01788761: The Effects of Probiotic Supplementation on Extremely Low Birthweight Infants | Trial protocol; no published results |
|  | EUCTR2008-001324-31-IT: Efficacy and security of oral supplementation with a probiotic (Lactobacillus reuteri) in newborns with birth weight < 1500 g: effects on necrotising enterocolitis (NEC) and gut colonization with gram negative bacteria. - NEO 2008-01 | Prematurely terminated; study not published |
|  | IRCT2014072618591N1: Effect of probiotic in prevention of Necroziting Entrocolitis in preterm infants in Hafez hospital | Trial protocol; no published results |
|  | NCT01337921: A Multi-strain Synbiotic Versus a Multi-strain Probiotic in Premature Infants | Withdrawn study protocol; Withdrawn due to safety issues of symbiotic |
|  | ISRCTN99401619: Supplementation of very low birthweight (VLBW) babies with probiotic Bifidobacterium breve strain (BBG): a pilot study of tolerability and feasibility | Trial protocol; no published results |
|  | IRCT2014022716574N3: The effect of probiotics in prevention of necrotizing enterocolitis | Trial protocol; no published results |
|  | JPRN-UMIN000010089: Effect of bifidobacterium enema in very low birth weight infants - double-blind controlled trial of enema vs enteral administration- | Trial protocol; no published result |
|  | IRCT2016061128386N1: Evaluation of the effects of synbiotic supplementation on weight gain, fecal ph, acetate, lactate, calprotectin in preterm infants | Full text NA |
|  | CTRI/2009/091/000556: A clinical trial to study the efficacy of probiotic VSL#3/PP in preventing Necrotizing enterocolitis in preterm infants | Trial protocol; no published result |
|  | CTRI/2018/07/014991: Effect of probiotic supplementation on feed tolerance and weight gain in low birth weight infants on tube feeds | Trial protocol; no published results |
|  | IRCT201210209568N3: The effect of probiotics in premature newborn | Trial protocol; no published results |
|  | NCT02552706: The Efficacy and Mechanisms of Oral Probiotics in Preventing Necrotizing Enterocolitis | Trial protocol; no published results |
|  | NCT04541771: The Role of Lactobacillus Reuteri in Preventing Necrotizing Enterocolitis (NEC) in Pre-term Infants | Trial protocol; no published results |
|  | CTRI/2016/09/007310: To study the efficacy and safety of single strain versus multi strain probiotics in prevention of late-onset sepsis in preterm neonates | Trial protocol; no published results |
|  | IRCT20170121032075N2: The effect of probiotics on prevention of necrotizing enterocolitis in neonates | Trial protocol; no published results |
|  | JPRN-UMIN000034023: A randomised controlled trial of kestose in growth promotion effect of bifodobacteria and safety in premature neonates | Trial protocol; no published results |
|  | JPRN-UMIN000033114: A randomised controlled trial on prevention of colonization of Staphylococcus aureus in neonates using multiple Bifidobacteria | Trial protocol; no published results |
|  | NCT01181791. Effects of Lactobacillus Reuteri in Premature Infants. | prematurely terminated; Terminated, poor recruitment |
|  | ACTRN12618000489291. Live versus heat-inactivated probiotic strains in preterm infants: The Pro-Para study | Trial protocol; no published results |
|  | NCT01375309. Bifidobacterium supplementation for very low birth weight infants (Bifido(RCT)) | Trial protocol; no published results |
