# Supplementary material for: Probiotics for the prevention of mortality and sepsis in preterm very low birth weight neonates from low- and middle-income countries: a Bayesian network meta-analysis
Source: Front Nutr. 2023 Jun 14;10:1133293. doi: 10.3389/fnut.2023.1133293 (PMC10300419; doi:10.3389/fnut.2023.1133293)
Supplement: Supplementary Table 1 — Deviations in the protocol. [file Data_Sheet_1.zip › Supplementary Table 5.docx]

Supplementary Table 5. Characteristics of ongoing studies

| S No | Study reference | Methods | Participants | Interventions | Outcomes | Starting date | Contact information |
| --- | --- | --- | --- | --- | --- | --- | --- |
|  | TCTR20200101001: Effect of probiotics on the incidence of necrotizing enterocolitis in preterm | Double blind randomized controlled trial | Preterm ≤33 wk, ≤1500 g, Able to tolerate feeding at least 1 ml/feed every 3 hours for 8 feeds - Has not received antibiotic for at least 24 hr before enrollment | TS6 probiotics 3 x 10^9^ CFU OD till 34 wks PMA | NEC ≥ stage 2, sepsis, time to full feeds, incidence of sepsis due to probiotic strain. | 21^st^ July, 2019 | Walaiporn Bowornkitiwong; 0894223604; [walbj@hotmail.com](mailto:walbj@hotmail.com) |
|  | Efficacy of Bifidobacterium animalis subsp. lactis (BB-12), B. infantis and Lactobacillus acidophilus (La-5) probiotics to prevent gut dysbiosis in preterm infants of 28+0–32+6 weeks of gestation: a randomised, placebo-controlled, double-blind, multicentre trial: the PRIMAL Clinical Study Protocol BMJ Open 2019;9:e032617. | Double-blind, multicentre clinical RCT | Preterm infants born between 28+0and 32+6 weeks of gestation, within first 48 hrs of birth | Lactobacillus acidophilus, B. lactis and B. infantis; 1.5 X 10^9^ CFU OD till. 28 days or discharge | Gut dysbiosis at Day 28-30, safety outcome: NEC or invasive infection with identification of probiotic bacteria in sterile fluids. | April, 2018 | Professor Christoph Härtel; [christoph.haertel@uksh.de](mailto:christoph.haertel@uksh.de)  (Study is from a high income country) |
|  | IRCT20210102049922N1. Comparison of the effect of probiotic drops pedilact and reuteflor in reducing prematurity complications in premature neonate | Triple-blind randomized controlled trial | Preterm neonates,1-2Kg, <7 days postnatal age | 5 drops of probiotic Pedilact drops made by Iran Zist takhmir Company/ 5 drops of probiotic Reuteflor made by Faradaroo fanavar mehr Iranian Company/ 5 drops of distilled water, daily with breast milk or formula for 7 days. | Duration of hospitalization, time to full feeds, duration of oxygen therapy. Health conditions studied: NEC and neonatal jaundice | 15^th^ June, 2021 | Rezaei Rana; [rezaeir9@mums.ac.ir](mailto:rezaeir9@mums.ac.ir) |
|  | EUCTR2018-000754-22-GB: A randomized, double blind, parallel-group, placebo controlled study to evaluate the efficacy and safety of IBP-9414 in premature infants 500-1500g birth weight in the prevention of necrotizing enterocolitis- - The Connection Study | Double blind randomized controlled trial | Preterm neonates 500-1500 g in first 2 days of life | Infant Bacterial Therapeutics AB (IBT)-9414, oral suspension | Necrotizing enterocolitis, time to sustained feeding intolerance | 4^th^ July, 2019 | Anders Kronström; +46841014555; [clinical@ibtherapeutics.com](mailto:clinical@ibtherapeutics.com)  (Study is from a high income country) |
|  | CTRI/2021/03/031724. Lactobacillus Rhamnosus GG to reduce NEC, sepsis and mortality in VLBW infants –A Randomised Controlled Trial” | Randomized, Parallel Group Trial | Hemodynamically stable preterm <32 wk or <1500 g in first 7 days of life | 6 drops of Lactobacillus rhamnosus GG, started with feed initiation till 35 weeks | Composite outcome of sepsis (blood culture positive sepsis) or NEC Stage II (modified bell staging) or mortality | 10^th^ March, 2021 | Dr Sachin Shah; 9689907296; [sshahdoc@gmail.com](mailto:sshahdoc@gmail.com) |
