# Supplementary material for: Probiotics for the prevention of mortality and sepsis in preterm very low birth weight neonates from low- and middle-income countries: a Bayesian network meta-analysis
Source: Front Nutr. 2023 Jun 14;10:1133293. doi: 10.3389/fnut.2023.1133293 (PMC10300419; doi:10.3389/fnut.2023.1133293)
Supplement: Supplementary Table 1 — Deviations in the protocol. [file Data_Sheet_1.zip › Supplementary Table 6.docx]

Supplementary Table 6: Probiotic used alone or in combination used in the study

| **S No** | **Probiotic alone or in combination** | **Coding** |
| --- | --- | --- |
|  | B lactis | Blac |
|  | Ba clausii | Bacl |
|  | L acidophilus | Lac |
|  | L reuteri | Lreu |
|  | L sporogenes | L spo |
|  | Sa boulardii | Sab |
|  | B bifidum+L acidophilus | BiLac |
|  | B breve+L casei | BrLca |
|  | B infantis+L rhamnosus+L casei+L plantarum+L acidophilus+S thermophilus | BinL4S |
|  | B longum+L rhamnosus | BloLr |
|  | B bifidum+L acidophilus+Sa boulardii | BLSa |
|  | B longum+B bifidum+L plantarum | BBL |
|  | B longum+B bifidum+B lactis+L acidophilus | B3L |
|  | B longum+B bifidum+B infantis+L acidophilus | B3iL |
|  | B longum+L helveticus+L rhamnosus+Sa boulardii | BL2Sa |
|  | B longum+L acidophilus+L rhamnosus+Sa boulardii | BL2aSa |
|  | B spp+L acidophilus+S thermophilus+L delbrueckii | BL2aS |
|  | B bifidum+ B longum+B infantis+L rhamnosus+L paracasei+L casei+L acidophilus+L latis | B3L4 |
|  | B longum+ B breve+L acidophilus+L rhamnosus+L bulgaricus+L casei+S thermophilus | B2L4S |
|  | B spp+L acidophilus+E faecalis | Bado |
|  | L acidophilus+ b infantis+ bacillus cereus+ E fecalis | BLaE |
|  | L paracasei | Lpar |
|  | B infantis + L reuteri + L rhamnosus | BinLreuLrha |
|  | B bifidum + B infantis + L acidophilus | B2La |
