# Supplementary material for: Probiotics for the prevention of mortality and sepsis in preterm very low birth weight neonates from low- and middle-income countries: a Bayesian network meta-analysis
Source: Front Nutr. 2023 Jun 14;10:1133293. doi: 10.3389/fnut.2023.1133293 (PMC10300419; doi:10.3389/fnut.2023.1133293)
Supplement: Supplementary Table 1 — Deviations in the protocol. [file Data_Sheet_1.zip › Supplementary Table 8.docx]

Supplementary Table 8. Summary of confidence rating of various probiotic interventions compared to placebo for sepsis.

| **Comparison** | **Number of studies** | **Within-study bias** | **Reporting bias** | **Indirectness** | **Imprecision** | **Heterogeneity** | **Incoherence** | **Confidence rating** | **Reason(s) for downgrading** |
| --- | --- | --- | --- | --- | --- | --- | --- | --- | --- |
| **B bifidum+L acidophilus:Placebo** | 1 | Major concerns | Some concerns | No concerns | Major concerns | No concerns | Major concerns | Very low | ["Within-study bias","Imprecision","Incoherence"] |
| **B bifidum+L acidophilus+Sa boulardii:Placebo** | 1 | Major concerns | Some concerns | No concerns | Major concerns | No concerns | Major concerns | Very low | ["Within-study bias","Imprecision","Incoherence"] |
| **B breve+L casei:Placebo** | 1 | Some concerns | Some concerns | No concerns | Major concerns | No concerns | Major concerns | Low | ["Imprecision","Incoherence"] |
| **B infantis+L rhamnosus+L casei+L plantarum+L acidophilus+S thermophilus:Placebo** | 1 | No concerns | Some concerns | No concerns | Major concerns | No concerns | Major concerns | Low | ["Imprecision","Incoherence"] |
| **B lactis:Placebo** | 1 | No concerns | Some concerns | No concerns | Major concerns | No concerns | Major concerns | Low | ["Imprecision","Incoherence"] |
| **B longum+ B breve+L acidophilus+L rhamnosus+L bulgaricus+L casei+S thermophilus:Placebo** | 1 | Some concerns | Some concerns | No concerns | Major concerns | No concerns | Major concerns | High | ["Imprecision","Incoherence"] |
| **B longum+B bifidum+B infantis+L acidophilus:Placebo** | 1 | Major concerns | Some concerns | No concerns | No concerns | Major concerns | Major concerns | Very low | ["Within-study bias","Heterogeneity","Incoherence"] |
| **B longum+B bifidum+B lactis+L acidophilus:Placebo** | 1 | Some concerns | Some concerns | No concerns | No concerns | Major concerns | Major concerns | Low | ["Heterogeneity","Incoherence"] |
| **B longum+L acidophilus+L rhamnosus+Sa boulardii:Placebo** | 1 | Major concerns | Some concerns | No concerns | Major concerns | No concerns | Major concerns | Very low | ["Within-study bias","Imprecision","Incoherence"] |
| **B longum+L helveticus+L rhamnosus+Sa boulardii:Placebo** | 2 | Some concerns | Some concerns | No concerns | Major concerns | No concerns | Major concerns | Low | ["Imprecision","Incoherence"] |
| **B longum+L rhamnosus:Placebo** | 1 | No concerns | Some concerns | No concerns | Major concerns | No concerns | Major concerns | Low | ["Imprecision","Incoherence"] |
| **B spp+ L acidophilus+E faecalis:Placebo** | 1 | Some concerns | Some concerns | No concerns | Major concerns | No concerns | Major concerns | Low | ["Imprecision","Incoherence"] |
| **B spp+L acidophilus+S thermophilus+L delbrueckii:Placebo** | 1 | Major concerns | Some concerns | No concerns | Major concerns | No concerns | Major concerns | Very low | ["Within-study bias","Imprecision","Incoherence"] |
| **Ba clausii:Placebo** | 1 | No concerns | Some concerns | No concerns | Major concerns | No concerns | Major concerns | Low | ["Imprecision","Incoherence"] |
| **L paracasei:Placebo** | 1 | No concerns | Some concerns | No concerns | Major concerns | No concerns | Major concerns | Low | ["Imprecision","Incoherence"] |
| **L reuteri:Placebo** | 4 | No concerns | Some concerns | No concerns | Major concerns | No concerns | Major concerns | Low | ["Imprecision","Incoherence"] |
| **L sporogenes:Placebo** | 1 | No concerns | Some concerns | No concerns | Major concerns | No concerns | Major concerns | Low | ["Imprecision","Incoherence"] |
| **Placebo:Sa boulardii** | 2 | No concerns | Some concerns | No concerns | Major concerns | No concerns | Major concerns | Low | ["Imprecision","Incoherence"] |
| **L acidophilus:Placebo** | 0 | Some concerns | Some concerns | No concerns | Major concerns | No concerns | Major concerns | Low | ["Imprecision","Incoherence"] |
