# Supplementary material for: Probiotics for the prevention of mortality and sepsis in preterm very low birth weight neonates from low- and middle-income countries: a Bayesian network meta-analysis
Source: Front Nutr. 2023 Jun 14;10:1133293. doi: 10.3389/fnut.2023.1133293 (PMC10300419; doi:10.3389/fnut.2023.1133293)
Supplement: Supplementary Table 1 — Deviations in the protocol. [file Data_Sheet_1.zip › Supplementary Table 9.docx]

Supplementary table 9: Commonly available probiotic preparations for neonatal use in India

| **Brands** | **Manufacturer** | **Strains** | **Strength** |
| --- | --- | --- | --- |
| Darolac | Aristo | L. acidophilus  L. rhamnosus  B. longus  S. Bulardii | 1.25 * 10^9^ |
| VSL#3 | Alphasigma | Bifidobacterium (3 strains)  Lactobacilus (4 strains)  Streptococcus thermophilus | 112.5 * 10^9^ |
| Enteroplus | GSK | L. rhamnosus GG | 3 * 10^9^ |
| Superflora GG | Sundyota numandis Pharma | L. rhamnosus GG | 6 * 10^9^ |
| Rescunate | Morinaga milk industry | B. breve M16 | 1 * 10^9^ |
| Darolac | Aristo | L. acidophilus  L. rhamnosus  B. longus  S. Bulardii | 1.25 * 10^9^ |
